# Combination of Coevolutionary Information and Supervised Learning Enables Generation of Cyclic Peptide Inhibitors with Enhanced Potency from a Small Data Set

**DOI:** 10.1021/acscentsci.4c01428

**Published:** 2024-11-20

**Authors:** Ylenia Mazzocato, Nicola Frasson, Matthew Sample, Cristian Fregonese, Angela Pavan, Alberto Caregnato, Marta Simeoni, Alessandro Scarso, Laura Cendron, Petr Šulc, Alessandro Angelini

**Affiliations:** † Department of Molecular Sciences and Nanosystems, Ca’ Foscari University of Venice, Via Torino 155, 30172 Mestre, Italy; ‡ School of Molecular Sciences and Centre for Molecular Design and Biomimetics, The Biodesign Institute, Arizona State University, 1001 South McAllister Avenue, Tempe, Arizona 85281, United States; § School for Engineering of Matter, Transport, and Energy, Arizona State University, Tempe, Arizona 85287, United States; ∥ Department of Biology, University of Padua, Viale G. Colombo 3, 35131 Padua, Italy; ⊥ Department of Environmental Sciences, Informatics and Statistics, Ca’ Foscari University of Venice, Via Torino 155, 30172 Mestre, Italy; # European Centre for Living Technology (ECLT), Ca’ Bottacin, Dorsoduro 3911, Calle Crosera, 30123 Venice, Italy; 7 Department of Bioscience − School of Natural Sciences, Technical University of Munich (TUM), Boltzmannstraße 10, 85748 Garching, Germany

## Abstract

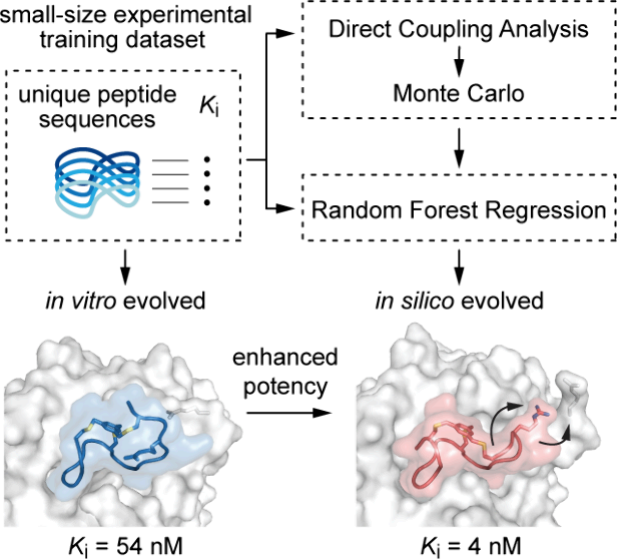

Computational generation of cyclic peptide inhibitors using machine learning models requires large size training data sets often difficult to generate experimentally. Here we demonstrated that sequential combination of Random Forest Regression with the pseudolikelihood maximization Direct Coupling Analysis method and Monte Carlo simulation can effectively enhance the design pipeline of cyclic peptide inhibitors of a tumor-associated protease even for small experimental data sets. Further *in vitro* studies showed that such *in silico*-evolved cyclic peptides are more potent than the best peptide inhibitors previously developed to this target. Crystal structure of the cyclic peptides in complex with the protease resembled those of protein complexes, with large interaction surfaces, constrained peptide backbones, and multiple inter- and intramolecular interactions, leading to good binding affinity and selectivity.

## Abstract 2

Combination of statistical and computational approaches enables rapid and cost-effective generation of potent cyclic peptide inhibitors of a human cancer associated protease.

## Introduction

Cyclic peptides combine numerous favorable properties that make them attractive modalities for drug development.^1^ More than 40 cyclic peptides are used as therapeutics today, with about one cyclic peptide drug approved per year.^2^ The discovery of cyclic peptide ligands with desired binding affinities and specificities has progressed exponentially with the advent of genetically encodable technologies, such as phage display,^3,4^ mRNA display,^5−7^ ribosome display,^8^ bacteria display,^9,10^ yeast display,^11,12^ and the split-intein based approach SICLOPPS.^13^ Although very powerful, such directed evolution approaches are often slow and resource-intensive, as they involve the generation of large combinatorial libraries of random genetically encoded cyclic peptides, multiple iterative cycles of selection, amplification and diversification, and painstaking trial-and-error.^14−16^

In this work, we raised the question of whether the potency of previously selected phage-encoded bicyclic peptide inhibitors could be rapidly and cost-effectively enhanced *in silico* rather than resort to slow, labor-intensive, and pricy *in vitro*, *ex vivo*, and/or *in vivo* evolutionary approaches. Initial attempts to improve the inhibitory potency of a family of bicyclic peptides using a supervised ensemble learning method yielded limited results in terms of prediction. We attributed the poor performance to the small size of the available training data set and attempted an unsupervised statistical learning method. However, even this latter approach proved unable to provide insightful information about the peptide sequence design. We have therefore combined the two approaches and demonstrated that the sequential application of statistical and computational methodologies can effectively enable the rapid and cost-effective *in silico* evolution of chemically constrained bicyclic peptide inhibitors with greater potency than the best previously experimentally evolved *in vitro*. We tested our combined approach on two different families of phage-encoded bicyclic peptide inhibitors of human urokinase-type plasminogen activator (huPA), a cancer-associated trypsin-like serine protease.^17^ In both cases, the *in silico* inferred bicyclic peptides proved to be more potent than the best experimentally evolved inhibitors.

## Results and Discussion

To generate new bicyclic peptide sequences with the desired property, we initially applied machine learning (ML) models on a family of phage-encoded bicyclic peptide inhibitors of huPA, whose most potent inhibitor was named UK18 and had an inhibitory constant (*K*_i_) value of 53 nM.^17^ Further efforts to affinity mature UK18 using phage display and partially randomized combinatorial peptide libraries under stringent selection conditions yielded novel peptide sequences with strong consensus motifs but not improved activities. Identified phage-encoded bicyclic peptides had *K*_i_ values ranging from 53 to 7670 nM.^17^ All bicyclic peptide inhibitors consist of two rings of identical length (each of six amino acids) flanked by three cysteines that have been selectively chemically modified using the same small organic linker 1,3,5-tris(bromomethyl)benzene (TBMB).^18^ Notably, the 3-fold symmetry of this small linker allows the formation of only one isomer upon chemical modification.

Given the small sample size of the training data set available (37 peptide sequences for which we previously measured *K*_i_ experimentally; Supporting data set 1) and the large possible design space (20^*L*^ possible sequences of length *L*), we ruled out using deep learning approaches and instead explored the use of Random Forest Regression (RFR) models to predict *K*_i_. Random Forest is a supervised ensemble learning method based on decision trees.^19^ In the case of regression, numerous decision trees are trained, and the model output is obtained by averaging the outputs of the individual trees.^20,21^ The RFR model was obtained by considering the peptide amino acid sequence information as a feature while also including further properties of the sequence itself in a second phase (Supporting data set 2). The small TBMB linker was not accounted because it does not impose a defined structure to the peptide and does not play a direct role in the binding of the bicyclic peptide to the target protein. Indeed, no noncovalent interactions between the small mesitylene core and the amino acids of the peptide loops (intramolecular) and/or the target proteins (intermolecular) were previously observed.^17,18,22,23^ The main role of this small linker is simply to tie the peptide ends together, leading to reduced flexibility of the backbone. Although entropic contributions are key in binding, they are often difficult to determine and thus to include as features in a training data set, especially for numerous bicyclic peptide molecules. Moreover, the linker remained unchanged in all 37 available bicyclic peptides as did the positions of the three cysteines with which it reacted. The only feature that varies between the different bicyclic peptide inhibitors is, therefore, the composition of the amino acids within the two peptide rings. The resulting RFR model was thus trained exclusively using an amino acid sequence-based data set and tested against bicyclic peptides whose *K*_i_ was known (Supporting results and discussion). However, the predicted *K*_i_ values were affected by a high root mean squared error (RMSE) and model overfitting (Figure S1). The reason for such limited prediction performance probably lies in the small size of the data set used during the training phase.

**Figure 1:**
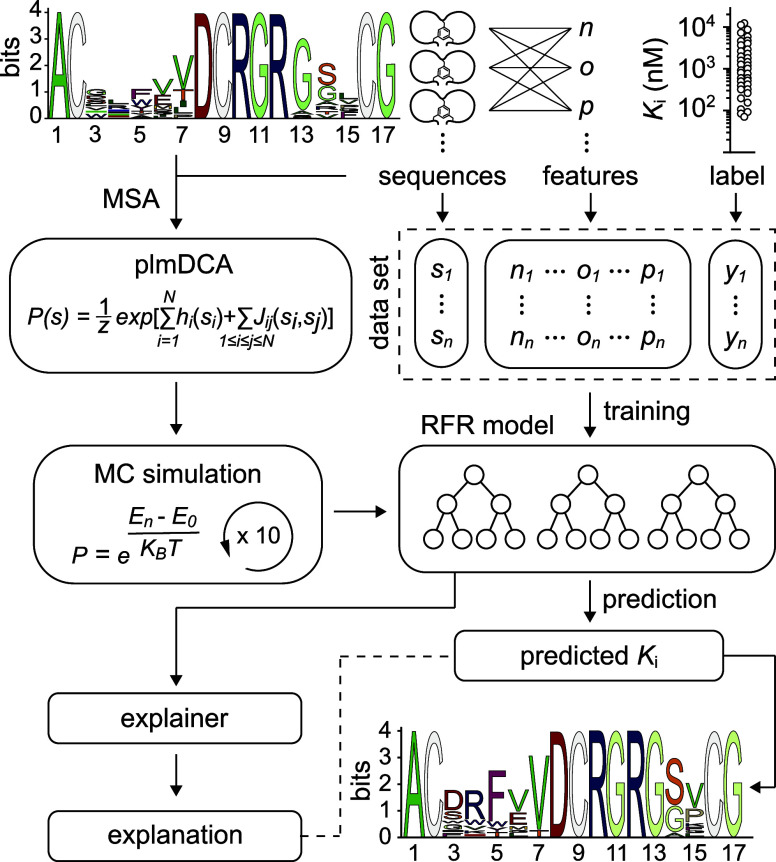
*In silico* molecular evolution of bicyclic peptide inhibitors of huPA. MSA logo of 37 phage-encoded bicyclic peptides (input data) selected *in vitro* against huPA (top left). Training and validation data set were generated using amino acid sequences of all selected bicyclic peptides (“sequences”), their biochemical and biophysical properties (“features”), and the *K*_i_ values (“label”) determined for 37 bicyclic peptide molecules (top right). Combination of pseudolikelihood maximization direct coupling analysis (plmDCA) and Monte Carlo (MC) methods (left) with the Random Forest Regression (RFR) algorithm (right) yielded new peptide sequences with a preferential frequency of amino acids at each position (MSA logo, bottom right; Supplementary Table 1). MSA: multiple sequence alignment; *K*_i_: inhibitory constant.

To overcome the RFR limitations, we applied Direct Coupling Analysis (DCA), an unsupervised statistical learning method that was originally developed^24^ to predict contacts in folded protein structures and has also been recently shown to be able to reconstruct fitness landscape of proteins when trained on sequence alignments obtained from experimental sequence evolution pipeline.^25,26^ The DCA method fits an ansatz to a multiple sequence alignment (MSA), where the parameters *h* and *J* are related to single position conservation and residue covariation (Supporting results and discussion). In particular, herein the MSA of the initial small peptide data set was processed using the pseudolikelihood maximization Direct Coupling Analysis (plmDCA) method,^27^ and the *h* and *J* parameters of the trained model were further used in a Monte Carlo (MC) simulation to sample novel peptide sequences and evaluate the plmDCA model’s score assigned to each sequence (Figure S2). Given the small size of the training data set, it is unlikely for the plmDCA model to learn parameters *h* and *J* such that it would correctly identify all interactions in the family of cyclic peptide binders to a given protein target. However, as described in more detail in the Supporting results and discussion, since the plmDCA is trained with peptide sequences known to bind the target, we expect it can still correctly identify some fraction of the covarying residues in the sequence ensemble. We then randomly generated new sequences with the Monte Carlo sampling algorithm, which uses the plmDCA model score as the effective “energy” parameter and samples the landscape of possible sequences in order to generate new ones with good plmDCA scores. The plmDCA score, trained on such a small data set, is not expected to reflect the actual binding affinity but rather be related to the likelihood that the generated peptide sequence can bind to the protein target.^28^ Based on the plmDCA model’s ability to correctly learn some of the covariations, we expect that at least a few of these generated sequences will also work experimentally. However, it would be too costly to perform a high-throughput experimental scan of all of the sequences generated by the plmDCA model. Therefore, we decided to take advantage of the qualities of both statistical and computational methods and applied them sequentially to generate and select improved peptide inhibitors, respectively. Hence, once fitted to the plmDCA model, MC simulation was used to generate novel sequences (∼23600), that were then given in input to the RFR model to predict their *K*_i_ values (Figure 1 and Table S1).

Finally, the best peptide sequences derived from all of these iterations were selected. Solely bicyclic peptides with *K*_i_ values predicted to be lower than 0.92 μM (that corresponds to 50th percentile) were chosen, resulting in ∼3000 novel sequences. The MSA logo of such peptide sequences revealed a preferential frequency of amino acids at each position that was instructive for the definition of the bicyclic peptides to be tested experimentally (Figure 1 and supporting results and discussion).

**Figure 2:**
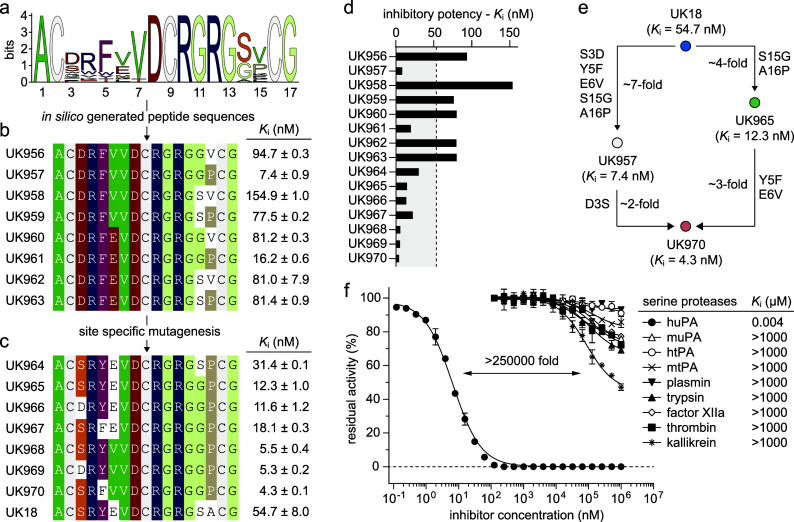
Biochemical characterization of *in silico* evolved bicyclic peptide inhibitors of huPA. a) MSA logo of bicyclic peptides derived from the iterative *in silico* process and predicted to have *K*_i_ values below 0.92 μM (that corresponds to 50th percentile); b) amino acid sequences of bicyclic peptides designed according to the sequence logo graph. The residues with the highest frequency (larger letters) were placed in each position. The sequences are arranged in groups according to sequence similarities; c) amino acid sequences of bicyclic peptides variants in which the *in silico* selected residues were reverted to those present in the parental phage-selected UK18 molecule. Identical or similar amino acids between different bicyclic peptide sequences are highlighted in color. The *K*_i_ values were determined at 25 °C and physiological pH (7.4) using the suitable substrate at the concentration of 50 μM. Mean values of at least three measurements are indicated S.E., standard error; d) column graph comparing the determined *K*_i_ values; e) scheme representing the contribution of mutated amino acid residues to the potency of inhibition; f) residual activities of huPA and a series of homologous human and murine trypsin-like serine proteases incubated with synthetic bicyclic peptide UK970 were determined at 25 °C, at physiological pH (7.4) using the suitable substrates at a concentration of 50 μM. The shown values are the means of three independent experiments. Data are presented as the mean (symbol). S.E., and standard error. The *K*_m_ values of each protease were determined by standard Michaelis–Menten kinetics and used in the calculation of the reported *K*_i_ values (Supplementary Table 2).

Since RFR was trained on a very small set of sequences and its performance was poor, thus increasing the risk of selecting potential false positives from the list of generated sequences, we decided to design the sequences of bicyclic peptide inhibitors to be tested experimentally based primarily on the frequencies of each amino acid residue at each position, as inferred from the MSA logo, rather than relying on the best peptide sequences generated directly by our model. We are indeed aware that while for proteins we can rely on large data sets and defined three-dimensional structures (e.g., the entire Protein Data Bank database) that enable proper training of generative models such as RFdiffusion,^29^ most bicyclic peptides do not have defined structures nor are large structural data available to easily train large-parameter models such as deep learning models.^30,31^ The design of small bicyclic peptide binding proteins must therefore be based on small data sets for which deep learning-based generative models do not have enough information to be trained on to perform accurately. Thus, here we rely on the combination of two models trained on the same small set of bicyclic peptides whose binding affinity is known: a plmDCA model for peptide sequence generation and an RFR model to predict the affinity of the generated peptides. However, due to the small size of the training data set, the best predicted peptide sequences require careful interpretation by experts to obtain functional molecules. Based on their MSA logo, we designed eight representative bicyclic peptide sequences, in which we included the most frequently predicted amino acids at each position (Figure 1). In cases of uncertainty, we used the 3D structure of the best phage-encoded bicyclic peptide UK18 in complex with huPA to better guide our choices. We therefore placed an Asp in position 3, an Arg in position 4, and a Phe in position 5 (Figure 2). The high frequency of an Arg residue at position 4 did not surprise us, since it was also present in UK18. The same applies to the aromatic residue Phe in position 5, which is very similar to the Tyr present in UK18. We were instead a little more intrigued by the high frequency of the negatively charged residue Asp in place of the polar residue Ser in position 3. We were even more surprised to find that in position 6 the hydrophobic residue Val had a higher frequency than the negatively charged Glu. Indeed, the three-dimensional structure of UK18 in complex with huPA revealed that the side chain of Glu in position 6 is crucial in conferring structural rigidity to the bicyclic peptide, since it forms an intramolecular salt bridge with the side chain of the Arg in position 4.^17^ We hence speculated that substitution of a Glu with a Val may have a significant effect on the structure and binding affinity of the bicyclic peptide. So, to assess the contribution of these two residues, we decided to design peptide sequences that include either a Val or a Glu in position 6 (Figure 2). The seven central residues (positions 7 to 13, Val-Asp-Cys-Arg-Gly-Arg-Gly) were instead kept unaltered as they occurred at very high frequency and were shown to be key in conferring high inhibitory potency (Figure 2). At position 14 we placed either a Gly or Ser residue, since they occurred with a significantly higher frequency than other amino acids (Figure 2). Notably, the three-dimensional structure of UK18 in complex with huPA showed that hydroxyl group of Ser in position 14 engages in hydrogen bonds with main carboxyl groups of the nearby Cys (position 9) and Gly (position 11) residues.^17^ Therefore, replacement of a Ser with a Gly is expected to have a significant effect on both the structure and binding affinity of the bicyclic peptide. Finally, in position 15 we included either a Val or a Pro residue (Figure 2). The high frequency of a moderately sized aliphatic hydrophobic amino acid such as Val did not surprise us, as it is quite similar to the Ala residue present in UK18. Conversely, the large incidence of a Pro was unexpected because of both its unique structural properties and its closed vicinity to the last cysteine. To investigate the role of these two residues, we therefore designed peptide sequences that present either a Val or a Pro in position 15 (Figure 2).

Eight designed peptides were chemically synthesized, cyclized with TBMB, and purified by reversed-phase high performance liquid chromatography, the molecular mass determined by electrospray ionization mass spectrometry, and their inhibitory potency assessed using a fluorogenic-based enzyme assay (Figures S3 and S4). Bicycle peptides inhibited huPA with *K*_i_’s ranging from 7.4 to 154.9 nM (Figure 2 and Figure S4). Notably, the concomitant presence of Gly14 and Pro15 in the second loop appears to have a synergic effect. Indeed, peptides UK957 and UK961 revealed *K*_i_ values of 7.4 nM and 16.2 nM, respectively, about 10- and 4-fold better than the best selected phage-encoded bicyclic peptide UK18 targeting huPA (*K*_i_ = 53 nM; Figure 2).^17^

To assess the contribution of the enriched amino acids in the first loop, we generated seven novel bicyclic peptide variants in which the *in silico* selected residues Asp3, Phe5, and Val6 were reverted to those present in the parental phage-encoded bicyclic peptide UK18 molecule, while the Gly14 and the Pro15 of the second loop, respectively, were kept unaltered (Figure S5). Synthetic bicycle peptide variants including either a single or a double amino acid substitution showed *K*_*i*_ values ranging from 4.27 to 31.4 nM (Figure 2 and Figure S6). UK964, which differs from UK18 for the presence of a Pro in place of an Ala in position 15, showed about 1.7-fold enhancement in potency. Further reversion of Ser14 to Gly led to UK965, a peptide variant with a 4-fold increase in inhibitory activity over UK18 (Figure 2 and Figure S6). While the sole or concomitant replacement of the Asp3 to a Ser and the Phe5 to a Tyr yielded peptide variants (UK961, UK965, UK966, UK967) with marginal improvements (*K*_i_’s ranging from 11.6 to 18.1 nM), the reversion of the Glu6 to a Val resulted in three peptide variants (UK968, UK969, and UK970) that are at least 10-fold better than the best selected phage-encoded bicyclic peptide UK18, with UK970 being the most potent one (*K*_i_ = 4.3 nM; Figure 2 and Figure S6).

To assess the specificity of UK970, we determined its *K*_i_’s toward a panel of structurally and functionally related human and murine trypsin-like proteases. The panel included murine uPA, human, and mouse tissue-type plasminogen activators (tPA) as well as other paralogue serine proteases such as the human trypsin, thrombin, plasmin, plasma kallikrein, and factor XIIa (Figure 2 and Table S2). Analogously to the parental clone UK18, the affinity matured bicyclic peptide UK970 appears to be highly specific for huPA (>250000-fold selectivity) as it only weakly inhibits (*K*_i_ > 1 mM) the other homologue enzymes (Figure 2). The high binding specificity of UK970 for huPA is particularly important, as many of the homologue serine proteases tested have vital biological functions, and their inhibition could cause severe off-target side effects.

**Figure 3:**
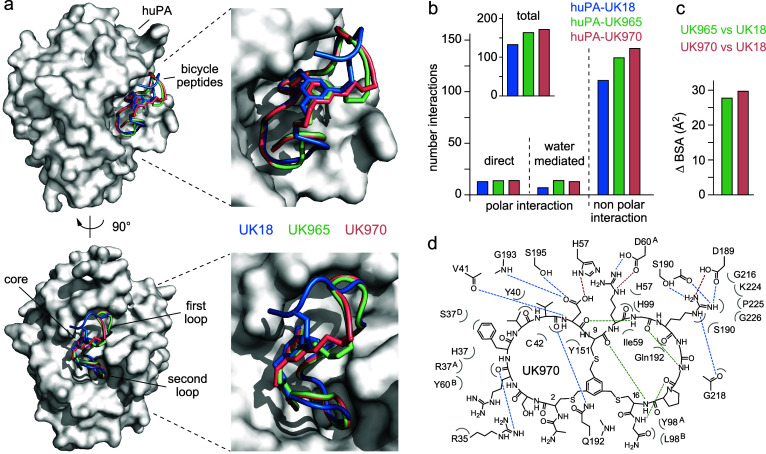
Structural comparison of the binding mode of bicyclic peptides UK18, UK965 and UK970 in complex with huPA. a) Molecular surface representation of the overall huPA-UK18, huPA-UK965, and huPA-UK970 superimposed complexes are shown in two orientations (90° rotation). Surface of huPA is colored in gray, while the peptide ribbon and mesitylene scaffold of UK18, UK965, and UK970 are colored in blue, pale green, and salmon, respectively; b) column graph reporting the total number of polar (both direct and H_2_O-mediates) and nonpolar interactions of huPA with bicycle peptides UK18 (blue), UK965 (pale green) and UK970 (salmon); c) comparative analysis of the buried surface area (BSA) covered by UK965 in respective to UK18 (pale green) and that covered by UK970 in respective to UK18 (salmon); d) schematic representation of molecular interactions between huPA and UK970. Residues of huPA are labeled according to the chymotrypsin numbering system. Intermolecular salt bridges and hydrogen bonds are shown as red and blue dashed lines, respectively. Bicyclic peptide intramolecular hydrogen bonds are shown as green dashed lines. Bent gray lines indicate residues of UK970 in close contact with human uPA (distances shorter than 4.0 Å that are not polar intermolecular interactions).

To unveil the contribution of the different enriched key residues, we applied X-ray crystallography and determined the structure of huPA in complex with bicyclic peptides UK965 (PDB entry 7ZRR) and UK970 (PDB entry 7ZRT) at 1.64 and 1.8 Å maximum resolution, respectively (Figure 3 and Table S3). Overall, superposition of huPA-UK18 (PDB entry 3QN7), huPA-UK965 and huPA-UK970 crystal structure complexes did not show any striking rearrangements of the main huPA backbone with root-mean-square deviations of the Cα-atoms that never exceed 1 Å except for loops Arg37^A^-Ser37^D^ and Leu203-Gly205 (Figure S7). The electron density of UK965 and UK970 peptide chains could be traced unambiguously apart for the first N-terminal residue Ala1 that is not detectable, suggesting some flexibility of orientation inside the crystal (Figure S8). Further comparison of the structure of huPA in complex with UK18 with that of bicyclic peptides UK965 and UK970 revealed that all inhibitors are accommodated in the substrate-binding region of huPA (Figure 3). While phage-encoded UK18 covered a total surface area of 730 Å^2^, the *in silico* evolved UK965 and UK970 variants cover a larger surface area (749 Å^2^ for UK965 and 746 Å^2^ for UK970; Figure 3, Table S4 and Figure S9).

Analogously to UK18, both peptide loops of UK965 and UK970 make contacts with the enzyme, establishing multiple noncovalent interactions with surrounding huPA residues (Figure 3, Table S5 and Figure S9), though bicyclic peptides UK965 and UK970 form a greater number of both intermolecular polar and nonpolar contacts than parental UK18 (Figure 3, Tables S5 and S6). Most of polar interactions are mediated by residues Asp8, Arg10 and Arg12 that are conserved between UK18, UK965, and UK970 (Supporting results and discussion, Figure 3 and Table S6) while the majority of nonpolar contacts are mediated by the aliphatic side chain of Arg4, Tyr5 (UK965) or Phe5 (UK970), Val7, Gly11 and Gly17 (Supporting results and discussion, Figure 3 and Table S7).

**Figure 4:**
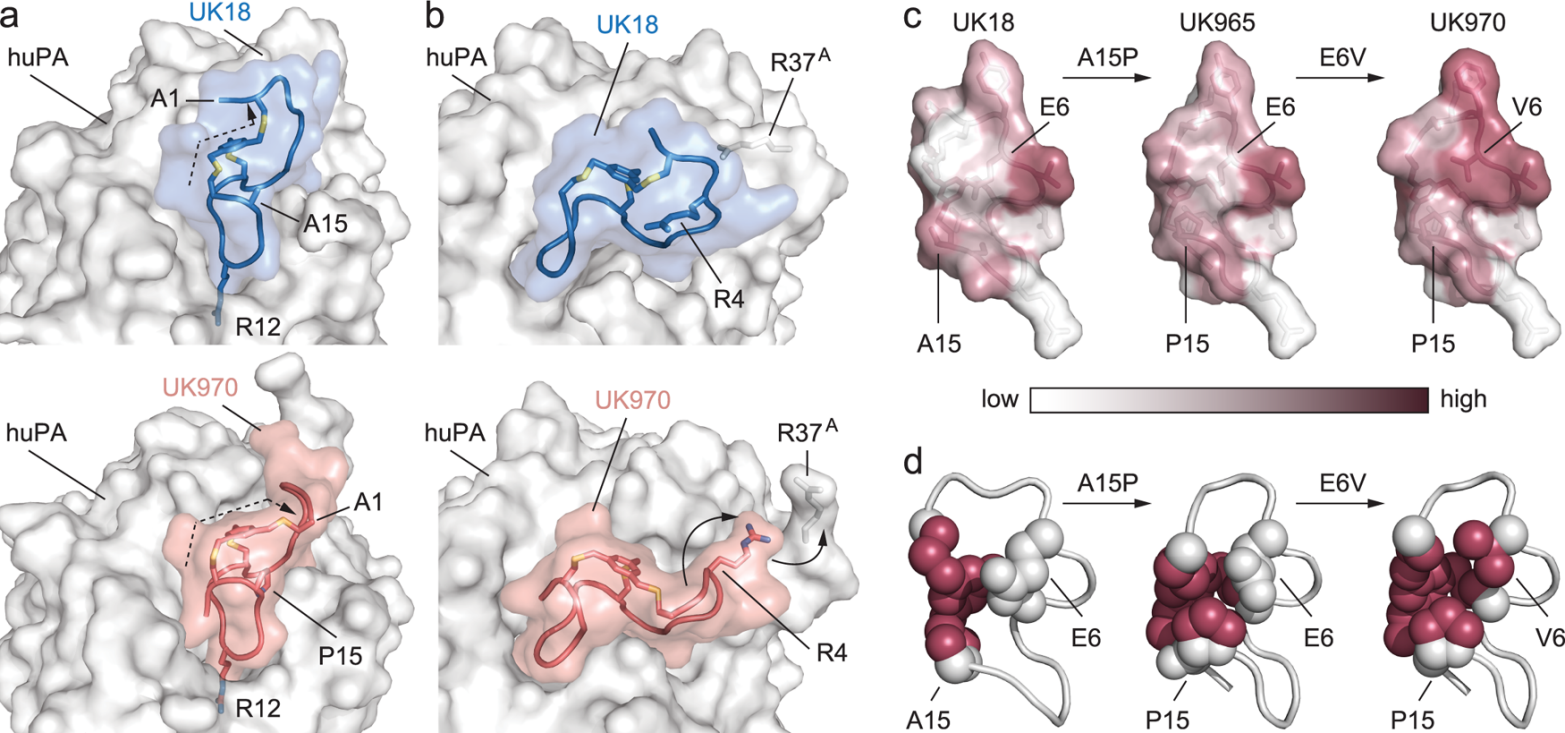
Differences in the binding mode of bicyclic peptides UK965 and UK970 to huPA with respect to UK18. a) Detail view of previously solved X-ray structure of bicyclic peptide UK18 in complex with huPA (blue and gray, top) and bicyclic peptide UK970 in complex with huPA (salmon and gray, bottom). The presence of a Pro instead of an Ala in position 15 of UK970 variant appears to induce a sharp turn in the local geometry that induce a different spatial arrangement of one arm of the linker arm and ultimately impose a different conformation on the backbones of the opposite loop; b) the large conformational change induced by the distal Pro15 cause a repositioning of the Arg4 side chain that instead of forming an intramolecular salt-bridge with Glu6 (top huPA-UK18 complex, gray and blue) now points toward huPA and engages in intermolecular contacts with huPA (bottom huPA-UK970 complex, gray and salmon); c) molecular surface representation of the bicyclic peptides UK18, UK965 and UK970 color-coded according to hydrophobicity. Most hydrophobic residues and the mesitylene scaffold are shown in raspberry, whereas the most hydrophilic ones are shown in white; d) view of the amino acids surrounding the central chemical linker. The mesitylene core and the side chains of the mutated residues are shown as spheres. Hydrophobic residues and the mesitylene scaffold are shown in raspberry, whereas the hydrophilic ones are colored in white.

Major differences in the binding mode of bicyclic peptides UK965 and UK970 to huPA with respect to UK18 can be ascribed to the presence of a Pro instead of an Ala in position 15 (Figure 4a). Hence, the Pro15 located in the second loop of both UK965 and UK970 variants appears to induce a sharp turn in the local geometry that prompts a conformational change of the opposite first loop, ultimately repositioning the amino acid side chains and affecting the interaction with huPA (Figure 4b). The reoriented first loop residues displaced huPA loop Arg37^A^-Ser37^D^ by around 3.5 Å, creating a new binding site that is occupied by Arg4 of UK970 (Figure 4b). Notably, contacts are established with the same shifted huPA residues Arg35 and Arg37^A^, that were not engaged in the huPA-UK18 complex (Tables S6 and S7; Figure S10a,b). The newly induced snug fit of the first loop to the huPA target may explain the higher inhibitory potency of UK965 and UK970 and validated the importance of the skeletal backbone shape that would have been difficult to predict by simply inspecting by eye both the peptide sequence alignments and the crystal structure of the UK18-huPA complex.

However, the higher potency of the bicyclic peptides UK965 and UK970 over the parental UK18 might be attributed not only to a larger contact surface but also to entropy-driven factors. It is fairly well-known that increasing the conformational constrains of the backbone limits entropic penalisation and often leads to better binding properties.^18,32−34^ A major role in the reduction of the conformational freedom appears to be played by both the branched cyclization linker TBMB and the network of noncovalent intramolecular interactions involving side- and/or main-chain atoms of residues of both peptide loops. Indeed, UK965 and UK970 bicyclic peptides exhibit a pattern of intramolecular contacts different from that of UK18 which could further limit the conformational flexibility of their backbone and ultimately provide them with greater compactness and rigidity (Figure 4 and Table S8).

The higher compactness and rigidity of UK965 and UK970 bicyclic peptides in complex with huPA are underpinned by their overall B-factor values, on average lower than that of the parental UK18 (Figure S11a–e). The replacement of the Ala in position 15 with a Pro appears to have a role not only in inducing the conformational change of the first loop but also in squeezing the backbone of the second loop (Figure 4). Indeed, incorporation of proline on a peptide loop is known to impose conformational rigidity.^35,36^ As a result of the presence of Pro15, the two nearby residues Gly11 and Gly14 are brought closer and engage in an intramolecular contact that further increases the overall conformational constraint of the second loop (Figure S11f).

The presence of the central small organic molecule TBMB might not only function as a branching point but also offer an environment to which the surrounding amino acids could adapt to. Indeed, analysis of the hydrophobic profiles of the three bicyclic peptides in complex with huPA revealed that while in the structure of UK18-huPA most of the mesitylene surface was solvent-exposed, in the crystal structure of both UK965-huPA and UK970-huPA complexes the mesitylene group is buried by a patch of aliphatic residues (Val6, Gly14, and Pro15) that seem to pack and fold around the small organic core (Figure 4). Therefore, we cannot exclude that in these specific bicyclic peptide molecules the hydrophobic benzene ring might also function as a nucleating factor that could direct the structure of the peptide moiety by promoting the formation of additional noncovalent interactions between side- and/or main-chain atoms of residues of both peptide loops ultimately leading to a more rigid molecule and thereby a more stable peptide–target complex.^23^ Overall, the compact folding of UK965 and UK970 appears to resemble that of a protein with a central hydrophobic core shaped by the mesitylene moiety and multiple aliphatic residues that wrap around it, whereas the surrounding hydrophilic amino acids are often oriented toward the solvent.

**Figure 5:**
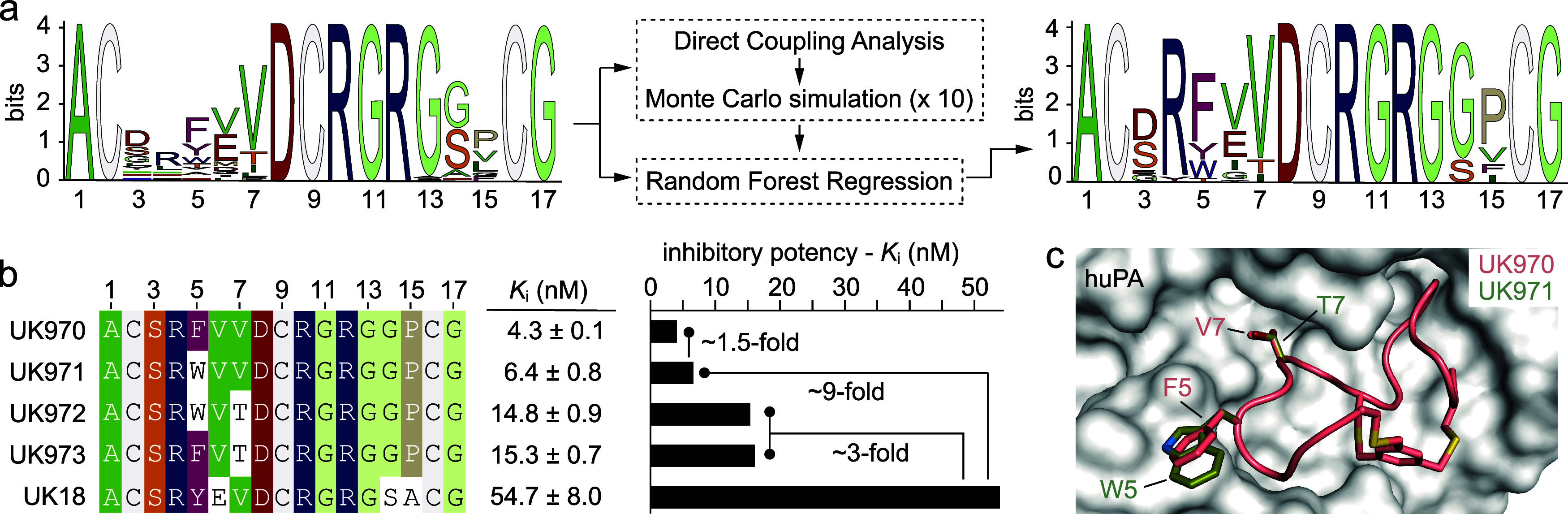
Further round of *in silico* molecular evolution on an enriched family of bicyclic peptide inhibitors of huPA. a) MSA logo of 52 phage-encoded bicyclic peptides (input data) selected *in vitro* against huPA (top left). Combination of pseudolikelihood maximization direct coupling analysis (plmDCA) and Monte Carlo (MC) methods with Random Forest Regression (RFR) algorithm (top middle) yielded new peptide sequences with a preferential frequency of amino acids at each position (MSA logo, top right) and predicted to have *K*_i_ values below 0.38 μM (that corresponds to 50th percentile). b) Left, amino acid sequences of bicyclic peptides designed according to the MSA logo of the new peptide sequences. Identical or similar amino acids between different bicyclic peptide sequences are highlighted in color. Right, column graph comparing the determined *K*_i_ values. The *K*_i_ values were determined at 25 °C and physiological pH (7.4) using the suitable substrate at the concentration of 50 μM. Mean values of at least three measurements are indicated S.E., standard error; c) Structural comparison of the binding mode of bicyclic peptides UK970 and UK971 in complex with huPA. Molecular surface of huPA is colored in gray, while the peptide ribbon and mesitylene scaffold of UK970 and UK971 are colored in salmon and blue, respectively. Selected amino acid side chains (Phe5 and Val7 in UK970; Trp5 and Thr7 in UK971) are represented as ball-and-stick and colored by atom type (carbon = salmon for UK970 and olive for UK971, oxygen = firebrick, nitrogen = deep blue).

Next, we assessed whether our statistical and computational combined approach could be successfully recapitulated on other bicyclic peptide families. To this end, we initially performed a further round of *in silico* molecular evolution using the same 37 phage-encoded bicyclic peptide inhibitors of huPA, to which we added the new 15 peptide sequences generated in this work, to obtain a data set of 52 unique bicyclic peptide molecules (Supporting data set 3). Application of the plmDCA model and MC simulation generated ∼21000 unique sequences that were further selected by RFR resulting in ∼450 novel bicyclic peptide molecules. Solely bicyclic peptide inhibitors with *K*_i_ values predicted to be lower than 0.38 μM (50th percentile) were used to build the MSA logo instructive for the definition of the bicyclic peptides to be tested experimentally (Figure 5a).

Alignment of newly selected peptide sequences confirmed preferential frequency of either an Asp or a Ser in position 3, an Arg in position 4, a Phe or a Tyr in position 5, a Val in position 6, a Gly in position 14, and a Pro in position 15. However, to our surprise, the combinatorial approach continued to pick up a Trp in position 5 and a Thr in position 7, even though these two amino acids were present at a much lower frequency in the enriched data set than in the initial one, since none of the 15 newly added peptide sequences comprised them. Intrigued by the recurrence of these two residues, that we had neglected in the first round of our *in silico* evolution process, we chemically synthesized, purified, and determined the inhibitory potency of three new bicyclic peptide molecules comprising one or both Trp and Thr residues in positions 5 and 7, respectively (Figure S12 and Figure S13). Substitution of Phe in position 5 with a Trp yields UK971, a bicyclic peptide inhibitor that showed a *K*_i_ value of 6.4 nM. Though UK971 was not superior to UK970 (*K*_i_ = 4.3 nM), its power is nevertheless remarkable (only 1.5-fold difference; Figure 5b). Further comparison of the structure of huPA in complex with UK970 with that of the modeled bicyclic peptide UK971 revealed that the site occupied by the aromatic residue Phe can indeed accommodate a Trp well (Figure 5c). Oppositely, replacement of Val in position 7 with Thr was detrimental (UK972, *K*_i_ = 14.8 nM; UK973, *K*_i_ = 15.3 nM; Figure 5b and Figure S13). However, the loss of potency is minimal (<4-fold) and can be explained by the fact that Val and Thr are both branched-chain C-beta amino acids with comparable bulkiness, though Thr contains a hydroxyl group instead of a methyl group in the side chain. Overall, all new generated bicyclic peptide sequences showed inhibitory potencies about 3- and 9-fold better than the best experimentally selected phage-encoded bicyclic peptide targeting huPA (UK18, *K*_i_ = 53 nM; Figure 5 and Figure S13).^17^ These results not only demonstrated the ability of our combined approach to intercept meaningful correlations even from small experimental data sets but also proved the possibility of applying it iteratively. Indeed, by performing sequential cycles of *in silico* evolution on larger data sets fed with new sequences generated at each round, it should be possible to better refine the process and hopefully increase the chances of obtaining more potent molecules.

Furthermore, we applied our *in silico* molecular evolution approach to two new diverse families of phage-encoded bicyclic peptide inhibitors. The first family included bicyclic peptide inhibitors of huPA that had different amino acid sequences than the UK18 family and had a clear consensus motif in the first loop.^17,37^ The second family, on the other hand, comprised bicyclic peptide inhibitors of another serine protease, the human coagulation factor XIIa (hFXIIa), which instead possessed consensus motifs in both loops.^38,39^ While bicyclic peptides of the first family have been generated using the small organic linker TBMB, bicyclic peptides of the second family were obtained using the cyclization linker 1,3,5-triacryloyl-1,3,5-triazinane (TATA).^22,23^ Notably, the linker remains unchanged between cyclic peptide molecules of the same family. Both data sets have a comparable sample size and a similar order of magnitude difference between the highest and lowest measured *K*_i_ values. The first family comprises 31 peptide sequences of length *L* = 17 amino acids and *K*_i_ values ranging from 0.20 to 51.4 μM (250-fold difference between the highest and the lowest *K*_i_ value; Supporting data set 4), while the second family contains 50 peptide sequences of length *L* = 14 amino acids and *K*_i_ values ranging from 0.004 to 3 μM (750-fold difference between the highest and the lowest *K*_i_ value; Supporting data set 5).

To begin, we challenged the system by removing few sequences from the initial experimental training data set, and assessed whether our *in silico* process could indeed generate *de novo* the same removed bicyclic peptide molecules even if it had never encountered them before. To enable good training, yet without biasing the system, we removed two bicyclic peptide inhibitors (<5% of total available molecules) from each initial data set, choosing among the ones that had *K*_i_ values below the 50th percentile and were not the most potent (Figure S14). In the case of the bicyclic peptide inhibitors of huPA, we removed UK115 (*K*_i_ = 610 nM) and UK132 (*K*_i_ = 470 nM), whereas in the case of the bicyclic peptide inhibitors of hFXIIa we eliminated FXII617 and FXII618, both with a *K*_i_ value of 12 nM. The size of the first family thus decreased from 31 to 29 unique sequences, while that of the second family lessened from 50 to 48 unique sequences (Figure S14). In the case of the “depleted” family of bicyclic peptide inhibitors of huPA (29 sequences), application of the plmDCA model and MC simulation generated ∼1700 unique sequences that were further selected by RFR resulting in 63 novel bicyclic peptide molecules with *K*_i_ values predicted to be within 2.16 μM (50th percentile). Interestingly, among the new 63 bicyclic peptide sequences *in silico* generated, we found the initially excluded UK132 molecule (Figure S14a). Similarly, application of plmDCA model and MC simulation using the “depleted” family of bicyclic peptide inhibitors of hFXIIa as training data set (48 sequences) generated ∼230 unique sequences that were further selected by RFR resulting in 6 novel bicyclic peptide molecule with *K*_i_ values expected to be within 0.12 μM (50th percentile). Again, our *in silico* approach proved capable to generate *de novo* the initially excluded FXII617 bicyclic peptide sequence (Figure S14b). These results are remarkable and demonstrate once again the ability of the combined methodology to provide effective peptide sequences even from small experimental data sets.

**Figure 6:**
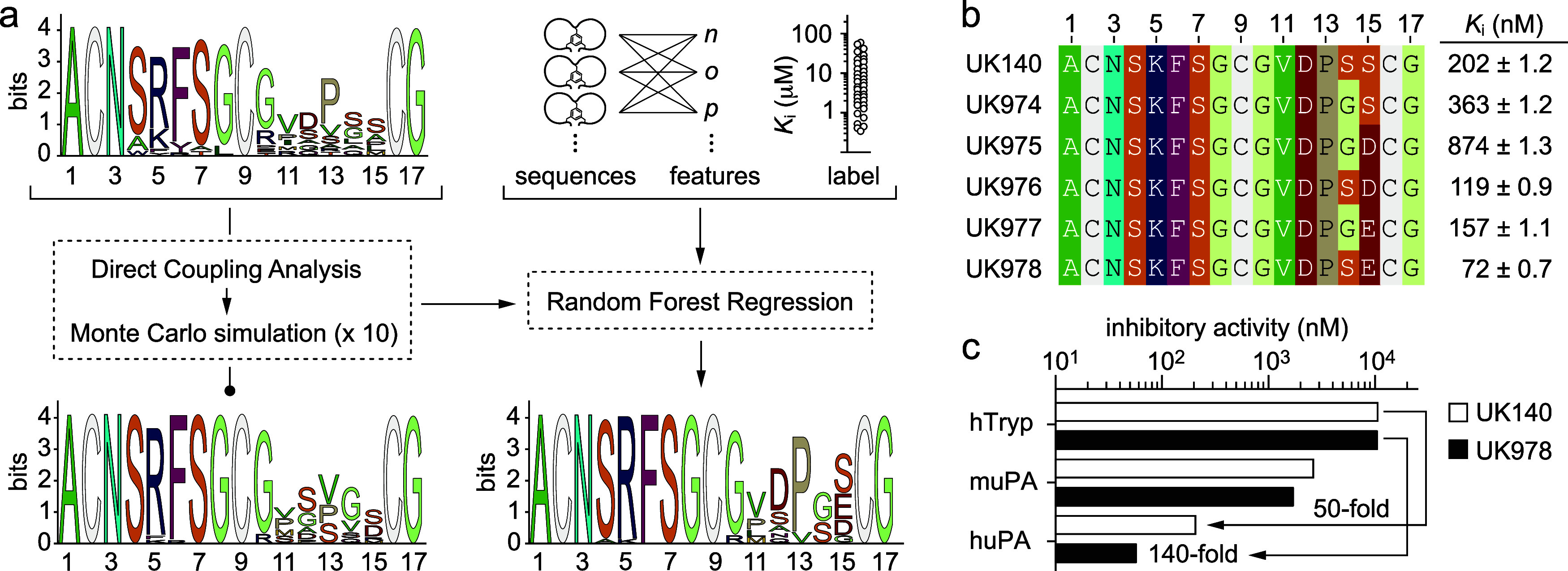
*In silico* molecular evolution on a different family of bicyclic peptide inhibitors of huPA. a) MSA logo of 31 phage-encoded bicyclic peptides (input data) selected *in vitro* against huPA (top left). Training and validation data set were generated using amino acid sequences of all selected bicyclic peptides (“sequences”), their biochemical and biophysical properties (“features”) and the *K*_i_ values (“label”) determined for 31 bicyclic peptide molecules (top right). Combination of pseudolikelihood maximization direct coupling analysis (plmDCA) and Monte Carlo (MC) methods (middle left) with the Random Forest Regression (RFR) algorithm (middle right) yielded new peptide sequences with a preferential frequency of amino acids at each position and predicted to have *K*_i_ values below 1.97 μM (that corresponds to 50th percentile; MSA logo, bottom right). The MSA logo obtained using statistical methods (plmDCA and MC) combined to computational (RFR) algorithm differs from that obtained when applying solely statistical methods (MSA logo, bottom left); b) amino acid sequences and *K*_i_ values of bicyclic peptides UK974–UK978 designed according to the sequence logo graph. Identical or similar amino acids between different bicyclic peptide sequences are highlighted in color. As a reference, the amino acid sequence and *K*_i_ value of the parental phage-selected UK140 are also reported. The *K*_i_ values were determined at 25 °C and physiological pH (7.4) using the suitable substrate at the concentration of 50 μM. Mean values of at least three measurements are indicated S.E., standard error; c) column graph comparing the determined *K*_i_ values of synthetic bicyclic peptide UK140 and UK978 against human uPA (huPA), murine uPA (muPA) and human trypsin (hTryp) proteases. Residual activities were determined at 25 °C, at physiological pH (7.4), using the suitable substrates at a concentration of 50 μM. The shown values are the means of three independent experiments. Data are presented as mean (symbol). S.E., standard error. The *K*_m_ values of each protease were determined by standard Michaelis–Menten kinetics and used in the calculation of the reported *K*_i_ values (Supplementary Table 2).

We then evaluated whether our approach, in addition to generating initially removed sequences, could consistently enable the design of new inhibitors with greater potency than the parental ones. To this end, we exploited the same phage-encoded bicyclic peptide inhibitors of huPA tested above, which included 31 amino acid sequences different from those of the UK18-UK970 family and *K*_i_ values ranging from 0.20 to 51.4 μM. We processed the 31 sequences using the plmDCA model and further used MC simulation to generate novel sequences (∼1700), that were then given in input to the RFR model to predict their *K*_i_ values (Figure 6). Solely bicyclic peptides with *K*_i_ values predicted to be lower than 1.97 μM (that corresponds to 50th percentile) were chosen, resulting in 46 novel sequences. Further multiple sequence alignment of these peptide sequences revealed a more definite occurrence of certain amino acids of the second loop, which was instructive for the design of new bicyclic peptides to be experimentally tested (Figure 6).

While for the design of bicyclic peptides UK956–UK963 against huPA (Figure 2) we could rely on detailed information about the binding mode of parental UK18 in complex with huPA, for this new family of bicyclic peptide inhibitors of huPA we did not have access to structural data to guide us. Therefore, we designed new bicyclic peptide molecules exclusively based on the knowledge collected during the characterization of the 31 initial phage-encoded sequences. We kept the first peptide loop unaltered, except for the residue Arg in position 5, which we replaced with a Lys that proved to be key in enhancing the inhibitory potency (Figure 6b). At positions 11, 12 and 13, we placed the residues Val, Asp, and Pro, respectively, which exhibited not only a higher frequency in the MSA logo but were also comprised in the most potent tested inhibitors (Figure 6b). As for positions 14 and 15, we instead explored all the possible amino acid combinations proposed and designed peptides that included a Gly or a Ser at position 14 and either an Asp, a Glu or a Ser at position 15 (Figure 6b). We must admit that we were particularly intrigued by the high frequency of both negatively charged amino acids, Asp and Glu, at position 15, as they occurred rarely in the phage-encoded sequences, and those few bicyclic peptides that had these residues at position 15 were actually not impressive inhibitors (*K*_i_ > 900 nM).

A total of five new representative peptide sequences were chemically synthesized, cyclized with the small organic linker 1,3,5-tris(bromomethyl)benzene (TBMB), purified by reversed-phase high performance liquid chromatography, the molecular mass determined by electrospray ionization mass spectrometry, and their inhibitory potency assessed using a fluorogenic-based enzyme assay (Figures S15 and S16). The synthetic peptide variants UK974 and UK975, which include a Gly at position 14 and a Ser or an Asp at position 15, showed *K*_i_ values of 363 and 874 nM, respectively, about 1.8- and 4.3-fold worse than the parental UK140 (*K*_i_ = 202 nM; Figure 6 and Figure S16). In contrast, bicyclic peptides UK976 and UK978, which differ from UK140 for the presence of either an Asp or a Glu in place of a Ser in position 15, showed greater inhibitory potency, approximately 1.7- and 2.8-fold higher, respectively (Figure 6 and Figure S16). The favorable effect of the presence of a Glu instead of an Asp in position 15 can also be seen in bicyclic peptide variant UK977, which, despite having a Gly instead of a Ser at position 14, is nonetheless more potent than UK140 (1.3-fold) and UK975 (5.5-fold; Figure 6 and Figure S16). Once again, our *in silico* approach proved capable of recognizing meaningful correlations and instructing the design of valuable bicyclic peptide molecules from small experimental data sets, even in the absence of an informative three-dimensional structure.

While the exquisite specificity of UK970 for human uPA was pleasing (Figure 2f), as many of the paralogous serine proteases tested play key biological functions and their inhibition could cause severe side effects, the sparing of the orthologue murine uPA (muPA) poses difficulties for the testing of the inhibitor in a preclinical mouse model. On the contrary, bicyclic peptide UK140 can also inhibit the orthologue murine uPA (muPA; *K*_i_ = 2.6 μM), though at a low micromolar concentration.^37^ However, UK140 can also weakly block the paralogue human trypsin (hTryp; *K*_i_ = 10.5 μM). We therefore assessed whether UK978, in addition to being more potent than UK140 against huPA, also retained its cross-reactivity for muPA and, hopefully, increased its specificity against hTryp. Indeed, when tested *in vitro*, bicycle peptide UK978 showed higher potency (∼1.5-fold) against muPA (*K*_i_ = 1.7 μM), while that for hTryp remained unchanged (*K*_i_ = 10.4 μM; Figure 6c and Figure S17). The greater potency and retained cross-reactivity of UK978 toward huPA and muPA, combined with its higher specificity toward hTryp (140-fold), are important, as it provides the opportunity to develop a novel and potent human and murine cross-reactive bicyclic peptide inhibitor that, differently from UK970, could be potentially tested in murine models, ultimately allowing not only the evaluation of the therapeutic efficacy but also a better assessment of treatment toxicity, as well as simpler and less costly clinical studies, facilitating the transition from preclinical murine models to human clinical trials.

## Conclusions

In summary, in the present work, we demonstrated that sequential combination of statistical (plmDCA and MC) and computational (RFR) approaches can enable the rapid and cost-effective affinity maturation of chemically constrained bicyclic peptide inhibitors with at least enhanced potency over the best *in vitro* evolved clone. Even though we used these models trained on very small data sets compared to their typical applications in bioinformatics context, they were still able to inform peptide sequences that have been experimentally verified to have higher potency than those used for model training. For example, in the case of the family of 37 phage-encoded bicyclic peptide inhibitors of huPA, whose most potent inhibitor is UK18, by inspecting more closely the parameters *h* and *J* of the trained plmDCA model, we identified that, besides learning the conserved residues from the sequence alignments, the interaction matrix *J* has also shown a high score between several amino acid pairs in specific positions (e.g., Phe and Val in positions 5 and 15, Gly and Val or Gly and Pro in positions 14 and 15 and various amino acid pairs in positions 15–4 and 5–15), which have then biased the generated sequences to contain these pairs. Notably, these amino acids pairs in specific positions have later been verified experimentally to play a key role. Hence, despite the small training set, the models still picked-up correlations that can provide an informed search of design space and perform better than what we would have been able to do just by inspecting by eye the original data set. Though this *in silico* molecular evolution approach has so far been evaluated using two different families, each comprising highly similar bicyclic peptide sequences, our results suggest that sequential application of the plmDCA model and MC simulation combined with the RFR algorithm can effectively enhance the design pipeline even from small experimental data sets that are not suitable for machine learning approaches with large numbers of free parameters such as deep neural networks. Further *in vitro* studies showed that such *in silico*-derived small bicyclic peptides appear to have properties typical of proteins, such as large surface of interaction with the target, constrained peptide backbones, multiple inter- and intramolecular noncovalent interactions mediated by both peptide loops, leading to good binding affinity and specificity. Such exquisite binding features are often difficult to rationalize and can be ascribed to an intricate balance of both enthalpic and entropic factors. We developed this concept with bicyclic peptides against huPA, but these studies also have value as a proof-of-concept for a general approach that could be applied to other relevant peptide binders and protein targets. Although many challenges still remain, the ability to evolve *in silico* cyclic peptide inhibitors using small data sets and a combination of computational and statistical approaches might pave the way for the fast generation of small-mimic proteins with excellent binding affinities and specificities, access to chemical synthesis, and attractive pharmacological properties. Further efforts are underway to implement our approach to include the contribution of linkers with different geometries and chemical groups that could provide different environments and thus impose different conformations to the backbones of bicyclic peptides. Additionally, we are trying to evaluate whether it is possible to vary the positions of the cysteines and consequently the length of the two peptide rings. Although captivating, these are all very challenging topics that will be the subject of future work since they first require the generation of new experimental data sets, even of small size, with which to train our method. Ongoing developments in this direction in the coming years hold promise for further increasing success rates, reducing dependence on extensive experimental optimization. Our results also suggest a possibility of an iterative generative method for design of cyclic peptide inhibitors, where one first trains model with a small number of sequences, generates and experimentally tests a set of them, and uses the experimentally verified binders to augment the data set and retrain the model. This model-experiment driven exploration of possible design space of all sequences can be more cost-effective than screening of a large number of completely random library of sequences.

## Data Availability

The source code and data used to produce the results and analyses presented in this manuscript are available from Open Science Framework (OSF) data repository: https://osf.io/gn6bz/?view_only=20805b7801ba4610a370080e3835fb3c.

## Supporting Information Available

Supplementary results and discussion, materials and methods and supplementary tables and figures. Generation of cyclic peptide sequences using supervised random forest regression. Affinity maturation of cyclic peptide sequences using pseudolikelihood maximization directed coupling analysis and Monte Carlo simulation. HPLC and mass spectrometry analysis of cyclic peptides. Inhibitory activity and specificity of cyclic peptides. Statistics on X-ray diffraction data collection and refinement. Conformation and electron density map of cyclic peptides. Superimposition of protein-peptide complexes. Buried surface in protein and cyclic peptide complexes. Analysis of intermolecular interactions between protein and different cyclic peptides. Directed coupling analysis parameter representations. Receiver operating characteristic and Kullback–Leibler divergence analysis. Python scripts and data sets with input files of cyclic peptide sequences used in the study (PDF)
