# Supplementary material for: Combination of Coevolutionary Information and Supervised Learning Enables Generation of Cyclic Peptide Inhibitors with Enhanced Potency from a Small Data Set
Source: ACS Cent Sci. 2024 Nov 20;10(12):2242–52. doi: 10.1021/acscentsci.4c01428 (PMC11672547; doi:10.1021/acscentsci.4c01428)
Supplement: Supplementary file 1 — oc4c01428_si_001.pdf [file oc4c01428_si_001.pdf]

Combination of co-evolutionary information and supervised learning enables generation of cyclic peptide inhibitors with enhanced potency from a small dataset

Ylenia Mazzocato[1], Nicola Frasson[1], Matthew Sample[2,3], Cristian Fregonese[1], Angela Pavan[4], Alberto Caregnato[1], Marta Simeoni[5,6], Alessandro Scarso[1], Laura Cendron[4], Petr Šulc[2,7], and Alessandro Angelini[1,6]*

[1] Department of Molecular Sciences and Nanosystems, Ca' Foscari University of Venice, Via Torino 155, 30172 Mestre, Italy

[2] School of Molecular Sciences and Centre for Molecular Design and Biomimetics, The Biodesign Institute, Arizona State University, 1001 South McAllister Avenue, Tempe, AZ 85281, USA

[3] School for Engineering of Matter, Transport, and Energy, Arizona State University, Tempe, AZ 85287, USA

[4] Department of Biology, University of Padua, Viale G. Colombo 3, 35131 Padua, Italy

[5] Department of Environmental Sciences, Informatics and Statistics, Ca' Foscari University of Venice, Via Torino 155, 30172 Mestre, Italy

[6] European Centre for Living Technology (ECLT), Ca' Bottacin, Dorsoduro 3911, Calle Crosera, 30123 Venice, Italy

[7] Department of Bioscience – School of Natural Sciences, Technical University of Munich (TUM), Boltzmannstraße 10, 85748 Garching, Germany

* Author to whom correspondence should be addressed: alessandro.angelini@unive.it

Supplementary results and discussion

In silico bicyclic peptide sequence generation using supervised random forest regression

To enhance the binding affinity of bicyclic peptides toward huPA *in silico* we initially applied a machine learning (ML) approach based on supervised Random Forest Regression (RFR) algorithm[1–4] using the Scikit-learn Python library.[5] We first organized both training and validation data in a table (**Supporting dataset 1**). This data set table includes the amino acid sequence of 37 unique phage-encoded bicyclic peptides known to inhibit human urokinase-type plasminogen activator (huPA) with inhibitory constant (K_i) values ranging from 53 to 7670 nM. The data set table has been organized in such a way that each bicyclic peptide sequence was sectioned and placed in columns where each column corresponds to a single amino acid (single letter code). Given that each bicyclic peptide sequence includes 17 residues, such allocation allowed us to obtain a data set table including 37 rows (total number of unique sequences) and 17 columns (total number of amino acids per sequence). An additional column was devoted to the K_i value ("label") experimentally determined for each bicyclic peptide sequence (**Supporting dataset 1**). The amino acid single letter codes were converted into numerical inputs using One Hot Encoding (OHE) approach.[5] To improve analysis performance, additional biochemical and biophysical properties ("features") were extracted for each bicyclic peptide sequence, determined using modlAMP[6] package and further added to generate a new data set table (**Supporting dataset 2**). For each amino acid of each sequence, we added the following properties: molecular weight, charge, charge density, hydrophobicity, isoelectric point, aromaticity, instability index and Boman index. By doing so, an approximately 5-times larger data set table was generated, including eighty-eight features (columns) and thirty-seven unique bicyclic peptide sequences (rows). Best hyperparameters were selected for training and test split size 90%-10% and 80%-20% according to specific metrics such as the root mean square error (RMSE; **Figure S1b** and **S1c**). To test and validate the RFR model we created a set of *N* novel random peptide molecules of length *L* residues by introducing random mutations in the amino acid sequences. Given the large size of the design space (20^{17} unique sequences of length L = 17), we decided to reduce it by keeping the highly conserved residues located in position 1, 2, 8, 9, 10, 11, 12, 13, 16 and 17 unaltered. Only amino acids in position 3, 4, 5, 6, 7, 14 and 15 were allowed to mutate, thus reducing the total space to

explore to 20^7 new potential peptide sequences. We randomly generated 2×10^6 *de novo* created sequences that have been further scored using the RFR model which had been trained on the data set of **Supporting dataset 2**. Bicyclic peptide sequences predicted by RFR to have a K_i < 1.2 μM were collected and used to generate a MSA logo. To explain predictions of the classifier and better appreciate the contribution of each feature we applied local interpretable model-agnostic explanations (LIME)[7] and Shapley Additive exPlanations (SHAP).[8] The predicted K_i values for the novel generated bicyclic peptide sequences were affected by high RMSE and model overfitting (**Figure S1b** and **S1c**). All these issues are likely due to the small size of the dataset used during the training section. Overall, the MSA logo for the bicyclic peptide molecules generated by RFR did not show any prominent motifs in the first loop (**Figure S1d**).

***In silico* bicyclic peptide affinity maturation using pseudolikelihood maximization directed coupling analysis and Monte Carlo simulation**

To overcome the issues encountered by the ML approach based on supervised RFR algorithm, we undertook a different strategy and applied pseudolikelihood maximization directed coupling analysis (plmDCA) combined to Monte Carlo (MC) simulation.[9,10] Briefly, the statistical plmDCA method is based on fitting a Potts model to assign probability to a target peptide sequence *s*:

$$P(s) = \frac{1}{Z} exp\left[\sum_{i=1}^{N} h_i(s_i) + \sum_{1 \le i \le j \le N} J_{ij}(s_i, s_j)\right] \qquad (1)$$

where s_i and s_j correspond to the type of amino acid in position i and j respectively, h corresponds to the conservation of a specific amino acid on a given position, and J_{ij} is a matrix corresponding to pairwise interaction parameters. For each pair of sites i and j, J_{ij} is $q \times q$ square matrix, where q = 21 is the number of possible amino acids (20 natural amino acids plus a gap symbol '-'), and local field $h_i(s_i)$ represents information on how a particular amino acid s_i is conserved in position i of the sequence. The direct coupling analysis (DCA) methods were originally developed to infer direct interactions of amino acids in proteins to guide prediction of their three-dimensional structure based on their co-evolution (**Figure S2a**).[10–12] As opposed to the RFR approach, the plmDCA method does not require additional experimental information (e.g. K_i values) but only necessitates the alignment of bicyclic peptide amino acid sequences. Furthermore, to enlarge the dataset, we used MC sampling

(using the energy assigned to given sequence by a Potts model) to generate novel bicyclic peptides (**Figure S2c**). The MC was applied iteratively (10 cycles), where we used trained Potts model energy to run MC simulation and generate a new augmented set of sequences to be evaluated then by the RFR model (~23600 sequences were generated and evaluated; **Figure S2a)**. Matplotlib and Seaborn Python-based packages were used to generate heat-maps for visualization of the J_{ij} interaction matrices (**Figure S2b**).[12,13] The interaction matrix J is an $L \times L$ tensor, where L is the length of the peptide and each element of the tensor is a submatrix of $q \times q$ dimension in which q = 21 represents the number of possible amino acids for each position (20 natural amino acids plus a gap symbol '-'). **Figure S2b** shows the inferred J tensor where respective entries are norms of submatrices and are classified according to their values in red (high) or blue (low). Therefore, these entries represent interaction matrices given for pairs of amino acids and refer to the probability of obtaining specific amino acid pairs in each coupled position, the higher the values the higher the probability. Overall, the MSA logo for the bicyclic peptide molecules generated by plmDCA did not show any prominent motifs in the first loop (**Figure S2c**).

***In silico* characterisation of plmDCA models' fitting capabilities on small datasets**

In our modelling pipeline, we use a very small set of sequences, and we hence wanted to explore in more details the ability of the DCA model inference to discover correct features from the training dataset. Previous mathematical work[12,14,15] has focused on the theoretical limits of the inference methods for Pott's model (and hence directly relevant to DCA models), quantified by the ability to reconstruct a known interaction matrix J based on the dimensions of the matrix and the number of system observations generated from the Potts model. However, these works typically analytically calculate exact results in limiting cases, and we have hence decided to empirically test the plmDCA model capabilities in an *in silico* model, where we define our own model parameters h and J and generate observed sequences from them, following the distribution as given by equation (1), where $P(s)$ corresponds to a probability of peptide sequence s consisting of amino acids s_1 to s_L. Here, we treated the probability as an effective score, assuming that high probability sequences correspond to strong (good) binders. To assess the plmDCA model's ability to represent a peptide family from just 37 sequences, we developed an *in silico* method to evaluate its fitting capabilities. We began by hardcoding the DCA model parameters, namely the h matrix and J tensor, to

represent a fictitious peptide family (**Figure S18**). Next, we used the Metropolis-Hastings algorithm to sample over 3000 sequences from the hardcoded DCA parameters, generating an *in silico* peptide family dataset. Subsequently, we randomly sampled 37 sequences from the *in silico* peptide family and trained a DCA model on these sequences. After training the DCA model, we used the newly learned *h* and *J* parameters to generate another set of over 3000 peptide sequences using the Metropolis-Hastings algorithm. Using both the hardcoded and learned DCA parameters, and the energy distributions from sequence evaluations, we computed ROC curves (**Figure S19**) and Kullback-Leibler divergences (**Figure S20**). These results demonstrate that a DCA model trained on just 37 sequences from a peptide family effectively captures key properties of the entire family.

To hardcode the DCA model parameters to represent peptide families, we first defined a set of parameters to create various peptide families. The parameters chosen to define the families included: the number of conserved sites, total correlations, amino acids per correlation, and the presence of conserved regions. The number of conserved sites established the *h* matrix (**Figure S18c**). Since our experimental peptide family (**Figure S18a**) had 10 conserved sites, we tested families with 8 or 10 conserved sites. First, we initialized an *h* matrix with dimensions: sequence length (L) × number of possible amino acids (17, 21). For a family with 10 conserved sites, we assigned random amino acids to 10 random locations, each with a value of approximately 1. Specifically, the value set in the *h* matrix for the conserved sites was sampled from a normal distribution with a mean equal to the maximum value of the experimental *h* matrix trained on the 37 experimental sequences (**Figure S18a**), and variance from the same set of values.

The subsequent parameters for the peptide family, used to populate the *J* tensor (**Figure S18d**), were the total number of correlations and the number of amino acids per correlation. The *J* tensor dimensions were: sequence length (L) × sequence length (L) × number of possible amino acids × number of possible amino acids (17, 17, 21, 21). To populate this tensor, we added interactions between non-conserved sites, choosing a total number of pairwise interactions between 15 and 45. For each correlation, we selected 3 or 6 possible amino acids. For example, with 15 total interactions and 3 amino acids per interaction, we randomly selected two non-conserved sites and picked 3 amino acids for each. We then assigned interaction values in the *J* tensor between all possible allowed amino acids for each site pair, resulting in nine non-zero interaction ($a1$, $a2$, $a3$) × ($a4$, $a5$, $a6$) for a chosen interaction site pair. We repeated this process 14 more times (with replacement), filling in

135 elements, with potential repeated site correlations. For a total of 45 correlations and 6 amino acids per correlation, 1620 elements ($45 \times 6 \times 6$) were filled in. The correlation value was sampled from a normal distribution with a mean equal to the maximum value of the experimental J tensor trained on 37 experimental sequences and variance from the same dataset. Finally, we added the amino acids assigned to each correlated site to the h matrix, sampling the self-correlation value from a normal distribution based on the mean and variance derived from the experimental h matrix.

The last parameter for the peptide families was the presence or absence of a conserved region. If there was no conserved region, we hardcoded the h and J matrices as described. However, if a conserved region existed, we used a slightly different approach. When populating the h matrix, after randomly choosing the first conserved site, we selected 4 sequential sites to create a continuous conserved region. The next 5 conserved sites were then chosen randomly. For the J tensor, we selected the longest continuous region of un-conserved sites to be interacting, creating "correlated regions". We generated a total of 72 *in silico* peptide families. Additionally, we created 3 sets of h and J parameters randomly, with each value sampled from a standard normal distribution (**Figure S18b**).

To generate realizations of the peptide families encoded in the DCA model parameters, we used the Metropolis-Hastings Monte Carlo Markov Chain algorithm. The acceptance ratio for each step in the chain was determined by the DCA parameters. Specifically, for a given sequence realization, we calculated the "energy" of the sequence by summing the values within the h and J matrices corresponding to the realized sequence elements. When proposing a random mutation, the acceptance probability was calculated by taking the exponential of the inverse effective temperature (β) multiplied by the energy difference between the old and newly mutated sequences. We used temperature annealing to maintain an overall acceptance probability of 30%.

After training the DCA models on 37 randomly selected sequences from the >3000 generated sequences, we assessed the model's performance by calculating the Receiver Operating Characteristic (ROC) curve for the hardcoded J tensor versus the learned J tensor. The ROC curve, which plots the true positive rate versus the false positive rate, enables us to quantify the model's ability to learn our toy-model hardcoded interactions between sequence indexes and importantly, specific amino acid identities. We used this metric because it helps us understand how well the model discriminates true positives from noise in a highly skewed dataset with many true negatives and few true positives. A result

was only considered a true positive if the model correctly predicted both amino acid identities and sequence indexes. We classified the hardcoded *J* matrix by setting all elements within the $17 \times 17 \times 21 \times 21$ tensor to class zero, unless the value exceeded a "hardcoded threshold," in which case the class was set to one. The threshold value was typically set to zero, except when classifying the randomly generated dataset, where a higher threshold optimized the ROC area under the curve (AUC).

The learned *J* matrices were classified using a different method. First, we calculated the norm of the *J* matrix. Then, if an element of the 17×17 normed *J* matrix was less than a "norm threshold," all elements of the 21×21 matrix corresponding to that element were set to 0. If the element exceeded the threshold, we set all elements in the 21×21 matrix greater than 0 to their given *J* score, and all other values to class 0. We then normalized the classified *J* matrix so that all values fell between 0 and 1, so that the scores represented the probability of being class 1. Using the hardcoded *J* matrix as the true labels and the learned *J* matrix scores and probabilities, we used SciKitLearn's roc_curve function to calculate the ROC statistics. The roc_curve function computes the true positive and false positive rates across multiple probability thresholds, assigning class one to predictions above each threshold (e.g., probabilities greater than 0.1, 0.2, 0.3, etc.). ROC curves were computed for *J* matrices learned from 37 sequences, 3000 sequences (**Figure S19a**), 37 Gaussian sequences, and 3000 Gaussian sequences (**Figure S19b**). Given our ROC curves, we computed the ROC AUC as a metric to score the performance of a given model.

Comparison of **Figure S19a** and **Figure S19b** showed that the DCA model trained on just 37 *in silico* peptides was able to fit the underlying sequence distribution better than the DCA model trained on sequences generated using DCA parameters sampled from a Gaussian. As the state-space of possible sequences within a peptide family is greatly reduced due to the intrinsic requirements of conserved amino acids and correlated regions, it can be hypothesized that the DCA's ability to better fit this data is due to the increased probability that the 37 sampled sequences will have repeat patterns. As one would expect, **Figure S19c**, which plots the ROC AUC versus sequence parameters, showed that an increasing number of correlated pairs and amino acids per correlation decreases the model ability to fit the underlying distribution. However, the model still learned meaningful information, notably more than the gaussian sequences. This phenomenon, shown though the ROC curves, can further be assumed to apply to any sequence space with intrinsic constraints (i.e., real

peptide families) implying a possible generalization of the methods described here despite a small number of datapoints.

Besides quantifying the discrimination power of the DCA model on 37 sequences, we also aimed to assess how well the sequences generated using the learned DCA parameters represented the hardcoded sequence distribution. There are the hardcoded-peptide parameters, and the parameters sampled from a gaussian distribution, called hardcoded-gaussian parameters. The hardcoded-peptide parameters in the Metropolis-Hastings scheme generate 3000 hardcoded-peptide sequences. The hardcoded-gaussian parameters generated the 3000 hardcoded-gaussian sequences. We trained DCA models on the hardcoded-peptide sequences to generate DCA parameters referred to as the learned-3000 parameters. Using a random subset of 37 sequences, we obtained the learned-37 parameters. The hardcoded-gaussian sequences provided the learned-3000 Gaussian parameters and learned-37 Gaussian parameters. From the learned parameters, we sampled sequences again, resulting in the learned-3000 sequences, learned-37 sequences, learned-3000 Gaussian sequences, and learned-37 Gaussian sequences. Each learned sequence set contained over 3000 sequences. Additionally, we generated a set of sequences randomly, without additional logic, as a negative control, referred to as the random sequences.

For each learned sequence set, we calculated the energy of a given sequence in two different ways. For example, with the learned-37 sequence set, we can calculate the energy using the DCA parameters used to generate the sequences (i.e., the learned-37 parameters) or the hardcoded-peptide parameters. By quantifying the energy of the learned-37 sequence set using the hardcoded-peptide parameters, we created an energy distribution, with each of the 3000 sequences in the learned-37 sequence set labelled with an energy value. By comparing the energy distributions of the hardcoded-peptide sequence set and the learned-37 sequence set, we quantified how much information the learned-37 parameter set is missing about the underlying hardcoded-peptide parameters.

To achieve this, we first removed all sequences within the learned-37 sequence set that had an energy score below the 50th percentile when evaluated using the learned-37 parameter set. We then created an energy histogram of the trimmed learned-37 sequence set and the hardcoded-peptide sequence set, evaluated using the hardcoded-peptide parameter set. Using the normalized histograms, we computed the Kullback-Leibler divergence (KL-div) using the SciKitLearn entropy function, adding a small epsilon (10^{-12}) to each bin to ensure

both distributions were well defined across a shared domain. The KL-div quantifies how one probability distribution diverges from another, essentially measuring the pseudo-distance between two distributions. For true distribution P and approximate distribution Q, the KL-div measures the information loss when Q approximates P. A higher KL-div indicates greater information loss, implying that distribution Q is a poorer approximation of P.

This analysis was performed for the learned-3000 sequences, learned-37 sequences, learned-3000 Gaussian sequences, and learned-37 Gaussian sequences across the 72 hardcoded-peptide parameter sets and 3 hardcoded-Gaussian parameter sets. The results of **Figure S20b** demonstrates that the energy distribution of the learned-37 sequences contains more than a magnitude more information than the learned-37 gaussian sequences, seen from the respective average KL-div values of 0.31 ± 0.12 and 1.92 ± 0.41. The amount of learned information is even more apparent in **Figure S20c**, comparing the KL-div values of the mean KDE estimations of the learned-37 sequence and the random sequences, 0.18 and 19.57 respectively. Furthermore, somewhat surprisingly the difference in the average KL-div between the learned-37 sequences and learned-3000 is not significant. All this together displays the apparent effectiveness of the plmDCA model coupled to a Metropolises' algorithm with a Pott's model energy function to learn a significant quantity of information about the underlying sequence distribution of a peptide family, even with a dataset of only 37 sequences. Visually, **Figure S21** showed the similarity of a representative sequence distribution using MSA logos of 3000 sequences and 37 sequences. This displayed how the large decease in possible sequence space from the constraints of belonging to a peptide family increased the probability of repeat patterns occurring from a small subset of the overall distribution of sequences.

Overall structure of huPA in complex with bicyclic peptide UK965 and UK970

The electron density of the non-glycosylated catalytic domain of huPA (Ile16 to Leu250, chymotrypsin numbering) in complex with bicyclic peptides UK965 or UK970 is clearly visible for all residues except for the last six amino acids at the C-terminal end. Like in the huPA-UK18 crystal structure, a single molecule of huPA is present in the asymmetric unit. For both huPA-UK965 and huPA-UK970 complexes, the overall structure of huPA does not show any striking rearrangements of the main backbone if compared to other huPA crystal structures,

belonging to the same or different space groups, that have been determined either in the apo form or in complex with inhibitors (**Figure S7a** and **S8b**).

Overall structure of bicyclic peptides UK965 and UK970 and intra-molecular interactions

The electron density of the bicyclic peptides UK965 and UK970 is well-defined for the residues from Cys2 to Gly17 allowing an unambiguous assignment of group orientations for protein complex present in the asymmetric unit. The electron density of the N-terminal Ala1 of both UK965 and UK970 is not detectable since it is solvent exposed and disordered (**Figure S8**). No classical secondary structure elements are found in the bicyclic peptides (**Figure 3**). The non-covalent intra-molecular interactions present appear to confer structural rigidity to the peptides (**Figure S9** and **Table S8**). The first loop (residues from Cys2 to Cys9) of both UK965 and UK970 forms two consecutives β-turns (Ser3 to Glu6 and Glu6 to Cys9 in UK965 and Ser3 to Val6 and Val6 to Cys9 in UK970). A change of direction occurs at the level of Cys9 followed by the second loop (residues from Cys9 to Cys16) that presents two additional β-turns (Gly11 to Gly14 and Gly14 to Gly17) that links the remaining residues running roughly anti-parallel. Bicyclic peptides UK965 and UK970 present five and four intra-molecular hydrogen bond interactions, respectively (**Figure S9** and **Table S8**). Differently from UK18, all these intra-molecular hydrogen bonds are mediated by main-chain to main-chain contacts. Notably, the number of intra-molecular hydrogen bonds of UK965 and UK970 have better angles of interaction and are therefore more energetically favourable than those of UK18.

Inter-molecular interactions between huPA and bicyclic peptide UK965 and UK970

Bicyclic peptides UK965 and UK970 fit well into the cleft formed by the active site and the surrounding substrate pockets covering a large protein surface of 749 $Å^2$ and 746 $Å^2$, respectively (**Table S4**). Both peptide loops of UK965 and UK970 interact directly with huPA. The second loop of both UK965 and UK970 forms more interactions than the first loop and hence contributes more to the overall binding. The residues of UK965 and UK970 contacting huPA through both main and side chain hydrogen bond interactions are eight for both UK965 (Arg4, Glu6, Val7, Asp8, Arg10, Arg12, Gly13 and Gly17) and UK970 (Arg4, Val6, Val7,

Asp8, Arg10, Arg12, Gly13 and Gly17; **Figure 3, Figure S9** and **Table S6**). In the first peptide loop of UK965 most interactions with huPA are mediated by Asp8 that forms three hydrogen bonds through its side chain (Asp8 OD1 with His57 NE2, Asp8 OD2 with Gly193 N and Asp8 OD2 with Ser195 OG), a water mediated hydrogen bond between its side chain and the main chain oxygen of Ser214 (Asp8 OD2 with Ser214 O) and one hydrogen bond between its main chain nitrogen and the Val41 carbonyl group (Asp8 N with Val41 O; **Figure S9** and **Table S6**). The side chain of Arg4 forms two hydrogen bonds with Arg35 (Arg4 NH1 with Arg35 NH2) and Arg37^{A} (Arg4 NH2 with Arg37^{A} N). The side chain of Glu6 forms a hydrogen bond with Gln192 (Glu6 OE2 with Gln192 NE2) and a water mediated hydrogen bond with Tyr151 (Glu6 OE2 with Tyr151 OH). Furthermore, the main chain oxygen of Glu6 forms a water-mediated hydrogen bonds with main chain oxygen Tyr40 (Glu6 O with Tyr40 O). The main chain oxygen of Val7 forms a hydrogen bond with the side chain of Gln192 (Val7 O to Gln192 NE2). The remaining amino acids Ser3 and Tyr5 of the first peptide loop occupy the S1' pocket of huPA but the atomic distances do not suggest the formation of hydrogen bond interactions. The most important interactions of the second loop of UK965 to huPA are mediated by Arg12 whose basic side chain occupies the primary specificity S1 pocket and interacts with the Asp189 carboxylate at the bottom of the cavity (**Figure S9** and **Table S6**). The side chain of Arg12 forms salt bridges with the side chain of Asp189 (Arg12 NH1 with Asp189 OD1 and Arg12 NH2 with Asp189 OD2). Additionally, the side chain of Arg12 forms two hydrogen bonds with Gly218 (Arg12 NE with Gly218 O and Arg12 NH2 with Gly218 O), one hydrogen bond with Ser190 (Arg12 NH1 with Ser190 OG) and three water mediated hydrogen bonds with Arg217 (Arg12 NH2 with Arg217 O), Leu 222 (Arg12 NH2 with Leu222 N) and Lys224 (Arg12 NH2 with Lys224 O). Furthermore, the main chain nitrogen of Arg12 forms a water mediated hydrogen bond with Ser195 (Arg12 N with Ser195 OG) (**Figure S9** and **Table S6**). Similarly, the side chain of Arg10 in the second peptide loop, forms two salt bridges with the side chain of Asp60^{A} (Arg10 NH2 with Asp60^{A} OD1 and Arg10 NE with Asp60^{A} OD2). Additionally, the main chain oxygen of Arg10 forms a water mediated hydrogen bond with the main chain oxygen of Ser210 (Arg10 O with Ser214 O). The main chain of Gly13 forms three water mediated hydrogen bonds with Lys143 (Gly13 O with Lys143 NZ), Ser146 (Gly13 O with Ser146 O) and Gly216 (Gly13 N with Gly216 O), whereas the main chain oxygen of Gly17 forms two water mediated hydrogen bonds with Gly216 (Gly17 O with Gly216 N and Gly17 O with Gly216 O). The remaining amino acids Gly11, Gly14 e Pro15 of the second peptide loop do not form polar inter-molecular interactions with huPA. The network of hydrogen bonds formed by UK970 with uPA is very

similar to that described above for UK965 (**Figure 3**, **Figure S9** and **Table S6**). The only differences concern Glu6, which has been replaced by a Val in UK970. While Val6 still retains its water mediated interactions with Thr29 (Val6 O with Thr29 OG1) and Tyr40 (Val6 O with Tyr40 O), it loses that with Tyr151 OH. Furthermore, in addition to the three water mediated hydrogen bonds formed with Lys143, Ser146 and Gly216 observed in UK965, the main chain of UK970's Gly13 also forms a water mediated hydrogen bond with the side chain of Gln192 (Gly13 O with Gln192 OE1). Finally, in addition to the numerous interactions described above, the main chain nitrogen of UK970's Arg12 forms a new water mediated hydrogen bond interaction with Ser214 (Arg12 N with Ser214 O), a contact that in UK965 was established by the side chain of Asp8 (**Figure 3**, **Figure S9** and **Table S6**).

Comparison of the binding interactions of UK18, UK965 and UK970 bicyclic peptides with huPA

Comparison of the crystal structure complexes revealed some differences at the level of the first loop (Cys2 – Cys9) while the second loop (Cys9 – Cys15) of all three bicyclic peptides UK18, UK965 and UK970 binds huPA in a very similar fashion (**Figure 3** and **4**, **Figure S9** and **S10**, and **Table S6** and **S7**). The first loop of UK965 and UK970 adopts a completely different conformation from that of UK18. Consequently, the side chain of Arg4 of both UK965 and UK970 establishes a hydrogen bond with the side chain of Arg35 (Arg4 NH1 – Arg35 NH2) and both the side and main chain of Arg37^{A} (Arg4 O – Arg37^{A} NH2 and Arg4 NH2 – Arg37 N), instead absent in UK18. The same aliphatic side chain of Arg4 of both UK965 and UK970 further forms numerous nonpolar contacts with aliphatic side chain of Arg35 and Tyr60^{B} of huPA, otherwise not detectable in UK18. Similarly, the main chain oxygen of Val (UK970) and Glu (UK965) in position 6, forms a water mediated bond with both side chain of Thr29 (Val6 O – H_2O – Thr29 OG1 and Glu6 O – H_2O – Thr29 OG1) and the main chain oxygen of Tyr40 (Val6 O – H_2O – Tyr40 O and Glu6 O – H_2O – Tyr40 O), otherwise absent in UK18. While UK18 presents an interaction between the main chain oxygen of Ala1 and the hydroxyl group of Tyr60B (Ala1 O – Tyr60^{B} OH), this is instead absent in both UK965 and UK970. While the density of Ala1 was well defined in UK18, it was not visible in both UK965 and UK970. (**Figure S9** and **S10**, and **Table S6** and **S7**). Despite the significant conformational change, some similarities are found in the first loop of all three bicyclic peptide inhibitors. For instance, the hydrophobic side chain of the conserved Val7 continues to fit well in the hydrophobic pocket of huPA defined by Val41,

Tyr40, Leu81 and Tyr151. Differently from UK18, the side chain of Val7 of UK965 and UK970 engages in a non-polar interaction with main chain of Gly193. Moreover, the conserved negatively charged amino acid Asp8 continues to establish multiple hydrogen-bonding and non-polar interactions with both side and main chains of surrounding Val41, His57, Gly193 and Ser195 of huPA (**Figure S9** and **S10**, and **Table S6** and **S7**). A major difference between UK970 and UK965 is the presence of a Phe in placed on a Tyr residue in position 5. This causes the water molecule, present in UK965 and coordinated by the side chain hydroxyl group of Tyr5 (UK965), the main chain oxygen of Arg37^{A} and the side chain hydroxyl group of Ser37^{D} of huPA, to be absent in UK970 bearing instead a Phe in the same position (**Figure S9** and **S10**, and **Table S6** and **S7**). Binding mode of the second loop of all three bicyclic peptides to huPA is very similar. The conserved Arg12 residue occupies the S1 subsite of huPA and forms multiple direct and water-mediated hydrogen bonds with the side chain of Asp189, both side and main chain of Ser190, and the main chain oxygen of Ser195, Ser214, Arg217, Gly218 and Lys224 (**Figure S9** and **S10**, and **Table S6** and **S7**). Similarly, the highly conserved Arg10 residue forms multiple direct and water-mediated hydrogen-bonding interactions with the side chain of Asp60^{A} of huPA (Arg10 NH2 – Asp60^{A} OD1 and Arg10 NE – Asp60^{A} OD2) and Ser214 (Arg10 O – H_2O – Ser214 O). Despite the high similarities, some differences exist at the level of Gly13 and Gly17 residues. For instance, the main chain oxygen and nitrogen of Gly13 of both UK965 and UK970, forms water-mediate hydrogen bonds with the side chain of Lys143, and the main chain oxygen of both Ser146 and Gly216, otherwise absent in UK18 (**Figure S9** and **S10**, and **Table S6** and **S7**). Similarly, the main chain oxygen of Gly17 of both UK965 and UK970, whose electron density was not detectable in UK18, establishes water-mediated hydrogen bonds with main chain oxygen and nitrogen of Gly216 (Gly17 O- H_2O – Gly216 O and Gly17 O- H_2O – Gly216 N). The same Gly17 of UK965 and UK970 engages in non-polar interactions with surrounding Thr97^{A}, Leu97^{B} and His99, otherwise absent in UK18.

Molecular basis for the target specificity of UK965 and UK970 bicyclic peptides to huPA

The high target specificity of UK965 and UK970 bicyclic peptides to huPA can be attributed to their ability of engaging in inter-molecular interactions with residues of huPA that are different in homologous serine proteases and/or steric factors that hinder the three bicyclic peptide inhibitors from binding to the homologous proteases. The substrate binding cleft of

huPA differs significantly from the homologous serine proteases in the S1-prime subsite (or S1'). In this subsite, the residues that are in close contact to UK965 and UK970 are Arg35, His37, Arg37^{A}, Gly37^{B}, Thr39, Asp60^{A} and Tyr60^{B} residues (**Figure 3** and **4**, **Figure S9** and **S10**, and **Table S6** and **S7**). The most important interactions of this subsite with UK965 and UK970 are mediated by *i*) the side chain of Arg35 that forms one hydrogen bond and multiple nonpolar contacts with the aliphatic side chain of Arg4, *ii*) the side chain of His37 that establishes nonpolar contacts with side chain of either Tyr5 (UK965) or Phe5 (UK970), *iii*) the main chain of Arg37^{A} that forms one hydrogen bond with side chain of Arg4, *iv*) the Asp60^{A} side chain that forms two hydrogen bonds with Arg10 and *v*) the Tyr60^{B} side chain that engages in multiple nonpolar contacts with the aliphatic side chain of Arg4 (**Figure S9** and **S10**, and **Table S6** and **S7**). Since the amino acids in position 60A and 60B are different in all the tested trypsin-like serine proteases, these proteases cannot form the same polar and nonpolar interactions with UK965 and UK970 bicyclic peptides. Another subsite in which the amino acids of different trypsin-like serine proteases vary significantly is the S1 sub-pocket also termed S1β. In this site, the side chains of Ser190 and Gln192 as well as the main chain of Gly193 make hydrogen bond interactions with Val7, Asp8, Arg12 and Gly13 of both UK965 and UK970 (**Figure S9** and **S10**, and **Table S6** and **S7**). While about half of the trypsin-like serine proteases have also a serine residue in position 190, the others contain an alanine and thus can hence not form a hydrogen bond to Arg12 with their side chain. In position 192, many homologous proteases have a lysine or glutamate residue that cannot form the same hydrogen bond as the Gln192 to Val7 present in UK965 and UK970. A number of amino acids of huPA interacting with UK965 and UK970 are similar or identical in many members of the trypsin-like serine protease family and interactions of UK965 and UK970 with those are likely to be less relevant for the specificity of the inhibitor. These include the amino acids Val41, Asp189 and Gly218 that are rather conserved within the family of trypsin-like serine proteases and the invariant amino acids His57 and Ser195 of the catalytic triad.

Supplementary experimental procedures

Chemical synthesis of peptides

Peptides with a free amine at the N-terminus and an amide at the C-terminus were chemically synthetized by standard Fmoc (9-fluorenylmethoxycarbonyl) solid-phase peptide synthesis (SPPS). Fmoc-protected amino acids, (Benzotriazol-1-yloxy)tripyrrolidinophosphonium hexafluorophosphate (PyBOP), acetic anhydride, anisole, dichloromethane (DCM), N,N-dimethylformamide (DMF) and Rink Amide MBHA resin (100 - 200 mesh, loading 0.4 - 0.9 mmol/g resin, 0.01 mmol scale) were purchased from Novabiochem (Darmstadt, Germany). Acetonitrile (ACN), formic acid, N-methylmorpholine (NMM), octanedithiol (ODT), 1,3,5-tris(bromomethyl)benzene (TBMB), piperidine, diethyl ether, trifluoroacetic acid (TFA) and thioanisole were purchased from Sigma-Aldrich (Darmstadt, Germany). N-methylpirrolidone (NMP) was purchased from VWR (Pennsylvania, USA). All chemicals were used as received without further purification. Peptides were chemically synthesized using a ResPepSLi automated peptide synthesiser (Intavis Bioanalytical Instruments, Köln, Germany) as previously described.[16] Briefly, Fmoc groups were removed using a 20% v/v solution of piperidine in DMF (180 µL × 2). Amino acid coupling was carried out twice for each Fmoc-amino acid (7.5 eq., 0.5 M solution in DMF) using the PyBOP/NMM coupling system (5.5 eq. 0.4 M / 9 eq. 4 M in DMF). Fmoc groups were removed using a 20% v/v solution of piperidine in DMF. Final acetylation capping was performed using a 5% v/v solution of acetic anhydride in DMF. DCM washes (0.3 mL × 5) were performed at the end of the synthetic process. NMP was used as cosolvent in the peptide synthesis. 4 M NMM solution in DMF was added as weak base for coupling reaction. The final peptides were deprotected (side-chain protected groups) and cleaved from the resin using a TFA/thioanisole/H_2O/anisole/ODT mixture (90/2.5/2.5/2.5/2.5% v/v) for 3 h at room temperature. The resin was removed by filtration under vacuum and the peptides were precipitated with cold diethyl ether (50 mL). The precipitated peptides were resuspended in diethyl ether (30 mL × 2) and centrifuged (3 times). Finally, the peptides were dissolved in H_2O:ACN (1:1), freeze-dried and lyophilized on a LIO-5PDGT (5Pascal, Milan, Italy).

Chemical cyclisation of peptides

Bicyclic peptides modified with TBMB were obtained by reacting crude peptides (1 mM) in 70% v/v 20 mM NH_4HCO_3, pH 8.0 and 30% v/v ACN with TBMB (1.5 mM) for 1 hr at 30 °C. The reaction products were purified by preparative reversed-phase high performance liquid chromatography (RP-HPLC) using a C18 SymmetryPrep functionalized silica column (7 μm, 19 mm × 150 mm, Waters, Millford, MA, USA) connected to a Waters Delta Prep LC 4000 System equipped with a Waters 2489 dual λ absorbance detector, a Waters 600 pump and a PrepLC Controller (Waters, Millford, MA, USA). A flow rate of 20 mL/min and a linear gradient (10% to 50% in 35 min) with a mobile phase composed of eluant A (99.9% v/v H_2O, 0.1% v/v TFA) and eluant B (99.9% v/v ACN and 0.1% v/v TFA) was applied. The purified peptides were freeze-dried. The purity and molecular mass of the peptides was assessed by LC-ESI as described below. Concentrations of peptides were determined using a BioPhotometer D30 UV spectrophotometer (Eppendorf, Hamburg, Germany).

Mass spectrometry analysis of bicyclic peptides

The molecular mass of each bicyclic peptide was determined by electrospray ionisation mass spectrometry (ESI–MS) performed on a single quadrupole liquid chromatograph InfinityLab LC/MSD mass spectrometer coupled to a 1260 Infinity II LC system (Agilent Technologies, Santa Clara, CA, USA). The system operated with the standard ESI source and in the positive ionisation mode. Peptides were run at a flow rate of 1 mL/min with a linear gradient of solvent B over 15 min (solvent A: 99.9% v/v H_2O and 0.1% v/v formic acid; solvent B: 99.9% v/v ACN and 0.1% v/v formic acid). The reversed-phase HPLC column was a Nucleosil 100-5 C18 (5 μm, 125 mm × 4 mm; Macherey-Nagel, Dueren, Germany). Data were acquired, processed and analyzed using the Agilent OpenLAB CDS (Agilent Technologies, Santa Clara, CA, USA) and MestReNova (Mestrelab Research, Santiago de Compostela, Spain).

Determination of inhibitory activity of bicyclic peptides

The inhibitory activity of bicyclic peptides was assessed by monitoring the residual activity of huPA in the presence of a fluorogenic substrate and different concentrations of inhibitor

bicyclic peptides. The activity assay was performed by incubating 15 nM huPA with 50 µM fluorogenic substrate Z-Gly-Gly-Arg-AMC (50 µM; Bachem, Bubendorf, Switzerland) and two-fold peptide dilutions (0, 0.12, 0.24, 0.48, 0.97, 1.95, 3.9, 7.81, 15.6, 31.2, 62.5, 125, 250, 500, 1000 and 2000 nM). All reagents were diluted in 10 mM Tris-Cl, pH 7.4, 150 mM NaCl, 10 mM $MgCl_2$, 1mM $CaCl_2$, 0.1% w/v BSA, 0.01% v/v Triton-X100 and 5% v/v DMSO. The measurements were performed on a Tecan microplate reader (Tecan infinite 200 pro, Tecan Trading AG, Switzerland) using black microfluor 96-well plate Nunc MicroWell, (Thermo Fisher Scientific, Dreieich, Germany) The enzymatic reactions were performed at 25 °C for 30 min, under shacking with an excitation wavelength of 355 nm and an emission recording at 460 nm. The initial velocities were monitored as changes in fluorescence intensity. The sigmoidal curves were fitted to the data using the following non-linear regression equation for the inhibitory dose-response curves with variable slope (2):

$$y = \frac{100}{\left(1 + \left(\frac{IC_{50}}{x}\right)^{p}\right)} \tag{2}$$

where *x* is the peptide concentration, y is the residual percentage of protease activity and *p* is the hill slope. Half maximum inhibitory concentration (IC_{50}) values were derived from the fitted curves from GraphPad Prism 8 8.0.0. software (GraphPad software, Inc., San Diego, California). The final K_i was subsequently determined using the Cheng-Prusoff equation (3):

$$K_i = \frac{IC_{50}}{1 + \frac{[S]_0}{K_m}} \tag{3}$$

where K_m (115 µM) is the Michaelis constant for the hydrolysis of Z-Gly-Gly-Arg-AMC catalysed by huPA which has been determined by standard Michaelis-Menten equation. Values were determined using either OriginPro 8G software (OriginLab Corporation, Northamppton, MA, USA) or GraphPad Prism 8.0.0. software (GraphPad software, Inc., San Diego, California).

Specificity determination of bicyclic peptide

Residual activities were measured in 150 µL volume of buffer containing 10 mM Tris-Cl, pH 7.4, 150 mM NaCl, 10 mM $MgCl_2$, 1mM $CaCl_2$, 0.1% w/v BSA, 0.01% v/v Triton-X100 and

5% v/v DMSO. Final concentrations of serine proteases were the following: human uPA (UPA-LMW, Molecular Innovations, Novi, MI, USA) 1.5 nM, mouse uPA (MUPA-LMW, Molecular Innovations, Novi, MI, USA) 12 nM, human tPA (HTPATC; Molecular Innovations, Novi, MI, USA) 7.5 nM, mouse tPA (MTPA, Molecular Innovations, Novi, MI, USA) 6 nM, human trypsin (HTRYP, Molecular Innovations, Novi, MI, USA) 0.05 nM, human plasmin (HPLM, Molecular Innovations, Novi, MI, USA) 1.5 nM, human plasma kallikrein (IHPKA, Innovative Research, Novi, MI, USA) 0.5 nM, human thrombin (IHT, Innovative Research, Novi, MI, USA) 10 nM, and human factor XIIa (IHFXIIa, Innovative Research, Novi, MI, USA) 6 nM. Two-fold dilutions of UK970 bicyclic peptide inhibitor were prepared ranging from 1 mM to 125 nM for all the proteases. For human uPA an extra two-fold UK970 inhibitor dilution experiment was performed by using inhibitor concentrations ranging from 2000 nM to 0.12 nM. For the determination of the K_i inhibitory constants, the following fluorogenic substrates were used at final concentration of 50 µM: Z-Gly-Gly-Arg-AMC (for human uPA, murine uPA, human tPA, murine tPA, human trypsin, human thrombin and human factor XIIa; Bachem, Bubendorf, Switzerland), Z-Phe-Arg-AMC (for human plasma kallikrein; Bachem, Bubendorf, Switzerland) and H-D-Val-Leu-Lys-AMC (for human plasmin; Bachem, Bubendorf, Switzerland). The initial velocities were monitored as changes in fluorescence intensity during 30 min on a Tecan microplate reader (Tecan infinite 200 pro, Tecan Trading AG, Switzerland) using black microfluor 96-well plate Nunc MicroWell (Thermo Fisher Scientific, Dreieich, Germany). The enzymatic reactions were performed at 25 °C under shacking with an excitation wavelength of 355 nm and an emission recording at 460 nm. Apparent equilibrium constants K_i^{apps} values were determined by non-linear regression analyses of V_i/V_0 versus $[I]_0$ using equation (2). The final K_is were subsequently determined by correcting for the competitive effect of the substrate $[S]_0$ using equation (3). The kinetic constants K_ms for the hydrolysis of fluorogenic substrate, catalysed by each protease, were determined by standard Michaelis-Menten equation. Values were determined using either OriginPro 8G software (OriginLab Corporation, Northamppton, MA, USA) or GraphPad Prism 8.0.0. software (GraphPad software, Inc., San Diego, California).

Recombinant production of the catalytic domain of human urokinase-type plasminogen activator

The low molecular weight (LMW) human urokinase-type plasminogen activator (huPA) comprising the truncated 23 amino acid peptide fragment of chain A (Lys136-Lys158 in

huPA numbering) and the catalytic domain (also termed chain B; Ile159-Leu411 in huPA numbering or Ile16-Leu250 in chymotrypsin numbering), mutated in two positions to eliminate both the surface-exposed free cysteine residue (Cys122Ala) and the glycosylation site (Asn145Gln), was expressed by transient transfection of suspension-adapted human embryonic kidney FreeStyle 293-F cells (HEK-293-F) as previously described.[17] Briefly, 1 mg of pSecTagA-LMW-huPA-C122A-N145Q plasmid encoding LMW huPA-C122A-N145Q protein was premixed to linear polyethylenimine (PEI, PEI, Polysciences, Heppenheim, Germany) and Opti-MEM (Thermo Fisher Scientific, Dreieich, Germany) and used to transfect 1 L of high cell density (1 x 10^6 cells/ml) HEK-293-F cells growing in serum-free FreeStyle™ 293 Expression Medium (Thermo Fisher Scientific, Dreieich, Germany) in an orbitally shaken one-litre flask at 180 rpm in a Forma Steri-Cycle 370 CO_2 incubator (Thermo Fisher Scientific, Dreieich, Germany) at 37 °C in the presence of 5% CO_2).[18,19] At the end of the 7-day phase production, cells were harvested by centrifugation at 5500 rpm for 20 min at 4 °C on an Avanti J-25 centrifuge (Backman Coulter, Indianapolis, USA). Any additional cell debris was removed from the medium by filtration through 0.45 µm low protein binding membranes (Prat-dumas, Bourg, France).

Purification of the recombinant catalytic domain of human urokinase-type plasminogen activator

The recombinant LMW huPA-C122A-N145Q protein was purified as previously described.[17] Briefly, the protein was concentrated by using 10000 MWCO Amicon Ultra ultrafiltration tube (Merck Novagen, Nottingham, UK) at 3000 g and 4 °C on a 5810R centrifuge (Eppendorf, Hamburg, Germany) and diluted five-times with Buffer A (50 mM sodium phosphate pH 6.2). The protein was captured on 10 mL strong cation exchange SP sepharose fast flow resin (Cytiva, Freiburg, Germany) packed on a XK-16 gravity column (Cytiva, Freiburg, Germany) pre-equilibrated with Buffer B (25 mM sodium phosphate pH 6.4). The diluted medium was passed through the pre-equilibrated resin at 4 °C. After extensive washing with Buffer B (25 mM sodium phosphate pH 6.4), the protein was eluted with Buffer C (25 mM sodium phosphate, 500 mM NaCl, pH 6.4). The protein containing fractions were pooled, concentrated by using 10000 MWCO Amicon Ultra ultrafiltration tube (Merck Novagen, Nottingham, UK) at 3000 g and 4 °C on an 5810R centrifuge (Eppendorf) and at 4 °C, diluted ten-times with Buffer A (50 mM sodium phosphate pH 6.2), and subjected to second cation exchange HiScreen SP HP pre-packed chromatography column (Cytiva, Freiburg,

Germany) connected to an AKTA purifier system (Cytiva, Freiburg, Germany). The diluted protein was passed through the resin pre-equilibrated with Buffer B at a flow rate of 1 mL/min at 4 °C. After extensive washing with Buffer B, the protein was eluted with Buffer C by applying a linear NaCl gradient (0 – 500 mM). The eluted protein showed a single band in SDS-PAGE, with an apparent molecular mass of about 32 kDa. Afterwards the recombinant LMW huPA-C122A-N145Q was converted into its active two-chains form by plasmin cleavage, whereby the first sixteen N-terminal amino acid residue of the A-chain were, due to the elimination of the A-B chain-connecting disulfide bridge by the Cys122Ala exchange, separated from the B-chain. To a solution of 150 µM LMW-huPA-C122A-N145Q in Buffer D (50 mM HEPES, 150 mM NaCl, pH 8.0), 200 nM human plasmin (HPLM, Haematologic Technologies, Essex, VT, USA) was added (ratio 500:1). After incubation for 4 hrs at room-temperature, the cleaved activated protein was further purified by size exclusion chromatography using a HiLoad 26/60 Superdex 200 prep-grade column (Cytiva, Freiburg, Germany) and Buffer E (50 mM HEPES, 100 mM NaCl, pH 7.0) on an AKTA purifier system (Cytiva, Freiburg, Germany). The protein was eluted as a monomer giving a single band in SDS-PAGE confirming the complete cleavage, with a molecular mass of about 28 kDa under reducing condition. The pure and activated LMW huPA-C122A-N145Q in Buffer E was then concentrated to 10 mg/mL (347 µM) for crystallisation by using 5000 MWCO PES Vivaspin-20 ultrafiltration tube (Sartorius-Stedim Biotech GmbH, Göttingen, Germany) at 3000 g and 4 °C on 5810R centrifuge (Eppendorf, Hamburg, Germany). The activity of recombinant LMW huPA-C122A-N145Q before and after plasmin activation was assessed by incubating the protein with the fluorogenic substrate Z-Gly-Gly-Arg-AMC (50 µM; Bachem, Bubendorf, Switzerland) in 10 mM Tris-Cl, pH 7.4, 150 mM NaCl, 10 mM $MgCl_2$, 1mM $CaCl_2$, 0.1% w/v BSA, 0.01% v/v Triton-X100 and 5% v/v DMSO. The measurements were performed on a Tecan microplate reader (Tecan infinite 200 pro, Tecan Trading AG, Switzerland) using black microfluor 96-well plate Nunc MicroWell, (Thermo Fisher Scientific, Dreieich, Germany). The enzymatic reactions were performed at 25 °C for 30 min, under shacking with an excitation wavelength of 355 nm and an emission recording at 460 nm. No inhibitors were added to the buffers.

Crystallization of recombinant huPA in complex with bicyclic peptides

Crystallization trials of huPA in complex with bicyclic peptides UK965 and UK970 were carried out at 293 K using the sitting-drop vapor-diffusion method and the Oryx 8

crystallization robot (Douglas Instruments Ltd, Berkshire, UK). Crystals of huPA in complex with UK965 were obtained by mixing 1 µL of huPA protein (10 mg/mL, 347 µM) in 50 mM HEPES, 100 mM NaCl, pH 7.0, 0.3 µL of UK965 bicyclic peptide (6 mM) in 50 mM citric acid pH 4.3, 7% w/v PEG400, 2 M $(NH_4)_2SO_4$, and 1 µL of precipitant solution and allowed to equilibrate against 200 µL of reservoir solution 50 mM citric acid pH 4.3; 5% w/v PEG400; 1.8 M $(NH_4)_2SO_4$. Best crystals were obtained within 7 days. Crystals of huPA in complex with UK970 were instead obtained by initially growing crystals in a solution containing the lower affinity bicyclic peptide UK18, which was later replaced by the higher affinity bicyclic peptide UK970 inhibitor. Briefly, crystals of huPA in complex with UK18 were obtained by mixing 1 µL of huPA protein (10 mg/mL, 347 µM) in 50 mM HEPES, 100 mM NaCl, pH 7.0, 0.3 µL of UK18 bicyclic peptide (6 mM) in 50 mM citric acid pH 4.3, 7% w/v PEG400, 2 M $(NH_4)_2SO_4$, and 1 µL of precipitant solution and allowed to equilibrate against 200 µL of reservoir solution 50 mM citric acid pH 4.3; 7% w/v PEG400; 1.8 M $(NH_4)_2SO_4$. After 9 days, the best uPA-UK18 crystals were taken and soaked in a suspension of UK970 bicyclic peptide (6 mM) in 50 mM citric acid pH 4.3 7% PEG400, 2 M $(NH_4)_2SO_4$. For X-ray data collection, crystals were soaked in a solution of 20% w/v ethylene glycol, mounted on LithoLoops (Molecular Dimensions Ltd, Suffolk, UK) and flash-cooled in liquid nitrogen.

X-ray data collection and processing

X-ray diffraction data of human uPA-UK965 and uPA-UK970 complexes were collected at beamline ID23-2 and ID23EH2 of the European Synchrotron Radiation Facility (ESRF, Grenoble, France), respectively. The best crystals of huPA in complex with UK965 diffracted to 1.64 Å maximum resolution. Crystals belong to the *H*3 space group, with unit cell parameters: a = 120.9 Å, b = 120.9 Å, c = 42.7 Å, α = 90°, β = 90°, and γ = 120°. The asymmetric unit contains 1 molecule and a solvent content of 44% of the crystal volume. The best crystals of huPA in complex with UK970 diffracted to 1.80 Å maximum resolution. Crystals belong to the *H*3 space group, with unit cell parameters: a = 121.3 Å, b = 121.3 Å, c = 42.8 Å, α = 90°, β = 90°, and γ = 120°. The asymmetric unit contains 1 molecule and a solvent content of 44% of the crystal volume. Frames were indexed and integrated with software XIA2, merged and scaled with software AIMLESS (CCP4i2 crystallographic package).[20]

Structure determination and model refinement

The structures were solved by molecular replacement with software PHASER[21] using as a template the model 3QN7. Refinement was carried on using REFMAC[22] and PHENIX.[23] Rebuilding and fitting of the bicyclic peptides and precipitant agents (ethylene glycol, EDO; polyethylene glycol 400, PEG400; ammonium sulphate; citrate) was performed manually with graphic software COOT.[24] Geometrical parameters of the model were validated using software included in the CCP4i2 crystallographic package.[20] Since the first cycles of refinement, the electron density corresponding to the bound bicyclic peptides and/or precipitant/additive molecules was clearly visible in the electron density map. The final model of huPA-UK965 complex contains 2027 protein atoms, 130 bicyclic peptide atoms, 125 water molecules, and 24 atoms of other molecules. The final crystallographic R factor is 0.206 (R_{free} 0.243). The final model of huPA-UK970 complex contains 1951 protein atoms, 123 bicyclic peptide atoms, 119 water molecules, and 52 atoms of other molecules. The final crystallographic R factor is 0.210 (R_{free} 0.238). Geometrical parameters of the two models are as expected or better for this resolution. Buried surface calculations were performed using program PISA.[25] Intra-molecular and inter-molecular hydrogen bond interactions were analysed by PDBsum,[26] LIGPLOT+,[27] and PyMOL[28] software. All figures were made with PyMOL.[28] The Protein Data Bank (PDB) identification code for the uPA-UK965 complex is 7ZRR and that of the uPA-UK970 complex is 7ZRT.

Supplementary tables

Data and Scripts	Description
X_data.csv	Input data file containing the family *X* of bicyclic peptide inhibitors to be investigated.
plmDCA_script.m	Main MATLAB script calling all the necessary MATLAB scripts listed below.
mexAll.m	MATLAB script to compile the external C programs needed for DCA analysis.
plmDCA_asymmetric.m	Main MATLAB script to perform DCA analysis.
givernaJ_and_h_plmDCA_asymmetric.m	MATLAB script to create the *h* vector and *J* matrix associated to the DCA analysis.
export_h.m	MATLAB script to export the *h* vector in a text file.
export_J.m	MATLAB script to export the *J* vector in a text file.
FASTA_file_generation_from_csv.ipynb	Python script (in Jupyter Notebook format) that translates the input file of bicyclic peptides in FASTA format.
Monte_Carlo_simulation.ipynb	Python script (in Jupyter Notebook format) that generates new bicyclic peptides using Monte Carlo (MC) simulation.
Random_Forest_regression_analysis.ipynb	Python script (in Jupyter Notebook format) that performs Random Forest Regression (RFR) on the bicyclic peptides generated by plmDCA and MC simulation.
Jij_matrices_visualization.ipynb	Python script (in Jupyter Notebook format) that allows for visualizing the *J* matrix generated by plmDCA and its submatrices.

Supplementary table 1. Input data, MATLAB and Python scripts used for the *in silico* molecular evolutionary approach. The data and scripts names (on the left) and their description (on the right) are reported for each step. The source code and data used to produce the results and analyses presented in this manuscript are available from Open Science Framework (OSF) data repository: https://osf.io/gn6bz/?view_only=20805b7801ba4610a370080e3835fb3c.

protease	substrate	K_m ± S.E. (μM)	K_i ± S.E. (μM)
human uPA (huPA)	Z-Gly-Gly-Arg-AMC	112 ± 19	0.004 ± 0.001
murine uPA (muPA)	Z-Gly-Gly-Arg-AMC	48 ± 12	> 1000
human tPA (htPA)	Z-Gly-Gly-Arg-AMC	94 ± 3	> 1000
murine tPA (mtPA)	Z-Gly-Gly-Arg-AMC	137 ± 34	> 1000
human plasmin	H-D-Val-Leu-Lys-AMC	$(6.1 \pm 1.8) \times 10^2$	> 1000
human trypsin	Z-Gly-Gly-Arg-AMC	54 ± 20	> 1000
human plasma kallikrein	Z-Phe-Arg-AMC	41 ± 14	> 1000
human thrombin	Z-Gly-Gly-Arg-AMC	199 ± 67	> 1000
human factor XIIa	Z-Gly-Gly-Arg-AMC	120 ± 3	> 1000

Supplementary Table 2. **Inhibitory activity and specificity of bicyclic peptide UK970**. The inhibitory activity (K_i) values of bicyclic peptide UK970 towards huPA and various other trypsin-like serine proteases was determined at 25 °C, at physiological pH (7.4) and using the indicated fluorogenic substrates at a concentration of 50 μM. The K_m values of each protease were determined by standard Michaelis-Menten kinetics and used in the calculation of the reported K_i values. S.E., standard error.

**Data collection**	**huPA-UK965**	**huPA-UK970**
Wavelength (Å)	0.873	0.873
Space group	*H*3	*H*3
Cell parameters		
a, *b*, *c* (Å); α, β, γ (°)	120.9, 120.9, 42.7; 90, 90, 120	121.3, 121.3, 42.8; 90, 90, 120
Resolution (Å)	39.58 – 1.64 (1.67 – 1.64)	39.68 – 1.80 (1.84 – 1.80)
Observations	54617 (2761)	173005 (6569)
Unique	28238 (1430)	21768 (1306)
Multiplicity	1.9 (1.9)	7.9 (5.0)
R_{merge}	0.059 (0.711)	0.136 (0.77)
<*I* / σ(*I*)>	8.5 (1.1)	15.4 (2.1)
$CC_{1/2}$	0.997 (0.317)	0.997 (0.588)
Completeness (%)	99.3 (0.317)	99.9 (99.3)
Wilson B-factor	19.0	19.8
**Refinement**		
No. reflections (Used for R_{free} calculation)	28204 (1368)	21767 (1082)
R_{work} / R_{free}	0.206 (0.243)	0.210 (0.238)
Number non-hydrogen atoms	2306	2245

protein (chain A)	2027	1951
bicyclic peptide (peptide, ZBR)	130	123
ions (SO_4)	0	10
other ligands (EDO, 1PE, PEG)	24	42
H_2O	125	119
**Geometry**		
RMSD values		
bond lengths (Å)	0.008	0.011
bond angles (°)	1.596	1.655
Ramachandran plot (%)		
most favoured	97	97
additionally allowed	3	3
outliers	0	0
Average B-factor	24	23

Supplementary Table 3. **Statistics on X-ray structure data collection and refinement**. Data collection and refinement statistics of huPA in complex with bicyclic peptides UK965 and UK970. A single crystal was used to collect all diffraction data. Highest-resolution shell statistics are shown within brackets.

Protein and bicyclic peptide complex	huPA-UK18	huPA-UK965	huPA-UK970
PDB	3QN7	7ZRR	7ZRT
Length of peptides (amino acid residues)	17	17	17
Buried surface area on protein (Å^2)	730	749	746
Buried surface area on bicyclic peptide (Å^2)	940	949	954

Supplementary Table 4. **Buried surface in protein and bicyclic peptide complexes**. Buried surfaces were calculated using the software PDBsum with a probe of 1.4 Å radius and are reported here for the protein huPA and for the bicyclic peptides UK18, UK965 and UK970. The designation "buried" implies that the residues are at least partially inaccessible to bulk solvent because of the proximity of the interface surfaces of the protein and the bicyclic peptides. The difference of the sum in buried surface area of interaction (Δ, Å^2) between huPA-UK965 complex and huPA-UK18 complex (28 Å^2), huPA-UK970 and huPA-UK18 complex (30 Å^2), and huPA-UK970 and huPA-UK965 complex (2 Å^2) have been determined by using the following equation (4):

$$\Delta\left(\text{Å}^2\right) = [(P^X + p^X) - (P^Y + p^Y)]$$

where P^X is the buried surface area on protein in complex X, p^X is the buried surface area on bicyclic peptide in the same complex X, P^Y is the buried surface area on protein in complex Y and p^Y is the buried surface area on bicyclic peptide in the same complex Y.

Protein and bicyclic peptide complex		number of residues at the interface	number of salt bridges	number of hydrogen bonds	number of hydrogen bonds water mediated	number of no-bonded contacts
huPA-UK18	huPA	27	4	10	8	112
	UK18	11				
huPA-UK965	huPA	28	4	9	14	134
	UK965	12				
huPA-UK970	huPA	28	4	9	13	143
	UK970	11				

Supplementary Table 5. **Inter-molecular interactions between huPA and bicyclic peptides**. Number of residues of huPA and bicyclic peptides UK18, UK965 and UK970 forming polar inter-molecular interactions. Total number of inter-molecular salt bridges, hydrogen bonds (direct or waterer mediated) and non-polar interactions have been defined using the software LIGPLOT+.[27]

huPA-UK18 complex	huPA-UK965 complex	huPA-UK970 complex
	Thr29 OG1 – H_2O – Glu6 O (2.5 Å and 3.1 Å) [HB]	Thr29 OG1 – H_2O – Val6 O (2.7 Å and 3.0 Å) [HB]
	Arg35 NH2 – Arg4 NH1 (3.3 Å) [HB]	Arg35 NH2 – Arg4 NH1 (2.9 Å) [HB]
	Arg37^{A} N - Arg4 NH2 (3.1 Å) [HB]	Arg37^{A} N - Arg4 NH2 (3.0 Å) [HB]
		Arg37^{A} NH_2 – Arg4 O (3.22 Å) [HB]
	Tyr40 O – H_2O – Glu6 O (2.7 Å and 3.1 Å) [HB]	Tyr40 O – H_2O – Val6 O (2.7 Å and 3.0 Å) [HB]
Val41 O – Asp8 N (3.1 Å) [HB]	Val41 O – Asp8 N (3.2 Å) [HB]	Val41 O – Asp8 N (3.1 Å) [HB]
His57 NE2 – Asp8 OD1 (2.9 Å) [HB]	His57 NE2 – Asp8 OD1 (2.9 Å) [HB]	His57 NE2 – Asp8 OD1 (2.9 Å) [HB]
Asp60^{A} OD1 – Arg10 NH2 (3.3 Å) [SB]	Asp60^{A} OD1 – Arg10 NH2 (3.1 Å) [SB]	Asp60^{A} OD1 – Arg10 NH2 (3.3 Å) [SB]
Asp60^{A} OD2 – Arg10 NE (2.9 Å) [SB]	Asp60^{A} OD2 – Arg10 NE (2.9 Å) [SB]	Asp60^{A} OD2 – Arg10 NE (2.8 Å) [SB]
Tyr60^{B} OH – Ala1 O (3.2 Å) [HB]		
His99 NE2 – H_2O – Arg10 NH1 (3.1 Å and 3.1 Å) [HB]		
His99 NE2 – H_2O – Cys16 O (3.1 Å and 2.8 Å) [HB]		
	Lys143 NZ – H_2O – Gly13 O (2.6 Å and 3.3 Å) [HB]	Lys143 NZ – H_2O – Gly13 O (2.7 Å and 3.1 Å) [HB]

	Ser146 O – H_2O – Gly13 O (3.3 Å and 3.3 Å) [HB]	Ser146 O – H_2O – Gly13 O (3.3 Å and 3.1 Å) [HB]
	Tyr151 OH – H_2O – Glu6 OE2 (2.6 Å and 2.7 Å) [HB]	
Asp189 OD1 – Arg12 NH1 (2.8 Å) [SB]	Asp189 OD1 – Arg12 NH1 (2.7 Å) [SB]	Asp189 OD1 – Arg12 NH1 (2.8 Å) [SB]
Asp189 OD2 – Arg12 NH2 (2.7 Å) [SB]	Asp189 OD2 – Arg12 NH2 (2.8 Å) [SB]	Asp189 OD2 – Arg12 NH2 (2.8 Å) [SB]
Ser190 OG – Arg12 NH1 (2.8 Å) [HB]	Ser190 OG – Arg12 NH1 (2.8 Å) [HB]	Ser190 OG – Arg12 NH1 (2.7 Å) [HB]
Gln192 N – Arg12 O (3.1 Å) [HB]	Gln192 NE2 – Glu6 OE2 (2.8 Å) [HB]	Gln192 OE1 – H_2O – Gly13 O (2.8 Å and 3.1 Å) [HB]
Gln192 NE2 – Val7 O (2.8 Å) [HB]	Gln192 NE2 – Val7 O (2.9 Å) [HB]	Gln192 NE2 – Val7 O (3.1 Å) [HB]
Gly193 N – Asp8 OD2 (3.1 Å) [HB]	Gly193 N – Asp8 OD2 (2.7 Å) [HB]	Gly193 N – Asp8 OD2 (2.8 Å) [HB]
Ser195 OG – Asp8 OD2 (2.8 Å) [HB]	Ser195 OG – Asp8 OD2 (2.8 Å) [HB]	Ser195 OG – Asp8 OD2 (2.6 Å) [HB]
Ser195 OG – H_2O – Arg12 N (2.7 Å and 3.3 Å) [HB]	Ser195 OG – H_2O – Arg12 N (2.8 Å and 3.1 Å) [HB]	Ser195 OG – H_2O – Arg12 N (2.8 Å and 3.0 Å) [HB]
Ser214 O – H_2O – Arg10 O (2.8 Å and 2.9 Å) [HB]	Ser214 O – H_2O – Arg10 O (2.8 Å and 2.8 Å) [HB]	Ser214 O – H_2O – Arg10 O (2.8 Å and 2.9 Å) [HB]
Ser214 O – H_2O – Arg12 N (2.7 Å and 3.3 Å) [HB]	Ser214 O – H_2O – Asp8 OD2 (3.0 Å and 2.8 Å) [HB]	Ser214 O – H_2O – Arg12 N (2.9 Å and 3.0 Å) [HB]

	Gly216 N – H_2O – Gly17 O (3.1 Å and 2.9 Å) [HB]	Gly216 N – H_2O – Gly17 O (3.3 Å and 2.6 Å) [HB]
	Gly216 O – H_2O – Gly17 O (3.1 Å and 2.9 Å) [HB]	
	Gly216 O – H_2O – Gly13 N (2.7 Å and 2.9 Å) [HB]	Gly216 O – H_2O – Gly13 N (3.0 Å and 2.8 Å) [HB]
Arg217 O – H_2O – Arg12 NH2 (2.7 Å and 3.0 Å) [HB]	Arg217 O – H_2O – Arg12 NH2 (2.7 Å and 3.1 Å) [HB]	Arg217 O – H_2O – Arg12 NH2 (2.7 Å and 3.1 Å) [HB]
Gly218 O – Arg12 NH2 (2.8 Å) [HB]	Gly218 O – Arg12 NH2 (3.2 Å) [HB]	Gly218 O – Arg12 NH2 (3.1 Å) [HB]
Gly218 O – Arg12 NE (2.9 Å) [HB]	Gly218 O – Arg12 NE (3.0 Å) [HB]	Gly218 O – Arg12 NE (3.0 Å) [HB]
Leu222 N – H_2O – Arg12 NH2 (3.0 Å and 3.0 Å) [HB]	Leu222 N – H_2O – Arg12 NH2 (2.9 Å and 3.1 Å) [HB]	Leu222 N – H_2O – Arg12 NH2 (2.9 Å and 3.1 Å) [HB]
Lys224 O – H_2O – Arg12 NH2 (2.9 Å and 3.0 Å) [HB]	Lys224 O – H_2O – Arg12 NH2 (2.9 Å and 3.1 Å) [HB]	Lys224 O – H_2O – Arg12 NH2 (2.8 Å and 3.1 Å) [HB]

Supplementary Table 6. **Polar inter-molecular interactions between huPA and different bicyclic peptides**. Residues and atoms of bicyclic peptides UK18, UK965 and UK970 forming polar inter-molecular interactions with huPA (chymotrypsin numbering). Optimal salt bridges [SB] and hydrogen bond [HB] interactions have been defined using the software LIGPLOT+.[27] Distances of intra-molecular interactions are shown within round brackets.

huPA-UK18 complex	huPA-UK965 complex	huPA-UK970 complex
	Arg35 CZ – Arg4 NH2 (3.78 Å)	Arg35 CZ – Arg4 NH2 (3.65 Å)
	Arg35 NH1 – Arg4 CZ (3.78 Å)	Arg35 NH1 – Arg4 CZ (3.68 Å)
		Arg35 NH2 – Arg4 CB (3.44 Å)
		Arg35 NH2 – Arg4 CG (3.78 Å)
		Arg35 NH2 – Arg4 CZ (3.61 Å)
His37 CG – Tyr5 CE1 (3.55 Å)	His37 CG – Tyr5 CE1 (3.80 Å)	His37 CG – Phe5 CE1 (3.64 Å)
His37 CG – Tyr5 CE2 (3.55 Å)		His37 CG – Phe5 CD1 (3.71 Å)
His37 CG – Tyr5 CD2 (3.70 Å)		His37 ND1 – Phe5 CE1 (3.61 Å)
His37 ND1 – Tyr5 CD2 (3.64 Å)		His37 ND1 – Phe5 CD1 (3.51 Å)
His37 CD2 – Tyr5 CD1 (3.60 Å)	His37 CD2 – Tyr5 CD1 (3.52 Å)	
His37 CD2 – Tyr5 CD2 (3.60 Å)		
His37 CD2 – Tyr5 CE2 (3.53 Å)		
His37 NE2 – Tyr5 CD1 (3.47 Å)	His37 NE2 – Tyr5 CD1 (3.37 Å)	
His37 NE2 – Tyr5 CD2 (3.47 Å)		His37 NE2 – Phe5 CD1 (3.59 Å)
$Gly37^{B}$ CA – Tyr5 OH (3.55 Å)		
$Gly37^{C}$ CA – Tyr5 CE2 (3.53 Å)		
$Gly37^{C}$ CA – Tyr5 OH (3.68 Å)		

Gly37^{C} O – Tyr5 CE2 (3.53 Å)		
Thr39 CG2 – Tyr5 CB (3.90 Å)		Thr39 CG2 – Phe5 O (3.90 Å)
	His57 CD2 – Gly11 N (3.82 Å)	
	His57 NE2 – Gly11 CA (3.55 Å)	His57 NE2 – Gly11 CA (3.65 Å)
Asp60^{A} OD1 – Ala1 CA (3.74 Å)		
	Tyr60^{B} OH – Arg4 CA (3.86 Å)	Tyr60^{B} OH – Arg4 CA (3.75 Å)
	Tyr60^{B} OH – Arg4 CB (3.46 Å)	Tyr60^{B} OH – Arg4 CB (3.20 Å)
		Thr97^{A} O – Gly17 C (3.65 Å)
		Leu97^{B} CA – Gly17 C (3.74 Å)
	His99 CE1 – Gly17 O (3.21 Å)	His99 CE1 – Gly17 O (3.47 Å)
	Gln192 NE2 – Glu6 CG (3.62 Å)	
		Gln192 OE1 – Gly13 C (3.82 Å)
	Gly193 CA – Val7 CG1 (3.89 Å)	Gly193 CA – Val7 CG1 (2.94 Å)

Supplementary Table 7. **Unique non-polar inter-molecular interactions between huPA and different bicyclic peptides**. Residues and atoms of bicyclic peptides UK18, UK965 and UK970 forming unique non-polar inter-molecular interactions with huPA (chymotrypsin numbering). Optimal interactions have been defined using the software LIGPLOT+.[27] Distances of intra-molecular interactions are shown within round brackets.

bicyclic peptide UK18	bicyclic peptide UK965	bicyclic peptide UK970
	Ser3 O – Glu6 N (3.2 Å) [HB]	
Arg4 NH2 – Glu6 OE1 (3.9 Å) [SB]		
Asp8 O – Gly11 N (2.9 Å) [HB]	Asp8 O – Gly11 N (2.9 Å) [HB]	Asp8 O – Gly11 N (3.0 Å) [HB]
Cys9 O – Ser14 OG (3.2 Å) [HB]	Cys9 O – Cys16 N (3.0 Å) [HB]	Cys9 O – Cys16 N (3.1 Å) [HB]
Gly11 O – Ser14 OG (2.9 Å) [HB]	Gly11 O – Gly14 N (2.7 Å) [HB]	Gly11 O – Gly14 N (2.7 Å) [HB]
	Gly14 O – Gly17 N (3.3 Å) [HB]	Gly14 O – Gly17 N (3.3 Å) [HB]

Supplementary Table 8. **Polar intra-molecular interactions within different bicyclic peptides**. Residues and atoms forming polar intra-molecular interactions in bicyclic peptides UK18, UK965 and UK970. Optimal salt bridges [SB] and hydrogen bond [HB] interactions have been defined using the software PyMOL.[28] Distances of intra-molecular HB interactions are shown within round brackets.

Supplementary figures

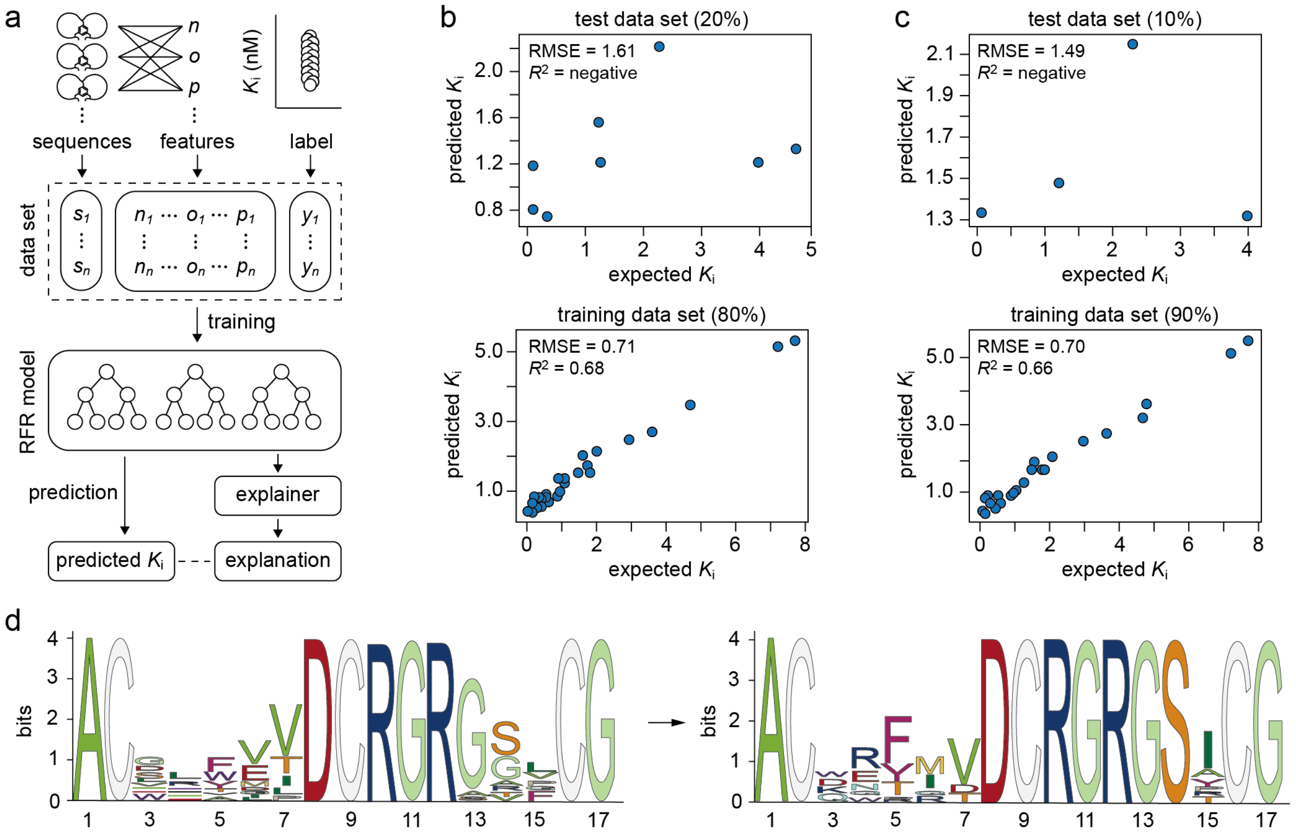

Supplementary Figure 1. **RFR algorithm development flowchart, metrics evaluation and amino acid MSA logo**. **a**) Schematic representation of the Random Forest Regression (RFR) algorithm process flow; **b, c**) Root mean squared error (RMSE) and linear coefficient (R^2) evaluation of two different test and training dataset. RMSE and R^2 have been used to evaluate the model predictions and find the best dataset distribution aiming at optimizing the K_i predictions while avoiding overfitting; **d**) MSA logo of the family of 37 phage-encoded bicyclic peptides inhibitors of huPA, whose most potent inhibitor is UK18 (left side) and MSA logo of the best bicyclic peptide binders predicted by RFR method (right side) among the randomly generated sequences.

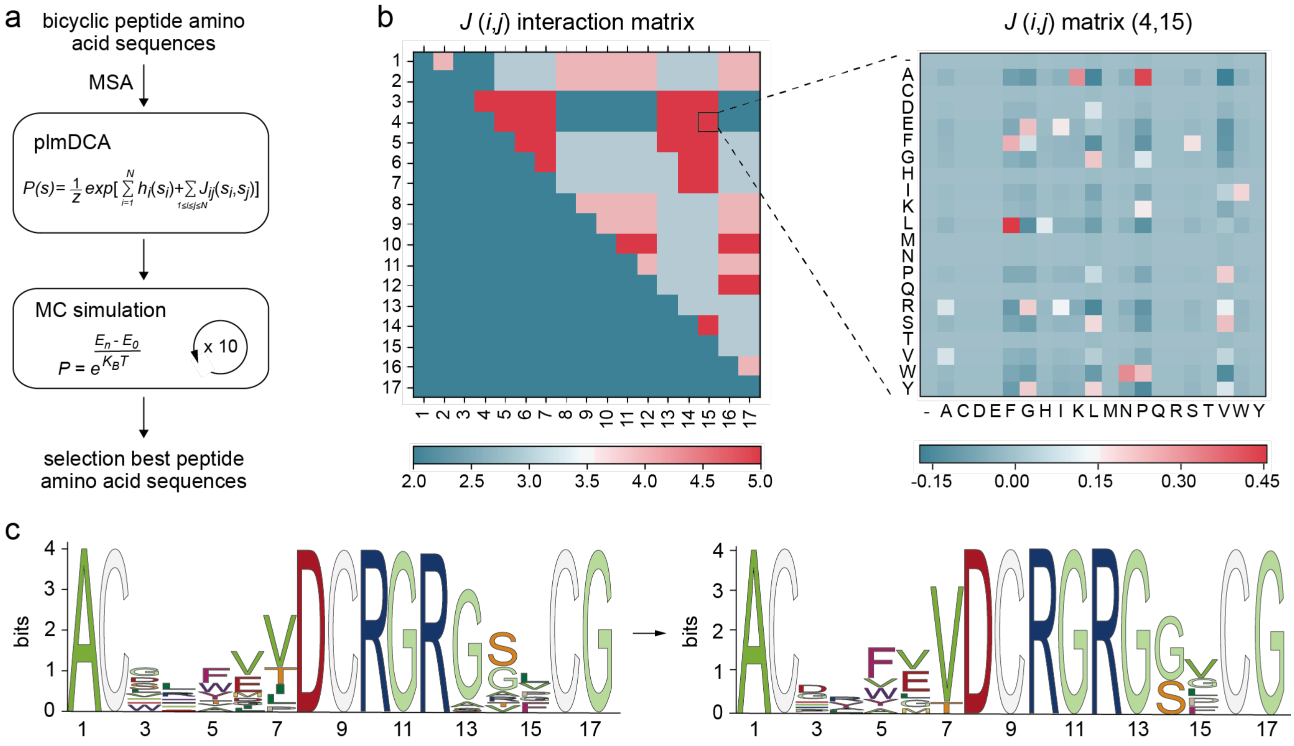

Supplementary Figure 2. **PlmDCA method coupled to MC simulation flowchart, interaction matrices and amino acid MSA logo**. **a**) Schematic representation of the pseudolikelihood maximization directed coupling analysis (plmDCA) method combined to Monte Carlo (MC) simulation process flow; **b**) Interaction matrix example in which red squares in the heatmaps indicate a strong correlation while blue squares designate low correlation between couples of amino acids. Each correlation score given for a couple of positions corresponds to the Frobenius norm of a submatrix as indicated on the right side in which the best amino acids correlations are evaluated; **c**) MSA logo of the family of 37 phage-encoded bicyclic peptides inhibitors of huPA, whose most potent inhibitor is UK18 (left side)[17] and MSA logo of novel sequences generated through the plmDCA and MC combined methods (right side).

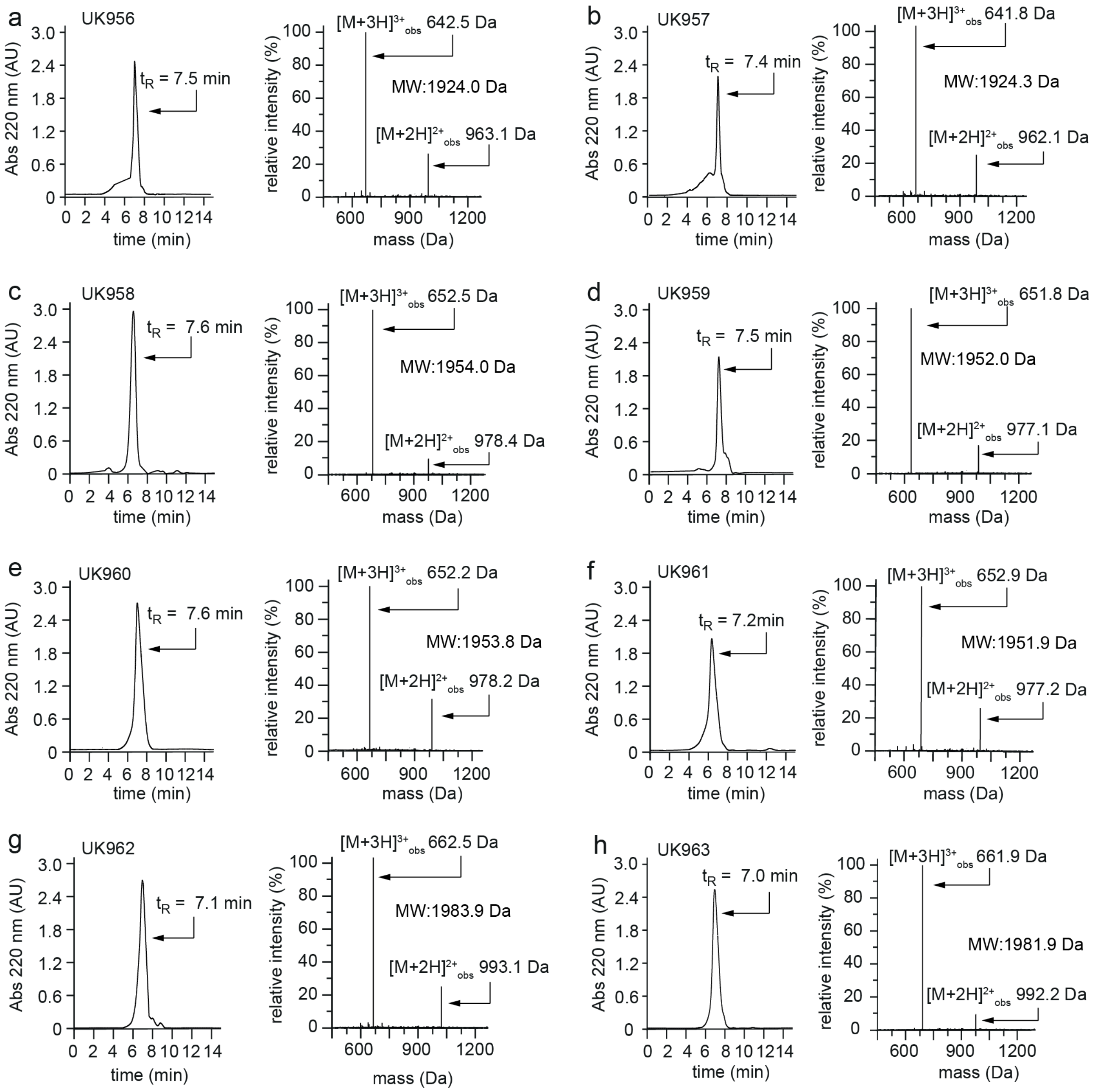

Supplementary figure 3. **Synthesis and characterisation of bicyclic peptides UK956-UK963**. HPLC (left) and mass spectra (right) analysis of bicyclic peptides UK956 (**a**), UK957 (**b**), UK958 (**c**), UK959 (**d**), UK960 (**e**), UK961 (**f**), UK962 (**g**) and UK963 (**h**). The measured molecular weight of each bicyclic peptide corresponds to the expected mass. Name, elution retention time (t_R), expected molecular weight (M.W.) and observed molecular ions of each peptide are indicated.

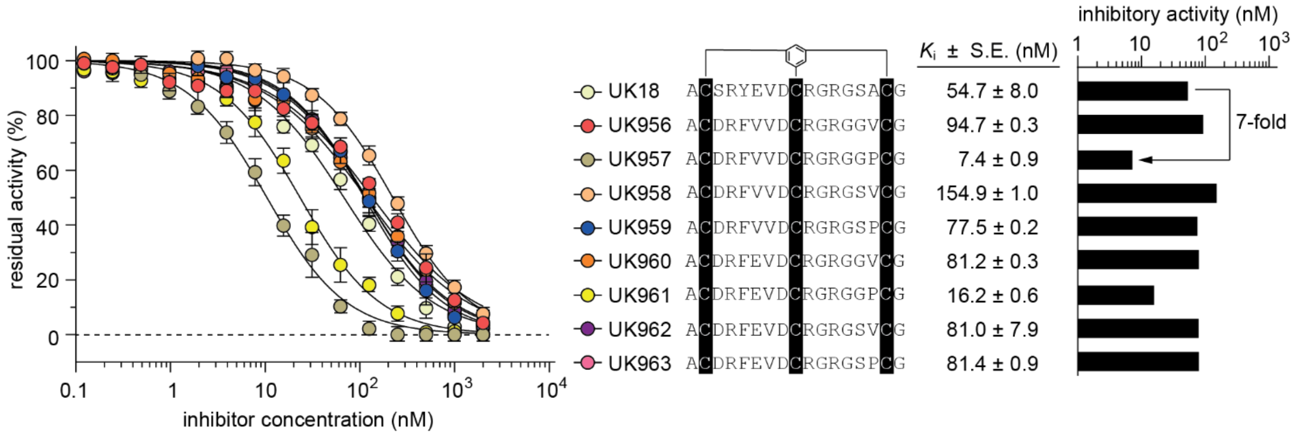

Supplementary figure 4. **Activity assay of bicyclic peptides UK956-UK963**. Residual activities of huPA measured at different concentrations of bicyclic peptides UK18, UK956, UK957, UK958, UK959, UK960, UK961, UK962 and UK963. The indicated values are the means of three independent experiments. Data are presented as mean (symbol). S.E., standard error and at the right the column graph comparing the determined K_i values. The inhibitory activities of all peptide variants towards huPA were determined at 25 °C and physiological pH (7.4) using the suitable substrate at a concentration of 50 µM. The K_m value of huPA protease was determined by standard Michaelis-Menten kinetics and used in the calculation of the reported K_i values. S.E., standard error.

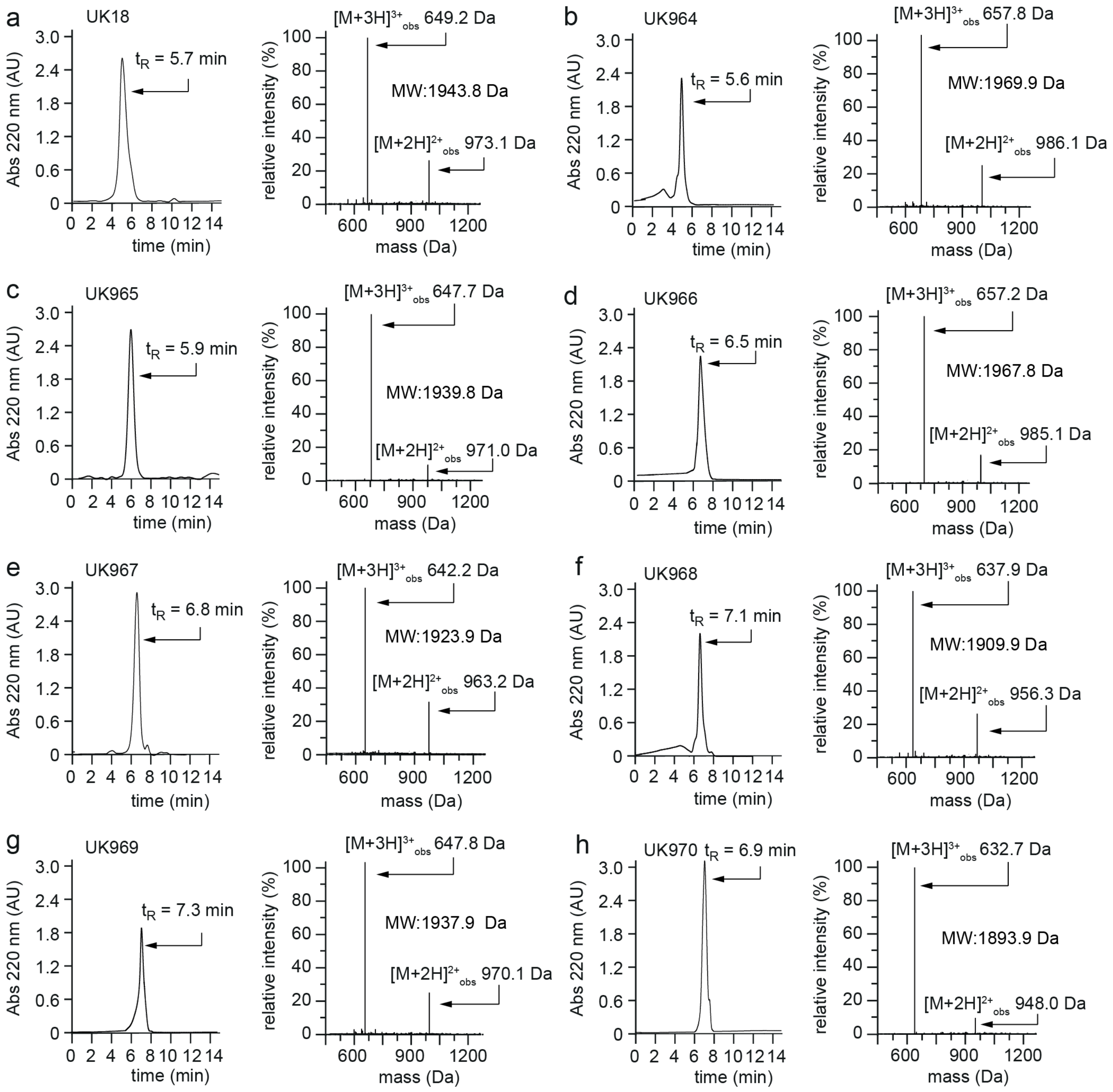

Supplementary figure 5. **Synthesis and characterisation of bicyclic peptides UK18 and UK964-UK970**. HPLC (left) and mass spectra (right) analysis of bicyclic peptides UK18 (**a**), UK964 (**b**), UK965 (**c**), UK966 (**d**), UK967 (**e**), UK968 (**f**), UK969 (**g**) and UK970 (**h**). The measured molecular weight of each bicyclic peptide corresponds to the expected mass. Name, elution retention time (t_R), expected molecular weight (M.W.) and observed molecular ions of each peptide are indicated.

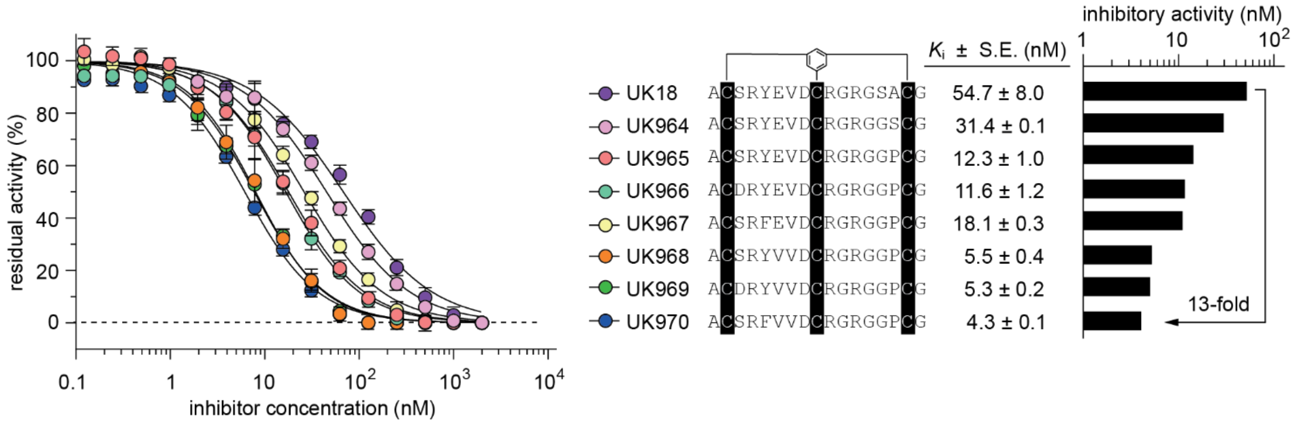

Supplementary figure 6. **Activity assay of bicyclic peptides UK18 and UK964-UK970**. Residual activities of huPA measured at different concentrations of bicyclic peptides UK18, UK964, UK965, UK966, UK967, UK968, UK969 and UK970. The indicated values are the means of three independent experiments. Data are presented as mean (symbol). S.E., standard error and at the right the Column graph comparing the determined K_i values. The inhibitory activities of all peptide variants towards huPA were determined at 25 °C and physiological pH (7.4) using the suitable substrate at a concentration of 50 µM. The K_m value of huPA protease was determined by standard Michaelis-Menten kinetics and used in the calculation of the reported K_i values. S.E., standard error.

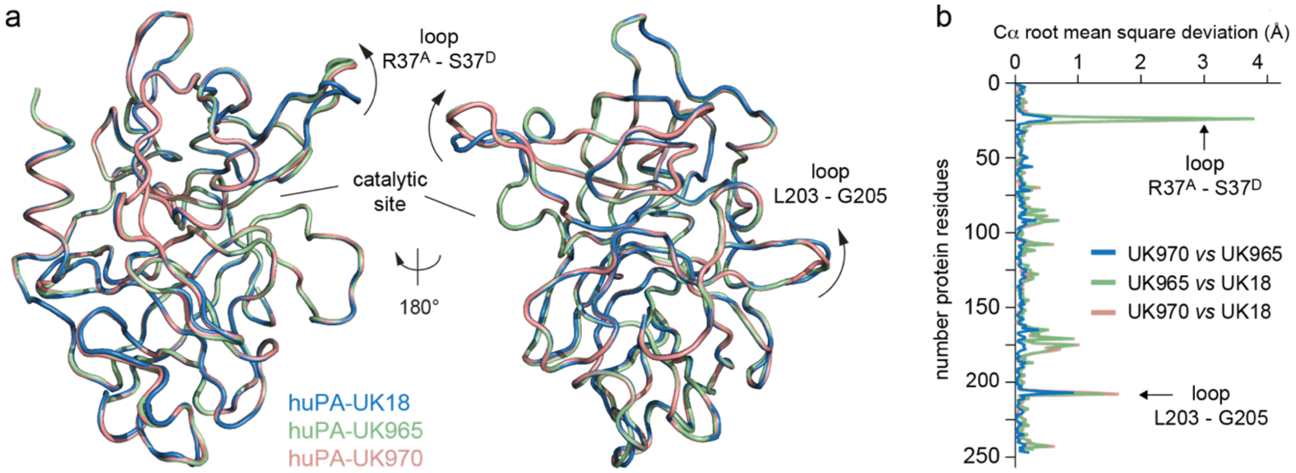

Supplementary figure 7. **Superimposition of huPA-UK18, huPA-UK965 and huPA-UK970 crystal structure complexes**. **a**) Superimposition of aligned huPA-UK18 (blue), huPA-UK965 (pale green) and huPA-UK970 (salmon) binary complexes are shown in two orientations (180° rotation). The secondary structures of huPA are represented by ribbon diagram. The three-dimensional structure was generated and rendered using PyMOL[28]; **b**) Root mean square deviations (RMSDs) calculated over the C-alpha atoms for the different complexes have been determined using Gesamt algorithm.[20]

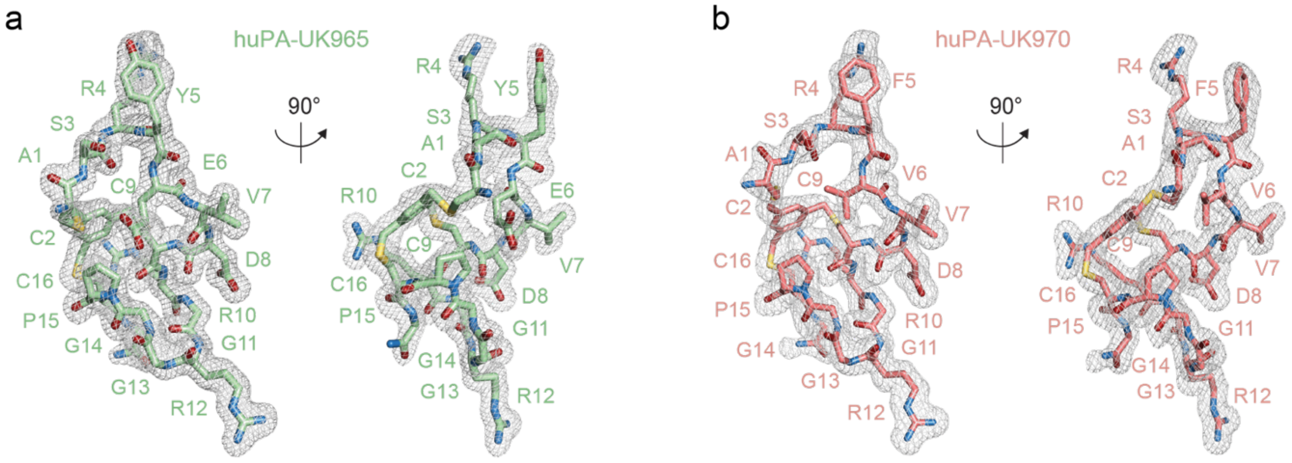

Supplementary figure 8. **Conformation and electron density map of bicyclic peptides UK965 and UK970**. **a**) Conformation and electron density map of bicyclic peptide UK965 shown in two orientations (90° rotation). The aromatic ring of the mesitylene core and the side chains of the residues are shown as sticks. Carbon, oxygen, nitrogen, and sulphur atoms are shown in pale green, red, blue and yellow, respectively; **b**) Conformation and electron density map of bicyclic peptide UK970 shown in two orientations (90° rotation). The aromatic ring of the mesitylene core and the side chains of the residues are shown as sticks. Carbon, oxygen, nitrogen, and sulphur atoms are shown in salmon, red, blue, and yellow, respectively. The $2F_o - F_c$ electron density maps are shown and contoured at the 2σ level. The three-dimensional structures were generated and rendered using PyMOL.[28]

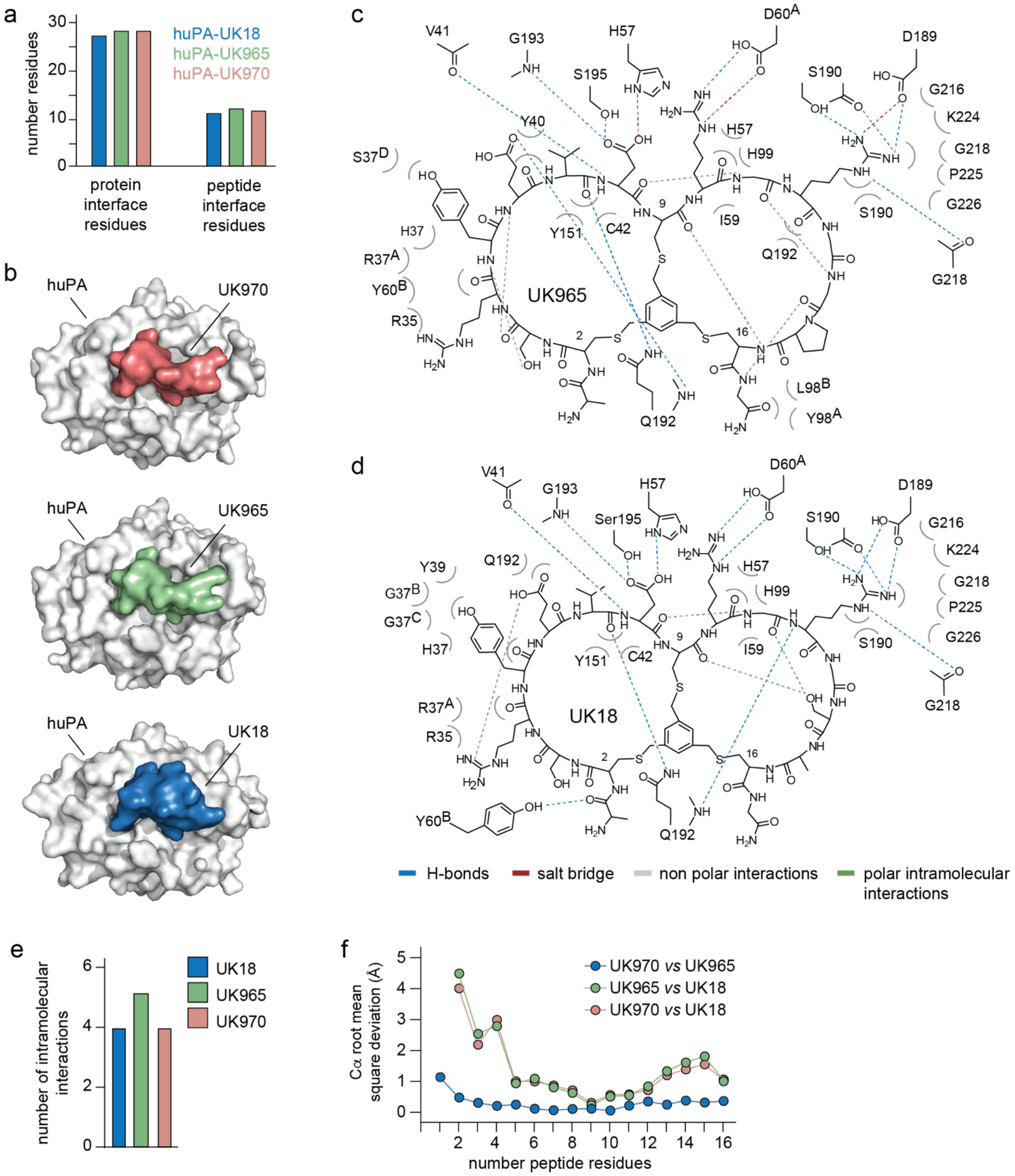

Supplementary figure 9. **Structural comparison of the binding mode of bicyclic peptides UK18, UK965 and UK970 in complex with huPA**. **a**) Column graph comparing the number of protein interface residues that interact with the three bicyclic peptides (UK18 in blue, UK965 in pale green and UK970 in salmon) and the number of peptides interface residues that interact with huPA; **b**) Surface representation of huPA (grey) in complex with the three bicyclic peptides (UK18 in blue, UK965 in pale green and UK970 in salmon). The three-dimensional structures were generated and rendered using PyMOL;[28] **c**) Schematic representation of molecular interactions between human

uPA and UK965. Residues of human uPA are labelled according to the chymotrypsin numbering system. Salt bridge (red) and intramolecular (blue) hydrogen bonds are shown as dashed lines. Bent grey lines indicate residues of UK965 in close contact with human uPA (distances shorter than 4.0 Å that are not hydrogen bonds); **d**) Schematic representation of molecular interactions between human uPA and UK18. Residues of human uPA are labelled according to the chymotrypsin numbering system. Salt bridge (red) and intramolecular (blue) hydrogen bonds are shown as dashed lines. Bent grey lines indicate residues of UK18 in close contact with human uPA (distances shorter than 4.0 Å that are not hydrogen bonds); **e**) Column graph comparing the number of intramolecular interactions of the three peptides (UK18 in blue, UK965 in green and UK970 in pink) with the huPA; **f**) Root mean square deviations (RMSDs) calculated over the C-alpha atoms for the three different bicyclic peptides (UK18 in blue, UK965 in pale green and UK970 in salmon) in comparison to each other have been determined using Gesamt algorithm.[20]

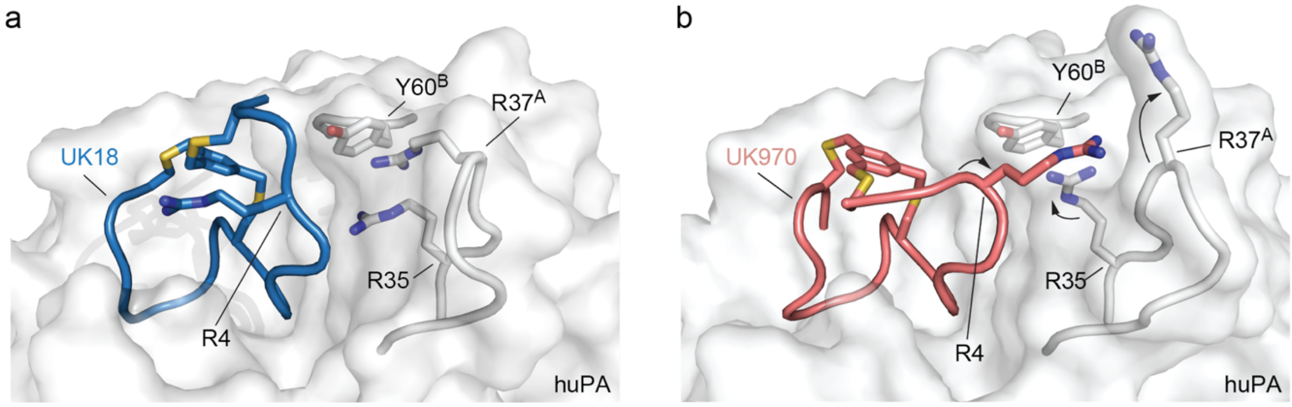

Supplementary figure 10. **Differences in the binding mode of bicyclic peptides UK18 and UK970 to huPA**. Detail view of previously solved X-ray structure of bicyclic peptide UK18 in complex with huPA (blue and grey, **a**) and bicyclic peptide UK970 in complex with huPA (salmon and grey, **b**). The large conformational change induced by Pro15, located in the second loop, causes a repositioning of the Arg4 side chain, located in the first loop, that can now form additional contacts with huPA residues Arg35 and Tyr60^{B}, not engaged in the huPA-UK18 complex. The three-dimensional structures were generated and rendered using PyMOL.[28]

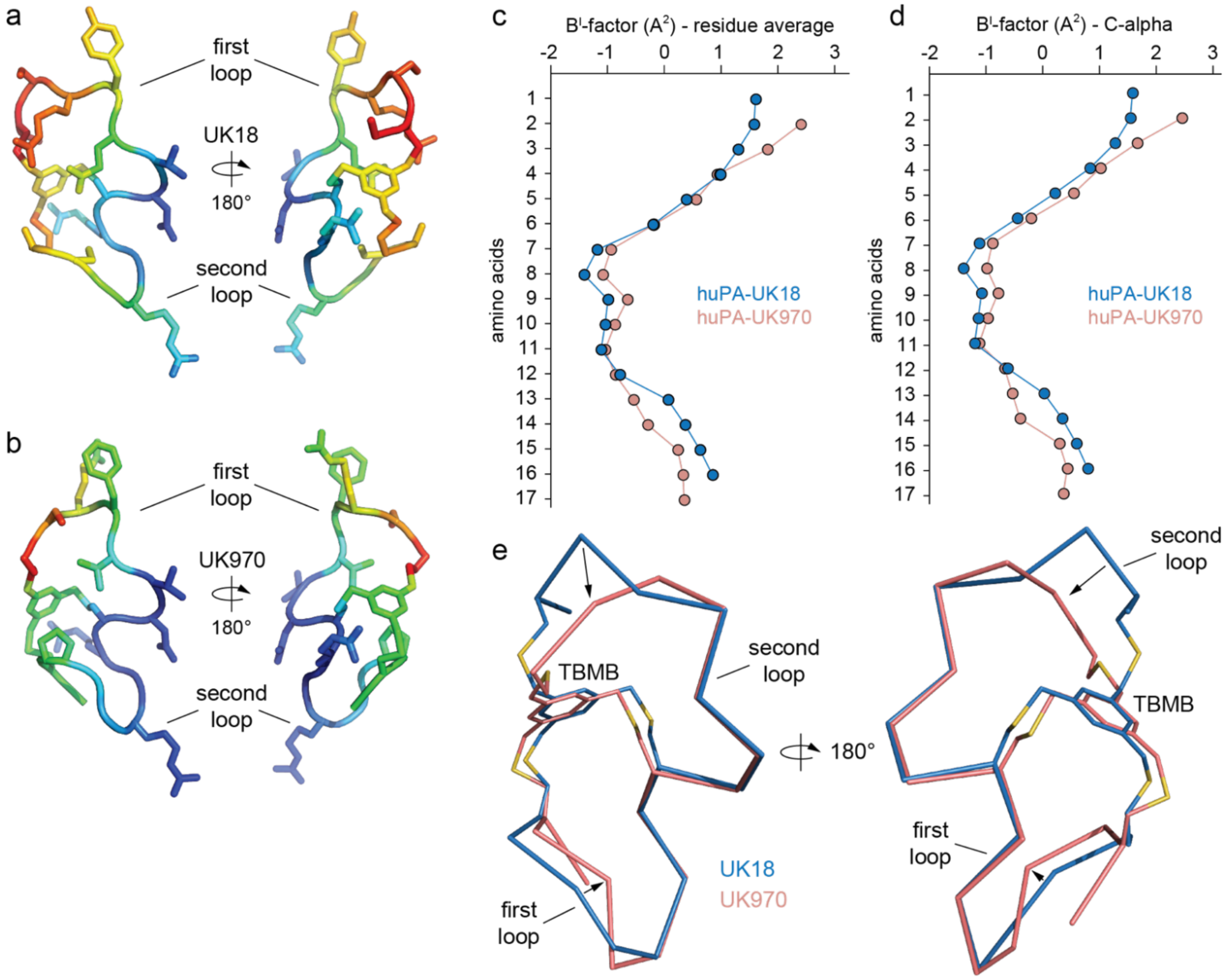

Supplementary figure 11. B-factor diagram of bicyclic peptide UK18 and UK970 in complex with huPA. B-factor diagram of bicyclic peptides UK18 (**a**) and UK970 (**b**) shown in two orientations (180° rotation). The aromatic ring of the mesitylene core and the side chains of the residues are shown as sticks. The B^{I}-factor values are illustrated by colour, ranging from low (blue) to high (red). B^{I}-factor values of the residue atoms (**c**) and of the C-alpha carbon atoms (**d**) of bicyclic peptide UK18 (blue) and UK970 (salmon) in complex with huPA as a function of the peptide residue number; **e**) Superimposition of aligned bicyclic peptide UK18 (blue) and UK970 (salmon) shown in two orientations (180° rotation). The three-dimensional structures were generated and rendered using PyMOL.[28] Normalised B-factor (B^{I}-factor) for structural comparison has been determined using BAN∆IT.[29]

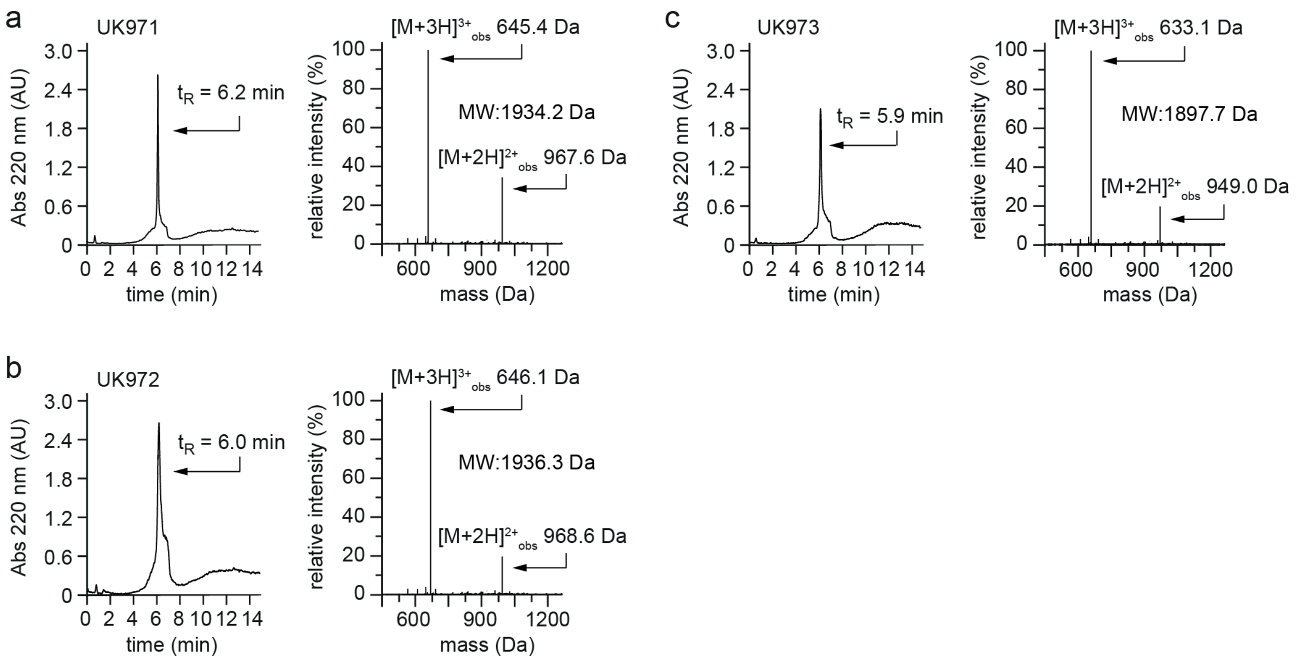

Supplementary figure 12. **Synthesis and characterisation of bicyclic peptides UK18 and UK964-UK970**. HPLC (left) and mass spectra (right) analysis of bicyclic peptides UK971 (**a**), UK972 (**b**) and UK973 (**c**). The measured molecular weight of each bicyclic peptide corresponds to the expected mass. Name, elution retention time (t_R), expected molecular weight (M.W.) and observed molecular ions of each peptide are indicated.

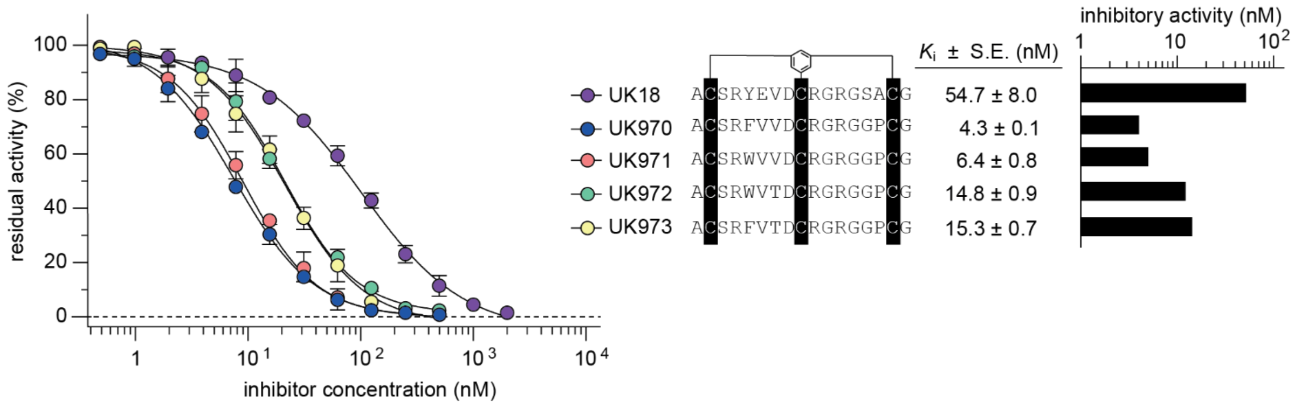

Supplementary figure 13. **Activity assay of bicyclic peptides UK18 and UK970-UK973**. Residual activities of huPA measured at different concentrations of bicyclic peptides UK18, UK970, UK971, UK972 and UK973. The indicated values are the means of three independent experiments. Data are presented as mean (symbol). S.E., standard error and at the right the Column graph comparing the determined K_i values. The inhibitory activities of all peptide variants towards huPA were determined at 25 °C and physiological pH (7.4) using the suitable substrate at a concentration of 50 µM. The K_m value of huPA protease was determined by standard Michaelis-Menten kinetics and used in the calculation of the reported K_i values. S.E., standard error.

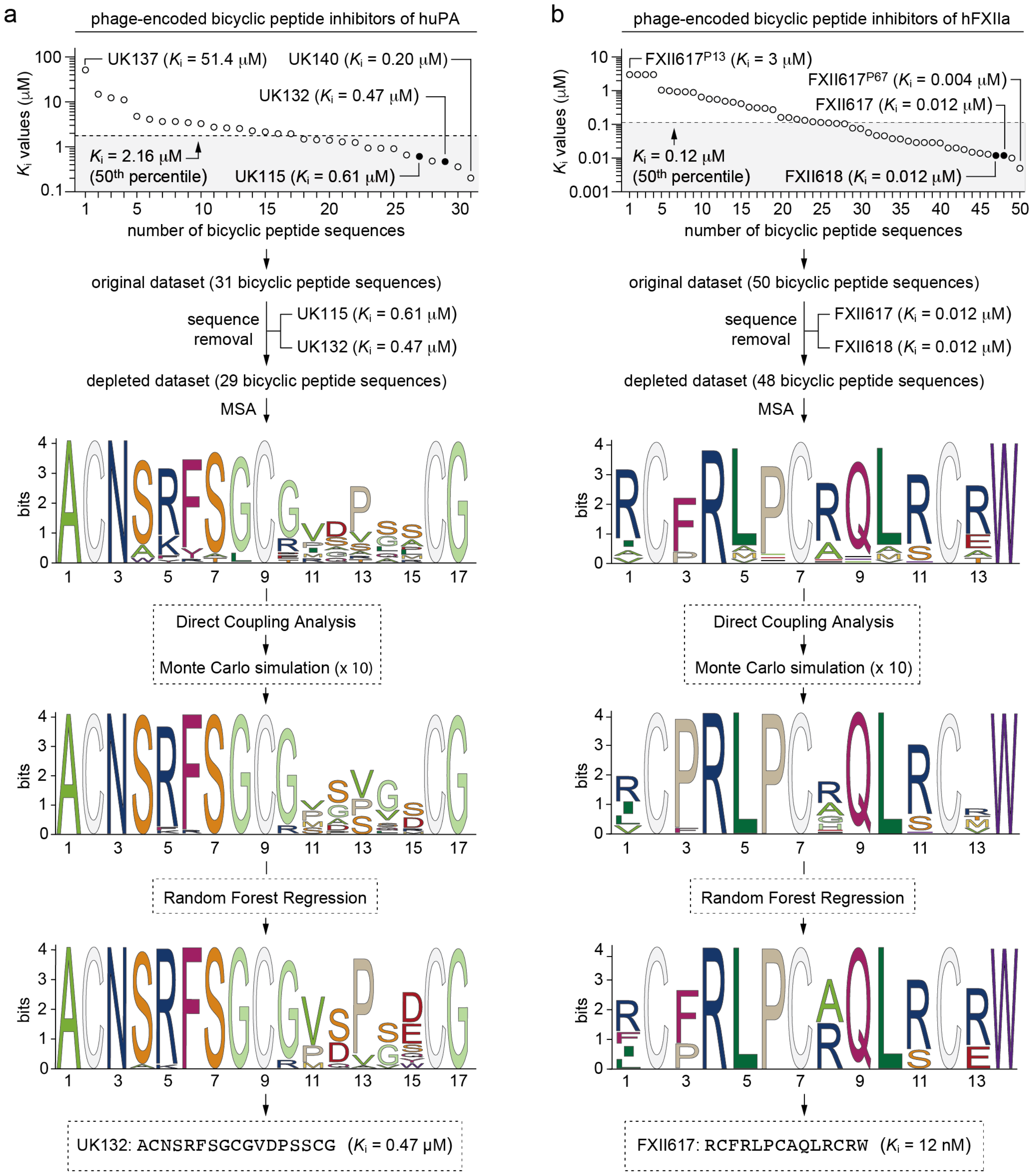

Supplementary figure 14. *In silico* molecular evolution of two different families of phage-encoded bicyclic peptide inhibitors that have been depleted of two bicyclic peptide sequences each. a) Top, plot reporting the inhibitory constant (K_i) values (*y* axis) of the 31 unique phage-encoded bicyclic peptides (*x* axis) selected *in vitro* against huPA. The bicyclic peptide molecules (white dots) are shown in descending order, starting from the weakest (left, peptide sequence 1, bicyclic peptide UK137; K_i = 51.4 μM) to the most powerful one (right, peptide sequence 31, bicyclic peptide UK140; K_i = 0.20 μM). The bicyclic peptide inhibitors removed are indicated as

black dots and are UK115 (K_i = 0.61 μM) and UK132 (K_i = 0.47 μM). MSA logo of 29 phage-encoded bicyclic peptides (input data) selected *in vitro* against huPA upon depletion of two bicyclic peptide sequences: UK115 and UK132. Combination of pseudolikelihood maximization direct coupling analysis (plmDCA) and Monte Carlo (MC) methods yielded ~1700 new peptide sequences (middle MSA logo). Further selection using Random Forest Regression (RFR) algorithm yielded 63 new bicyclic peptide sequences with a preferential frequency of amino acids at each position and predicted to have K_i values below 2.16 μM (that corresponds to 50th percentile; bottom MSA logo). The combined statistical and computational approach was able to generate *de novo* UK132 (K_i = 0.47 μM), the most potent inhibitor of the two initially eliminated bicyclic peptide sequences; **b**) Top, plot reporting the inhibitory constant (K_i) values (*y* axis) of the 50 unique phage-encoded bicyclic peptides (x axis) selected *in vitro* against human coagulation factor XIIa (hFXIIa). The bicyclic peptide molecules (white dots) are shown in descending order, starting from the weakest (left, peptide sequence 1, bicyclic peptide FXII617^{P13}; K_i = 3 μM) to the most powerful one (right, peptide sequence 50, bicyclic peptide FXII617^{P67}; K_i = 0.004 μM). The bicyclic peptide inhibitors removed are indicated as black dots and are FXII617 (K_i = 0.012 μM) and FXII618 (K_i = 0.012 μM). MSA logo of 48 phage-encoded bicyclic peptides (input data) selected *in vitro* against hFXIIa upon depletion of two bicyclic peptide sequences: FXII617 and FXII618. Combination of pseudolikelihood maximization direct coupling analysis (plmDCA) and Monte Carlo (MC) methods yielded ~230 new peptide sequences (middle MSA logo). Further selection using Random Forest Regression (RFR) algorithm yielded 6 new bicyclic peptide sequences with a preferential frequency of amino acids at each position and predicted to have K_i values below 0.12 μM (that corresponds to 50th percentile; bottom MSA logo). The combined statistical and computational approach was able to generate *de novo* FXII617 (K_i = 12 nM), the more potent of the two initially eliminated bicyclic peptide inhibitors.

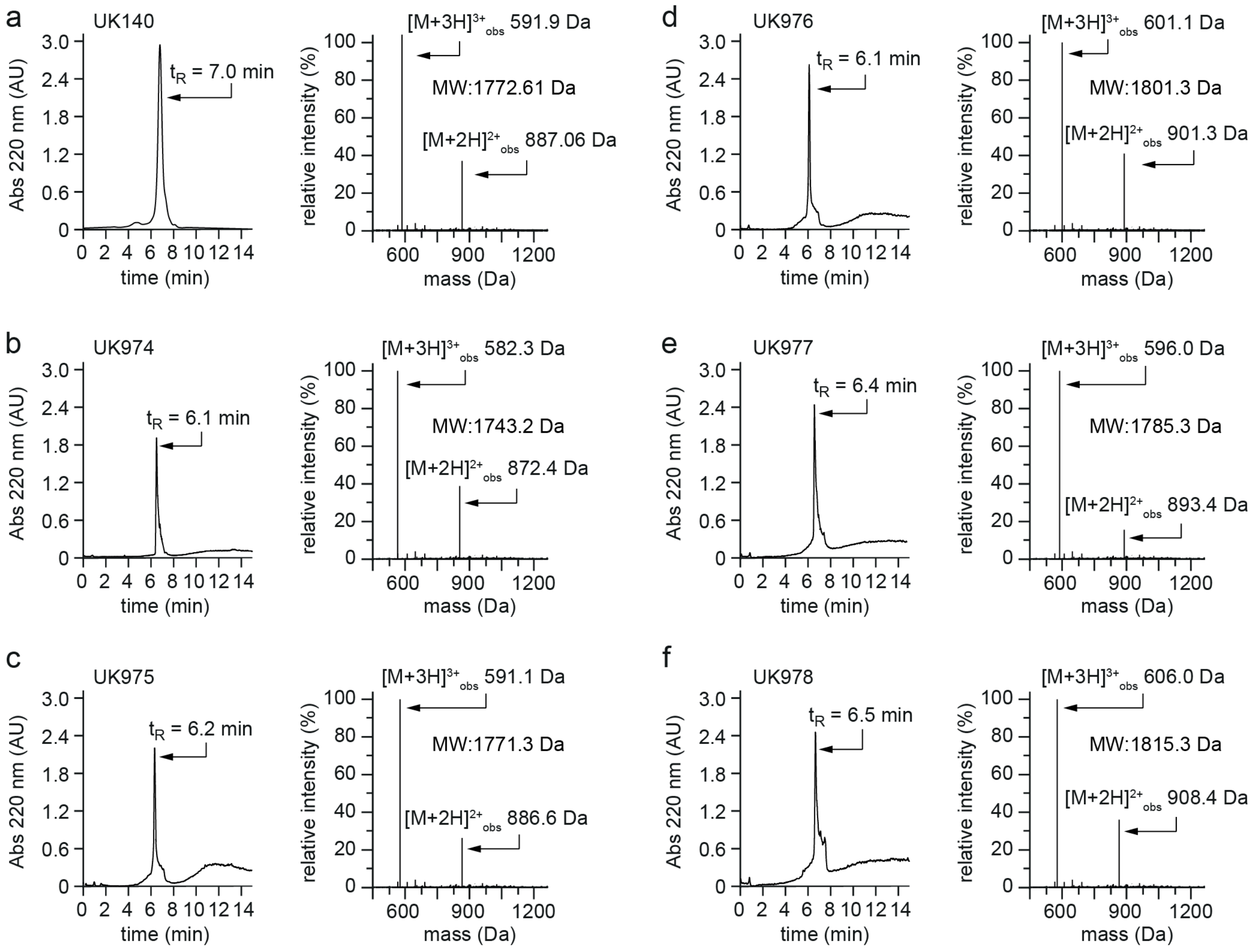

Supplementary figure 15. **Synthesis and characterisation of bicyclic peptides UK140 and UK974-UK978**. HPLC (left) and mass spectra (right) analysis of bicyclic peptides UK140 (**a**), UK974 (**b**), UK975 (**c**), UK976 (**d**), UK977 (**e**) and UK978 (**f**). The measured molecular weight of each bicyclic peptide corresponds to the expected mass. Name, elution retention time (t_R), expected molecular weight (M.W.) and observed molecular ions of each peptide are indicated.

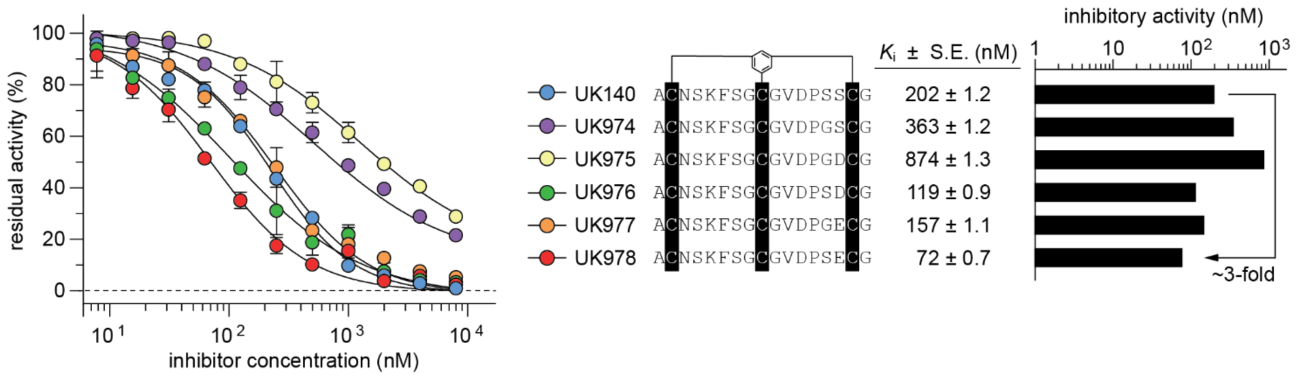

Supplementary figure 16. **Activity assay of bicyclic peptides UK140 and UK974-UK978**. Residual activities of huPA measured at different concentrations of bicyclic peptides UK140, UK974, UK975, UK976, UK977 and UK978. The indicated values are the means of three independent experiments. Data are presented as mean (symbol). S.E., standard error and at the right the Column graph comparing the determined K_i values. The inhibitory activities of all peptide variants towards huPA were determined at 25 °C and physiological pH (7.4) using the suitable substrate at a concentration of 50 µM. The K_m value of huPA protease was determined by standard Michaelis-Menten kinetics and used in the calculation of the reported K_i values. S.E., standard error.

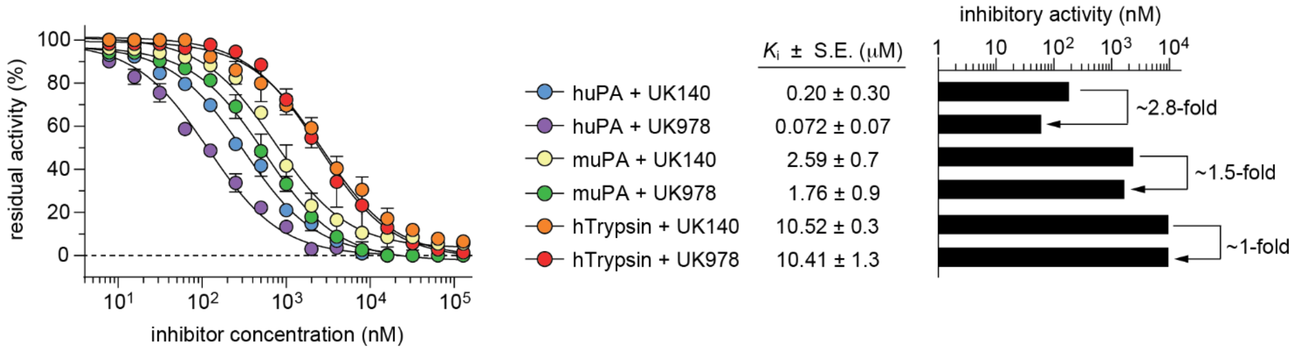

Supplementary figure 17. **Activity assay of bicyclic peptides UK140 and UK978 against human uPA, murine uPA and human trypsin**. Residual activities of human uPA (huPA), murine muPA (muPA) and human trypsin (hTryp) measured at different concentrations of bicyclic peptides UK140 and UK978. The indicated values are the means of three independent experiments. Data are presented as mean (symbol). S.E., standard error and at the right the Column graph comparing the determined K_i values. The inhibitory activities of synthetic peptide UK140 and UK978 towards huPA, muPA and hTryp were determined at 25 °C and physiological pH (7.4) using the suitable substrate at a concentration of 50 µM. The K_m value of each protease was determined by standard Michaelis-Menten kinetics and used in the calculation of the reported K_i values. S.E., standard error.

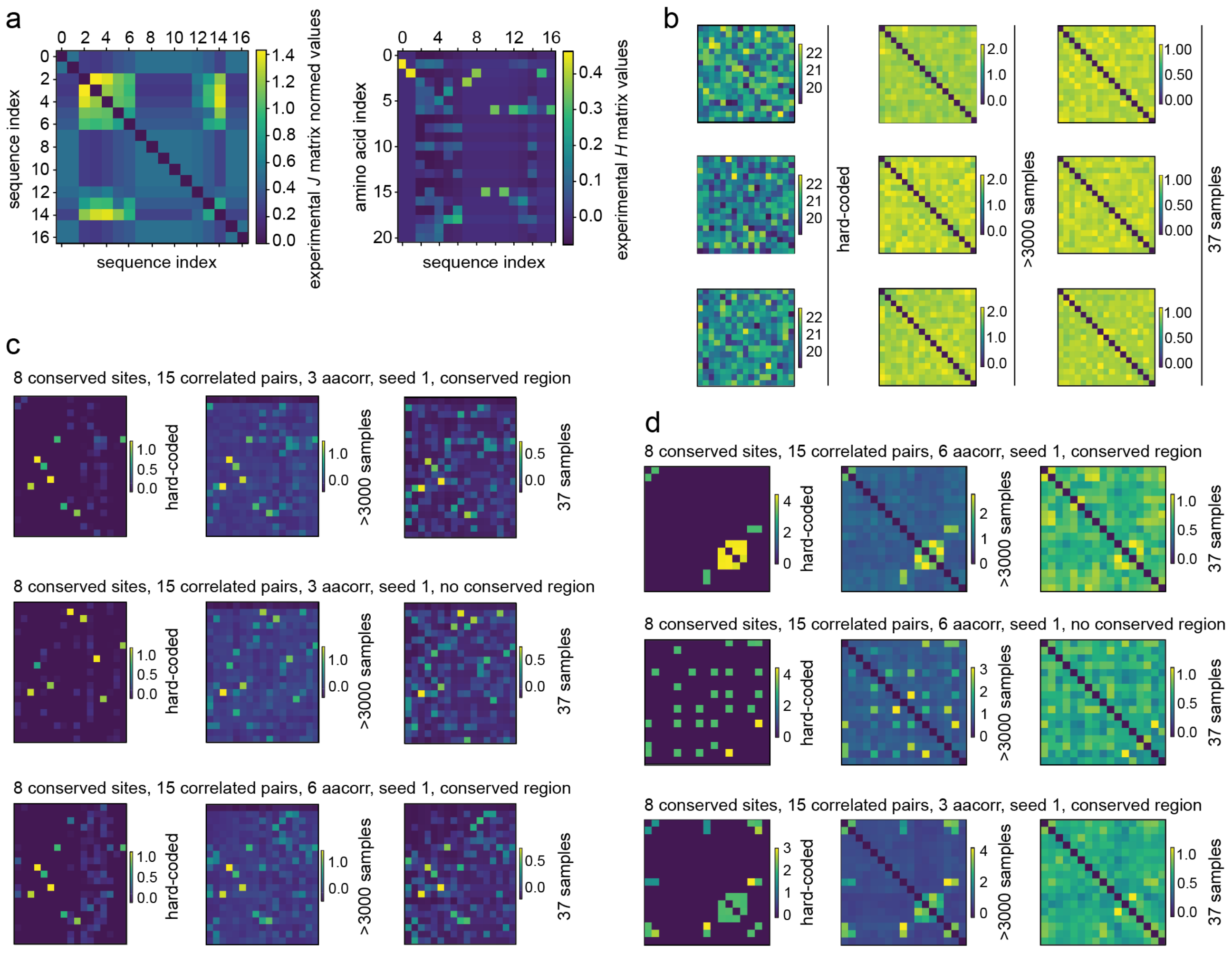

Supplementary figure 18. **DCA parameter representations**. **a**) Matrices from training the DCA model on 37 experimentally characterized sequences. Left: reduced representation of the *J* tensor (17 × 17 × 21 × 21) by taking the norm. Right: *h* matrix representing conserved amino acid sites; **b**) Three *J* matrices with elements sampled from a normal distribution and normed. Columns: 1. Hardcoded random matrices. 2. DCA model trained on >3000 sequences generated from hardcoded matrices. 3. DCA model trained on 37 sequences sampled from the generated sequences; **c-d**) Three *H* and *J* matrices rationally hardcoded to represent peptide families with varied parameters, following the same column pattern as the random DCA parameters. The third column of the normed *J* tensor displays the DCA model's ability to learn correlations between sequence indexes, especially at correlated regions.

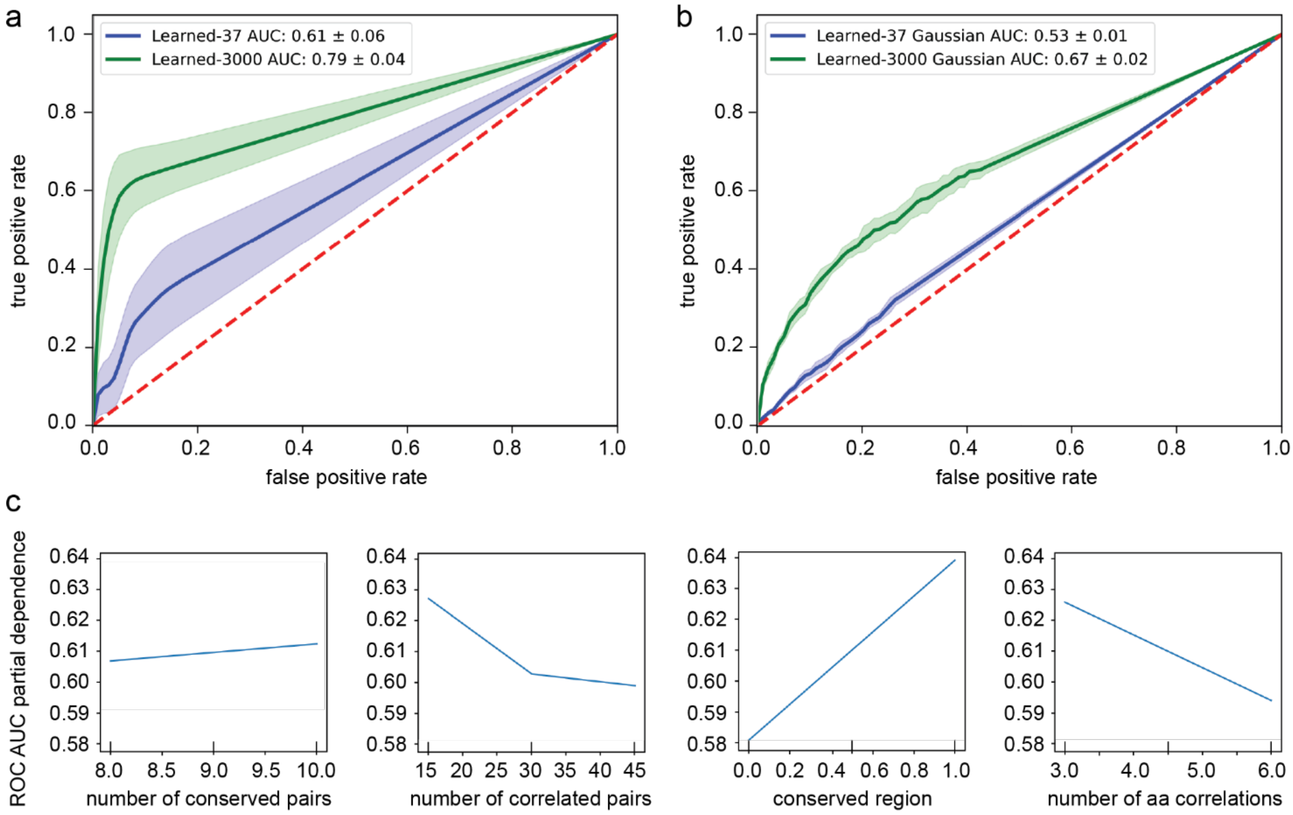

Supplementary figure 19. **Receiver Operating Characteristic (ROC) analysis**. **a**) Average ROC curve across 72 *in silico* peptide families with one standard deviation shown as shaded region. Red line: ROC curve for random DCA discrimination. Blue line: DCA model trained on 37 sequences. Green line: DCA model trained on >3000 sequences. Legend: Average area under the curve (AUC) with one standard deviation; **b**) Average ROC curve for DCA parameters learned from a Gaussian distribution. Red, blue, and green curves represent the same groups as in panel 'a'; **c**) ROC AUC against *in silico* peptide parameters. Panel 'a' demonstrates that training a DCA model on 37 sequences enables the model to learn meaningfully information about the underlying peptide family, obtaining a ROC AUC of 0.61 on average. Comparing panel 'a' and 'b' shows that the peptide families properties make it easier for a DCA model to learn when compared to sequences with random underlying distributions. Panel 'c' shows that as a peptide family's J matrix increases in complexity the harder it is to learn, and that the presence of a conserved region contributes the most in enabling the model to learn the parameters.

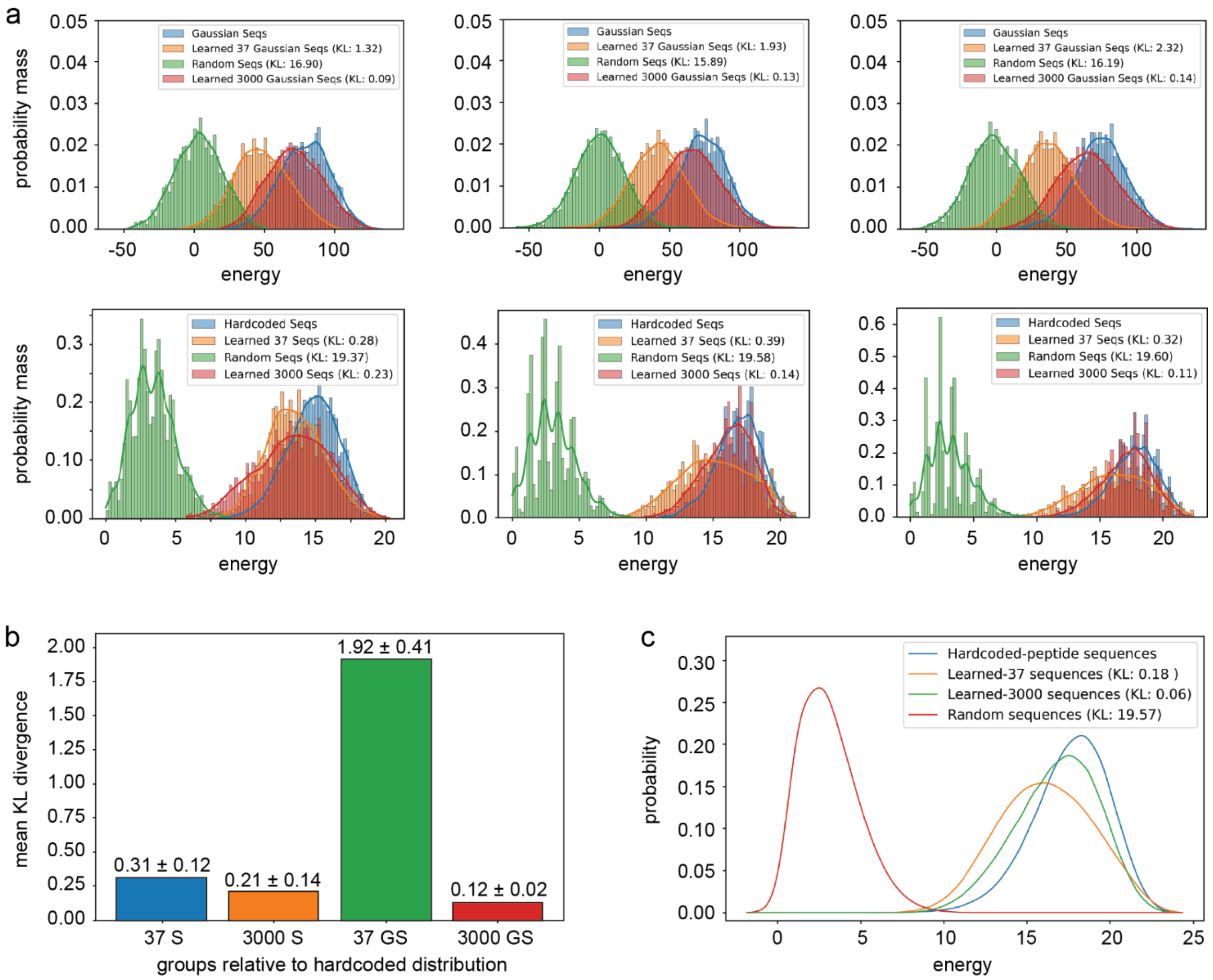

Supplementary figure 20. **Kullback-Leibler divergence (KL-div) analysis**. **a**) Top: energy distributions from Gaussian sequence sets. Bottom: energy distributions from three representative peptide sequence sets. Green distribution (Random Seqs) is a negative control from randomly generated sequences. Energies are evaluated using the respective hardcoded parameter sets. Legend: KL-div values calculated relative to the energy distribution of the hardcoded sequence sets; **b**) Mean KL-div values for 72 learned-37 sequence and 3000 sequence sets (S), and three learned-37 and learned-3000 Gaussian sequence sets (GS). Mean and standard deviation are shown above each bar; **c**) Mean Gaussian kernel density estimation plots with KL-Div from mean plots.

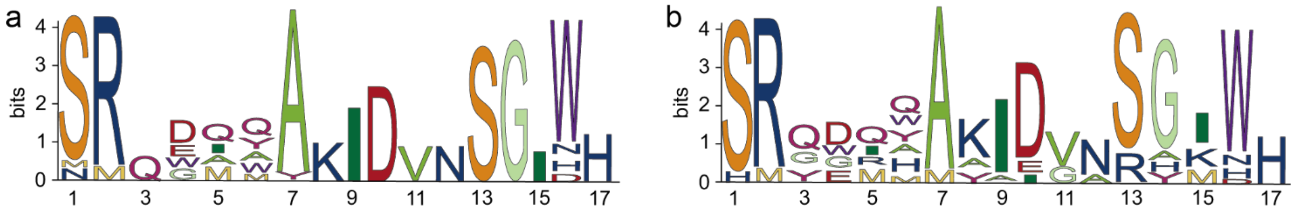

Supplementary figure 21. MSA Logos from sequences generated from hardcoded-peptide parameters with 10 conserved sites, 45 amino acid correlations, 3 amino acids per correlation, and a conserved region. **a**) MSA logo generated from 3000 sequences; **b**) MSA logo generated from 37 sequences randomly sampled from the 3000 sequences.

References

(1) Yang, K. K.; Wu, Z.; Arnold, F. H. Machine-Learning-Guided Directed Evolution for Protein Engineering. *Nature Methods* **2019**, *16* (8), 687–694. https://doi.org/10.1038/s41592-019-0496-6.

(2) Breiman, L. Random Forests. *Machine Learning* **2001**, *45*, 5–32. https://doi.org/https://doi.org/10.1023/A:1010933404324.

(3) Breiman, L. *Classification and Regression Trees*; Routledge, Ed.; 1984. https://doi.org/https://doi.org/10.1201/9781315139470.

(4) Breiman, L. Technical Report 670 STATISTICS DEPARTMENT. *Statistics* **2004**.

(5) Pedregosa, F.; Varoquaux, G.; Gramfort, A.; Michel, V.; Thirion, B.; Grisel, O.; Blondel, M.; Prettenhofer, P.; Weiss, R.; Dubourg, V.; Vanderplas, J.; Passos, A.; Cournapeau, D.; Brucher, M.; Perrot, M.; Duchesnay, É. Scikit-Learn: Machine Learning in Python. *Journal of Machine Learning Research* **2011**, *12*, 2825–2830.

(6) Müller, A. T.; Gabernet, G.; Hiss, J. A.; Schneider, G. ModlAMP: Python for Antimicrobial Peptides. *Bioinformatics (Oxford, England)* **2017**, *33* (17), 2753–2755. https://doi.org/10.1093/bioinformatics/btx285.

(7) Ribeiro, M. T.; Singh, S.; Guestrin, C. "Why Should I Trust You?" Explaining the Predictions of Any Classifier. *NAACL-HLT 2016 - 2016 Conference of the North American Chapter of the Association for Computational Linguistics: Human Language Technologies, Proceedings of the Demonstrations Session* **2016**, 97–101. https://doi.org/10.18653/v1/n16-3020.

(8) Lundberg, S. M.; Erion, G.; Chen, H.; DeGrave, A.; Prutkin, J. M.; Nair, B.; Katz, R.; Himmelfarb, J.; Bansal, N.; Lee, S. I. From Local Explanations to Global Understanding with Explainable AI for Trees. *Nature Machine Intelligence* **2020**, *2* (1), 56–67. https://doi.org/10.1038/s42256-019-0138-9.

(9) Zhou, Q.; Kunder, N.; De La Paz, J. A.; Lasley, A. E.; Bhat, V. D.; Morcos, F.; Campbell, Z. T. Global Pairwise RNA Interaction Landscapes Reveal Core Features of Protein Recognition. *Nature Communications* **2018**, *9* (1), 1–10. https://doi.org/10.1038/s41467-018-04729-0.

(10) Ekeberg, M.; Lövkvist, C.; Lan, Y.; Weigt, M.; Aurell, E. Improved Contact Prediction in Proteins: Using Pseudolikelihoods to Infer Potts Models. *Physical Review E - Statistical, Nonlinear, and Soft Matter Physics* **2013**, *87* (1), 1–16. https://doi.org/10.1103/PhysRevE.87.012707.

(11) Balakrishnan, S.; Kamisetty, H.; Carbonell, J. G.; Lee, S. I.; Langmead, C. J. Learning Generative Models for Protein Fold Families. *Proteins: Structure, Function and Bioinformatics* **2011**, *79* (4), 1061–1078. https://doi.org/10.1002/prot.22934.

(12) Ravikumar, P.; Wainwright, M. J.; Lafferty, J. D. High-Dimensional Ising Model Selection Using ℓ1-Regularized Logistic Regression. *Annals of Statistics* **2010**, *38* (3), 1287–1319. https://doi.org/10.1214/09-AOS691.

(13) Hunter, J. D. Matplotlib: A 2D Graphics Environment. *Computing in Science and Engineering* **2007**, *9* (3), 90–95. https://doi.org/10.1109/MCSE.2007.55.

(14) Bachschmid-Romano, L.; Opper, M. A Statistical Physics Approach to Learning Curves for the Inverse Ising Problem. *Journal of Statistical Mechanics: Theory and Experiment* **2017**, *2017* (6). https://doi.org/10.1088/1742-5468/aa727d.

(15) Fanthomme, A.; Rizzato, F.; Cocco, S.; Monasson, R. Optimal Regularizations for Data Generation with Probabilistic Graphical Models. *Journal of Statistical Mechanics: Theory and Experiment* **2022**, *2022* (5). https://doi.org/10.1088/1742-5468/ac650c.

(16) Mazzocato, Y.; Perin, S.; Morales-sanfrutos, J.; Pluda, S.; Acquasaliente, L.; Borsato, G.; Filippis, D.; Scarso, A.; Angelini, A. A Novel Genetically-Encoded Bicyclic Peptide Inhibitor of Human Urokinasetype Plasminogen Activator with Better Cross-Reactivity toward the Murine Orthologue. *Bioorganic & Medicinal Chemistry* **2023**, 117499. https://doi.org/10.1016/j.bmc.2023.117499.

(17) Angelini, A.; Cendron, L.; Chen, S.; Touati, J.; Winter, G.; Zanotti, G.; Heinis, C. Bicyclic Peptide Inhibitor Reveals Large Contact Interface with a Protease Target. *ACS Chemical Biology* **2012**, *7* (5), 817–821. https://doi.org/10.1021/cb200478t.

(18) Angelini, A.; Diderich, P.; Morales-Sanfrutos, J.; Thurnheer, S.; Hacker, D.; Menin, L.; Heinis, C. Chemical Macrocyclization of Peptides Fused to Antibody Fc Fragments. *Bioconjugate Chemistry* **2012**, *23* (9), 1856–1863.

https://doi.org/10.1021/bc300184m.

(19) Angelini, A.; Miyabe, Y.; Newsted, D.; Kwan, B. H.; Miyabe, C.; Kelly, R. L.; Jamy, M. N.; Luster, A. D.; Wittrup, K. D. Directed Evolution of Broadly Crossreactive Chemokine-Blocking Antibodies Efficacious in Arthritis. *Nature Communications* **2018**, *9* (1). https://doi.org/10.1038/s41467-018-03687-x.

(20) Potterton, L.; Agirre, J.; Ballard, C.; Cowtan, K.; Dodson, E.; Evans, P. R.; Jenkins, H. T.; Keegan, R.; Krissinel, E.; Stevenson, K.; Lebedev, A.; McNicholas, S. J.; Nicholls, R. A.; Noble, M.; Pannu, N. S.; Roth, C.; Sheldrick, G.; Skubak, P.; Turkenburg, J.; Uski, V.; Von Delft, F.; Waterman, D.; Wilson, K.; Winn, M.; Wojdyr, M. CCP 4 i 2: The New Graphical User Interface to the CCP 4 Program Suite. *Acta Crystallographica Section D: Structural Biology* **2018**, *74*, 68–84. https://doi.org/10.1107/S2059798317016035.

(21) McCoy, A. J.; Grosse-Kunstleve, R. W.; Adams, P. D.; Winn, M. D.; Storoni, L. C.; Read, R. J. Phaser Crystallographic Software. *Journal of Applied Crystallography* **2007**, *40* (4), 658–674. https://doi.org/10.1107/S0021889807021206.

(22) Vagin, A. A.; Steiner, R. A.; Lebedev, A. A.; Potterton, L.; McNicholas, S.; Long, F.; Murshudov, G. N. REFMAC5 Dictionary: Organization of Prior Chemical Knowledge and Guidelines for Its Use. *Acta Crystallographica Section D: Biological Crystallography* **2004**, *60* (12 I), 2184–2195. https://doi.org/10.1107/S0907444904023510.

(23) Adams, P. D.; Afonine, P. V.; Bunkóczi, G.; Chen, V. B.; Davis, I. W.; Echols, N.; Headd, J. J.; Hung, L. W.; Kapral, G. J.; Grosse-Kunstleve, R. W.; McCoy, A. J.; Moriarty, N. W.; Oeffner, R.; Read, R. J.; Richardson, D. C.; Richardson, J. S.; Terwilliger, T. C.; Zwart, P. H. PHENIX: A Comprehensive Python-Based System for Macromolecular Structure Solution. *Acta Crystallographica Section D: Biological Crystallography* **2010**, *66* (2), 213–221. https://doi.org/10.1107/S0907444909052925.

(24) Emsley, P.; Lohkamp, B.; Scott, W. G.; Cowtan, K. Features and Development of Coot. *Acta Crystallographica Section D: Biological Crystallography* **2010**, *66* (4), 486–501. https://doi.org/10.1107/S0907444910007493.

(25) Krissinel, E.; Henrick, K. Inference of Macromolecular Assemblies from Crystalline State. *Journal of Molecular Biology* **2007**, *372* (3), 774–797. https://doi.org/10.1016/j.jmb.2007.05.022.

(26) Laskowski, R. A.; Jabłońska, J.; Pravda, L.; Vařeková, R. S.; Thornton, J. M. PDBsum: Structural Summaries of PDB Entries. *Protein Science* **2018**, *27* (1), 129–134. https://doi.org/10.1002/pro.3289.

(27) Roman, A. L.; Mark, B. S. LigPlot+: Multiple Ligand-Protein Interaction Diagrams for Drug Discovery. *Journal of Chemical Information and Modeling* **2011**, *51*, 2778–2786.

(28) The PyMOLMolecularGraphics,Version2.0;Schrödinger,LLC. https://doi.org/10.1002/chem.201702117.

(29) Barthels, F.; Schirmeister, T.; Kersten, C. BANΔIT: B'-Factor Analysis for Drug Design and Structural Biology. *Molecular Informatics* **2021**, *40* (1), 1–6. https://doi.org/10.1002/minf.202000144.
